# Supplementary material for: Examining the application of the IDEAL framework in the reporting and evaluation of innovative invasive procedures: secondary qualitative analysis of a systematic review
Source: BMJ Open. 2024 May 24;14(5):e079654. doi: 10.1136/bmjopen-2023-079654 (PMC11129025; doi:10.1136/bmjopen-2023-079654)
Supplement: Supplementary data [file bmjopen-2023-079654supp002.pdf]

Supplementary Material 2

Search strategy:

The search strategy was to identify article that had cited at least one of the ten key IDEAL publications (see below) using the electronic citation tools in SCOPUS and Web of Science.

Electronic searches in these databases (web of Science and Scopus) were performed to identify studies reported as following the IDEAL/IDEAL-D framework. This was defined as a study citing any of the ten key IDEAL/IDEAL-D papers that members of the IDEAL collaboration deemed significant in describing the framework (see Khachane *et al*, 2018). No restrictions to study design or publication dates were applied. Searches were conducted in April 2019, and were updated in June 2020.

SCOPUS
Search date: 1st April 2019
1112 records identified.

Searches repeated: 26th June 2020 (limited to since 01/04/2019)
259 records identified.

Web of Science
Search date: 1st April 2019
1018 records identified.

Searches repeated: 26th June 2020 (limited to since 01/04/2019)
200 records identified.

Citation searches were conducted for the following papers:

1. McCulloch P, Altman DG, Campbell WB, Flum DR, Glasziou P, Marshall JC et al. No surgical innovation without evaluation: the IDEAL recommendations. Lancet 2009; 374: 1105–1112.
2. Sedrakyan A, Campbell B, Merino JG, Kuntz R, Hirst A, McCulloch P. IDEAL-D: a rational framework for evaluating and regulating the use of medical devices. BMJ 2016; 353: i2372.
3. Barkun JS, Aronson JK, Feldman LS, Maddern GJ, Strasberg SM, Altman DG et al. Evaluation and stages of surgical innovations. Lancet 2009; 374: 1089–1096.
4. Ergina PL, Cook JA, Blazeby JM, Boutron I, Clavien PA, Reeves BC et al. Challenges in evaluating surgical innovation. Lancet 2009; 374: 1097–1104.
5. Cook JA, McCulloch P, Blazeby JM, Beard DJ, Marinac-Dabic D, Sedrakyan A; IDEAL Group. IDEAL framework for surgical innovation 3: randomised controlled trials in the assessment stage and evaluations in the long term study stage. BMJ 2013; 346: f2820.
6. Ergina PL, Barkun JS, McCulloch P, Cook JA, Altman DG; IDEAL Group. IDEAL framework for surgical innovation 2: observational studies in the exploration and assessment stages. BMJ 2013; 346: f3011.
7. Hirst A, Agha RA, Rosin D, McCulloch P. How can we improve surgical research and innovation?: the IDEAL framework for action. Int J Surg 2013; 11: 1038–1042.

8. McCulloch P, Cook JA, Altman DG, Heneghan C, Diener MK; IDEAL Group. IDEAL framework for surgical innovation 1: the idea and development stages. BMJ 2013; 346: f3012.
9. Pennell CP, Hirst A, Sedrakyan A, McCulloch PG. Adapting the IDEAL framework and recommendations for medical device evaluation: a modified Delphi survey. Int J Surg 2016; 28: 141–148.
10. Pennell CP, Hirst AD, Campbell WB, Sood A, Agha RA, Barkun JS et al. Practical guide to the idea, development and exploration stages of the IDEAL framework and recommendations. Br J Surg 2016; 103: 607–615.
