# Supplementary figures and images for: Examining the application of the IDEAL framework in the reporting and evaluation of innovative invasive procedures: secondary qualitative analysis of a systematic review
Source: BMJ Open. 2024 May 24;14(5):e079654. doi: 10.1136/bmjopen-2023-079654 (PMC11129025; doi:10.1136/bmjopen-2023-079654)

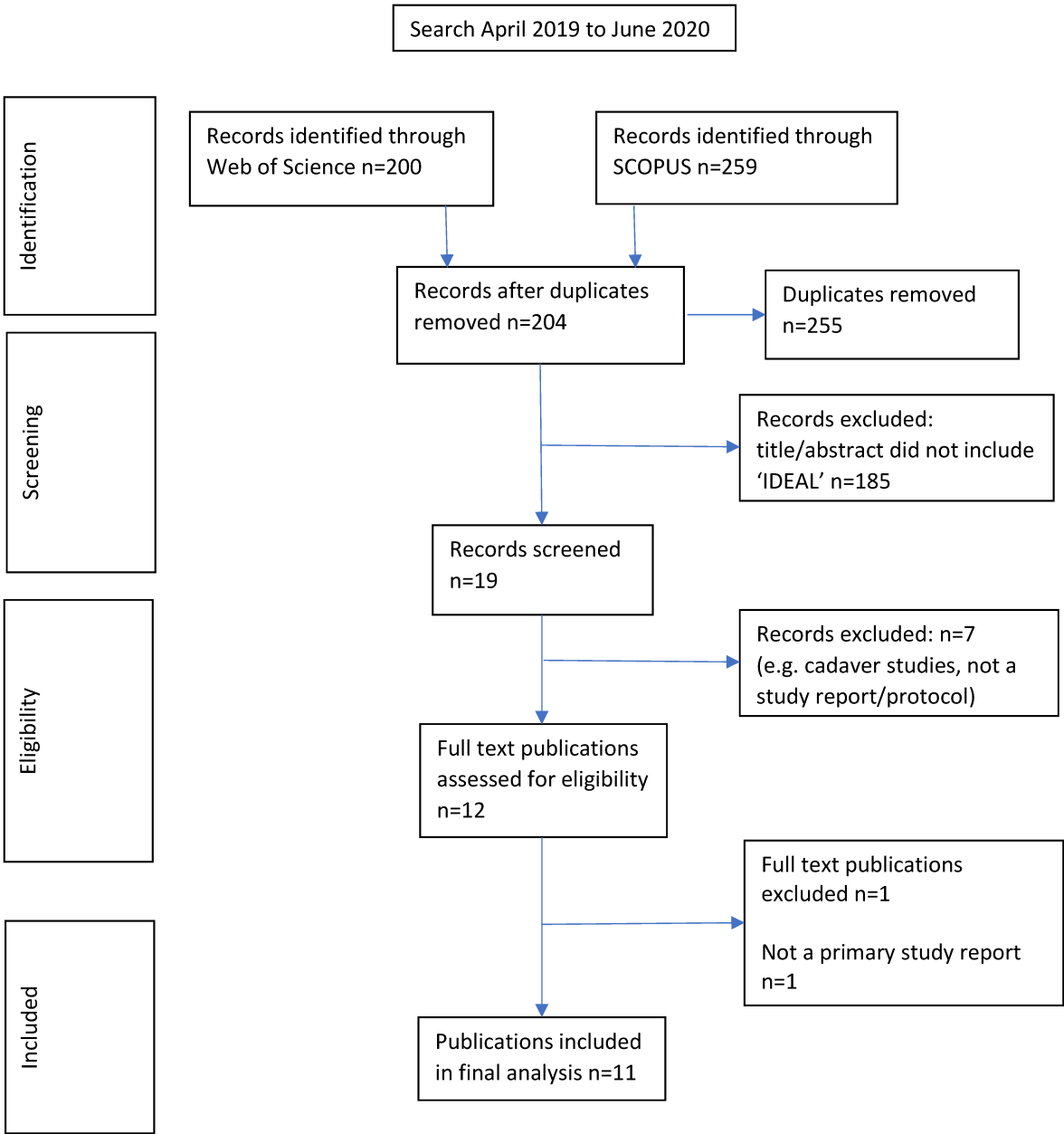

Supplement: Supplementary data [file bmjopen-2023-079654supp003.pdf]
