# Supplementary material for: Examining the application of the IDEAL framework in the reporting and evaluation of innovative invasive procedures: secondary qualitative analysis of a systematic review
Source: BMJ Open. 2024 May 24;14(5):e079654. doi: 10.1136/bmjopen-2023-079654 (PMC11129025; doi:10.1136/bmjopen-2023-079654)
Supplement: Author's manuscript [file bmjopen-2023-079654.draft_revisions.pdf]

BMJ Open is committed to open peer review. As part of this commitment we make the peer review history of every article we publish publicly available.

When an article is published we post the peer reviewers' comments and the authors' responses online. We also post the versions of the paper that were used during peer review. These are the versions that the peer review comments apply to.

The versions of the paper that follow are the versions that were submitted during the peer review process. They are not the versions of record or the final published versions. They should not be cited or distributed as the published version of this manuscript.

BMJ Open is an open access journal and the full, final, typeset and author-corrected version of record of the manuscript is available on our site with no access controls, subscription charges or pay-per-view fees (http://bmjopen.bmj.com).

If you have any questions on BMJ Open's open peer review process please email info.bmjopen@bmj.com

BMJ Open

Examining the application of the IDEAL Framework: a systematic review and qualitative analysis of the reporting and evaluation of innovative invasive procedures in the published literature

Journal:	*BMJ Open*
Manuscript ID	bmjopen-2023-079654
Article Type:	Original research
Date Submitted by the Author:	07-Sep-2023
Complete List of Authors:	Richards, Hollie; University of Bristol Medical School, Population Health Sciences<br>Cousins, Sian; University of Bristol<br>Scroggie, Darren; University of Bristol<br>Elliott, Daisy; University of Bristol<br>Macefield, Rhiannon; University of Bristol Medical School, Population Health Sciences<br>Alford, Natasha; University of Bristol Medical School<br>Shah, Maximilian; University of Bristol Medical School<br>Blencowe, Natalie; University of Bristol Medical School, Population Health Sciences<br>Blazeby, Jane; University of Bristol Medical School, Population Health Sciences
Keywords:	SURGERY, QUALITATIVE RESEARCH, Systematic Review

SCHOLARONE™
Manuscripts

I, the Submitting Author has the right to grant and does grant on behalf of all authors of the Work (as defined in the below author licence), an exclusive licence and/or a non-exclusive licence for contributions from authors who are: i) UK Crown employees; ii) where BMJ has agreed a CC-BY licence shall apply, and/or iii) in accordance with the terms applicable for US Federal Government officers or employees acting as part of their official duties; on a worldwide, perpetual, irrevocable, royalty-free basis to BMJ Publishing Group Ltd ("BMJ") its licensees and where the relevant Journal is co-owned by BMJ to the co-owners of the Journal, to publish the Work in this journal and any other BMJ products and to exploit all rights, as set out in our licence.

The Submitting Author accepts and understands that any supply made under these terms is made by BMJ to the Submitting Author unless you are acting as an employee on behalf of your employer or a postgraduate student of an affiliated institution which is paying any applicable article publishing charge ("APC") for Open Access articles. Where the Submitting Author wishes to make the Work available on an Open Access basis (and intends to pay the relevant APC), the terms of reuse of such Open Access shall be governed by a Creative Commons licence – details of these licences and which Creative Commons licence will apply to this Work are set out in our licence referred to above.

Other than as permitted in any relevant BMJ Author's Self Archiving Policies, I confirm this Work has not been accepted for publication elsewhere, is not being considered for publication elsewhere and does not duplicate material already published. I confirm all authors consent to publication of this Work and authorise the granting of this licence.

For peer review only

Examining the application of the IDEAL Framework: a systematic review and qualitative analysis of the reporting and evaluation of innovative invasive procedures in the published literature

[1]Richards, H.S*., [1]Cousins, S., [1]Scroggie, D., [1]Elliott, D., [1]Macefield, R., [1]Hudson, E., [1]Mutanga, I., [1]Shah, M., [1]Alford, N., [1]Blencowe, N.S & [1]Blazeby, J.M.

[1]National Institute for Health and Care Research Bristol Biomedical Research Centre, University Hospitals Bristol and Weston NHS Foundation Trust and University of Bristol, Bristol, United Kingdom.

*Corresponding author: Bristol Medical School: Population Health Sciences, University of Bristol, 39 Whatley Road, Bristol, BS8 2PS, United Kingdom. Email: hollie.richards@bristol.ac.uk,

ABSTRACT

Introduction

The development of new surgical procedures, techniques and devices is fundamental to advancing patient care. The IDEAL framework describes study designs for four stages of innovation (first in human to long term follow up). It can be difficult to apply due to challenges in defining and identifying innovation in surgery. This study aimed to examine how the IDEAL framework is operationalised in real world settings; specifically, what types of innovations are evaluated and reported using the framework and how authors justify their choice of IDEAL study design.

Methods

Papers self-described as IDEAL studies, citing at least one of the 10 key IDEAL papers, were examined using a thematic framework approach. Three frameworks were developed: i) type of innovation under evaluation; ii) terminology used to describe stage of innovation; and iii) reported rationale for IDEAL stage.

Results

Forty-eight articles were included. 19/48 papers described innovations of an entirely new procedure, most commonly procedures being used for the first time in a different clinical context (n=15/48), reported as IDEAL stage 2a (n=8, 53%). Terminology used to describe stage of innovation was varied,

For peer review only - http://bmjopen.bmj.com/site/about/guidelines.xhtml

inconsistent and ambiguous. Terms such as 'innovation' were not defined. Authors commonly justified their choice of IDEAL study design based on limitations in published evidence (n=36) and unknown feasibility and safety (n=32) outcomes.

Conclusion

Identifying stage of innovation is crucial to inform appropriate study design and governance decisions. Authors' rationale for choice of IDEAL stage often related to the existing evidence base or lack of sufficient outcome data for procedures. Stage of innovation was poorly defined using vague and inconsistent descriptions. Further work is needed to develop methods to identify innovation to inform practical application of the IDEAL framework. Defining the concept of innovation in terms of uncertainty, risk and degree of evidence may help to inform decision making.

Strengths and limitations of this study

- This review provides an in-depth review of how the IDEAL Framework has been applied and reported by examining how authors self-report stage of innovation in relation to IDEAL and use this to inform study design decisions.
- Limitations include the omission of potentially relevant papers where authors did not explicitly reference their use of the IDEAL Framework and that database searches took place in 2020.

INTRODUCTION

Innovation in healthcare requires evidence of safety and effectiveness before widespread implementation. Although pharmaceutical development occurs through a clearly delineated pathway, surgical innovation often happens haphazardly without incremental building of evidence through evaluation within formal research studies (1, 2). The IDEAL (Idea, Developments, Exploration, Assessment and Long-term follow-up) framework was developed to facilitate the evaluation of new surgical procedures and devices (IDEAL-D)(3, 4). It provides study design and governance

For peer review only

recommendations for new procedures and devices at different stages of development. Stage 0 deals with pre-clinical studies, prior to stage 1 first in human studies. This is followed by stage 2a developmental studies, which focus on technical details and ongoing modifications of the intervention, moving to stage 2b exploratory studies where the intervention and patient group are defined in multiple centres in preparation for stage 3 comparative studies. These are typically randomised controlled trials (RCTs) comparing the now stabilised intervention against standard treatment. Stage 4 consists of long-term monitoring in registries (3, 4). Although IDEAL is an important contribution to the field, it has not been widely applied (1, 5, 6) and is not always used appropriately (7-9). One challenge is that application of the recommendations requires determining the innovative stage of the procedure, usually by examining the previous literature, and that 'innovation' is not well defined (10)

Commonly, definitions of surgical innovation are intrinsically linked to concepts of 'newness', including the extent to which a procedure differs from standard practice (11) or is being modified (11-15). It can also be defined as something with uncertain levels of risk (16). Requirements for additional training (13, 15) and the degree of evidence of safety and efficacy (17, 18) have been used to determine how innovative an intervention is. However, some of these definitions are difficult to apply in practice (19). For example, it is often unclear what degree of modification constitutes innovation (10, 20). Guidance has been developed to help surgeons identify innovative procedures (21). The Macquarie Tool bases the degree of innovation on surgeons' previous experiences and how much the procedure varies from routine practice. This places the responsibility for identifying innovation on the surgeon (19), and as such may be open to individual interpretations (19, 22) and biases (23-25). IDEAL suggests that examination of published studies may help determine stage of innovation (26). Although this is useful for stage 1 proof-of-concept studies, where there is very limited or no published evidence about the innovation, the guidance acknowledges that the literature may be of limited use when interventions are at a later stage of innovation. Distinguishing IDEAL stage 2a from 2b may be problematic as centres

For peer review only - http://bmjopen.bmj.com/site/about/guidelines.xhtml

For peer review only

achieve stability in a procedure at different times (26). Given these challenges, examination of how stage of innovation is determined and used by surgeons and is needed.

The aim of this study is to examine how innovative surgical procedures are conceptualised, evaluated and reported using the IDEAL Framework and to understand authors' rationale for choice of IDEAL study design. This will inform work to develop practical methods to facilitate determination of stage of innovation, to ensure appropriate study design and governance (4, 20).

For peer review only

METHODS

Published papers self-reported as IDEAL studies, reporting evaluations of innovative surgical procedures, were examined using a qualitative approach.

Searches

Articles identified in a previous systematic review(27) were retrieved. Electronic searches of the Web of Science and Scopus databases were undertaken to identify all papers citing any of the ten key IDEAL papers (3, 7, 26, 28-34) published between January 2009 and April 2019. Records were further filtered to select only those with 'IDEAL' or 'IDEAL-D' in the title or abstract. Searches were updated in June 2020 using the same databases and search criteria to identify further studies. Articles were imported into reference management software (35) and duplicates were removed. No further searches were undertaken after 2020 because the current review focussed on authors' conceptualisation and description of innovation, and justification of their choice of study design. Such factors are unlikely to change across the time since the searches were undertaken.

Eligibility criteria and definitions

Eligibility criteria for inclusion in the previous review and updated search were studies of invasive procedures and devices of any design. Secondary studies and those not involving living human

For peer review only - http://bmjopen.bmj.com/site/about/guidelines.xhtml

participants, were excluded, along with editorials, commentaries, conference proceedings and letters (27). Further exclusion criteria for the current review were studies where the intervention under evaluation was not the invasive procedure itself (e.g. non-invasive co-interventions). Invasive procedures were defined as interventions where access to the body was gained by incision, natural orifices or endoscopic techniques, or percutaneous puncture where instrumentation is used in addition to the puncture needle (36). Invasive devices were defined as any instrument, apparatus or material that is used within or implanted into the body.

Screening

All studies included in the previous review and update were screened to ensure they met the updated eligibility criteria. Titles and abstracts were reviewed initially by one reviewer (HSR) and ineligible studies excluded. Full texts of all remaining articles were then screened. At each stage, 20% of articles were screened by a second reviewer (SC). Disagreements were resolved by discussion amongst the wider study team.

Data extraction and analysis

Study characteristics

Study characteristics were extracted using a standardised REDCap electronic data capture form (37, 38). Author-reported IDEAL stage, year of publication, country, the invasive procedure or device under evaluation, number of centres and surgeons, and sample size are reported in the previous systematic review (27). Additional details about reported oversight (e.g. research governance), study design, and surgical specialty were extracted by one reviewer (HSR). Study designs, determined by NICE definitions (39) were extracted from all papers by two independent reviewers (HSR, SC). Surgical specialty data was extracted from 20% of papers by a second independent surgeon reviewer (DS). Disagreements were resolved by discussion. Characteristics were reported using descriptive statistics, where appropriate.

For peer review only - http://bmjopen.bmj.com/site/about/guidelines.xhtml

For peer review only

Type of surgical innovation under evaluation and rationale for study design

Papers were analysed using a thematic framework approach (37, 38). This structured approach involves coding of data to identify themes to form a framework, which was applied in an iterative and reflexive manner (37, 38). Full publications were imported into NVivo (version 13). Text was coded (whereby text of interest is identified, and descriptive labels attached) using a combination of deductive and inductive approaches, allowing for any unexpected themes to develop. This ensured that analysis was data driven. Three frameworks were developed according to study aims. The first (1) related to the type of innovation under evaluation. All text describing the surgical innovation under evaluation was extracted and coded inductively. For each study, the innovative component under evaluation was identified, for example if the procedure was described as being used for the first time or if changes to a procedure were reported. Where procedural changes were reported, a typology that facilitates the deconstruction of invasive procedures into their constituent parts was utilised to identify how many, and which, steps of the procedure were changed (36). Where codes shared commonalities, these were grouped together into themes and sub-themes to form a framework. Where the innovation under evaluation involved a device or robot, these formed a separate theme. The second (2) framework related to the terminology used to describe the stage of innovation of the procedure. This was analysed according to pre-agreed themes: 'novel/new' – the first or early use of the procedure/device; 'modified' – planned or unplanned changes to the procedure/device or patient group; 'stabilised' – the finalisation of iterative changes or lack of subsequent changes/modifications; and 'adopted' – the wider use of the procedure/device in clinical practice. If the terminology describing stage of innovation could not be coded to these themes, additional themes were created. The third (3) framework related to the reported rationale for study design. All text describing the author's reported rationale for choice of study design was extracted and coded inductively. A narrative summary was produced to illustrate findings with reference to study characteristics.

For peer review only - http://bmjopen.bmj.com/site/about/guidelines.xhtml

Framework development

Three multi-disciplinary (qualitative research (HSR), health services research (SC) and surgical (DS)) members of the team independently coded an initial five articles and discussed how the text had been interpreted. This allowed similarities and differences to be incorporated into an initial set of codes and themes with clear definitions (40). This process was repeated iteratively until agreement was reached on the definitions of codes and themes/sub-themes. A senior member of the team (JMB) reviewed coded text and the iterative development of frameworks. Each framework was then applied to all articles by one researcher (HSR). The research team (HSR, SC, DC and JMB) met regularly throughout this process to discuss analysis, ensuring agreement was reached regarding the appropriate interpretation of the text.

RESULTS

From the 74 papers retrieved from the previous review (27) and update (supplementary material Figure 1), 48 were included.

For peer review only

Study characteristics		Total, n=48 (%)	Author-reported IDEAL Stage					
			1 (n=11)	2a (n=18)	2b (n=6)	3 (n=2)	Multiple (n=9)	Not stated (n=2)
Study Design	Case series	36 (75)	7	15	6	-	7	1
	Case study	6 (13)	4	1	-	-	-	1
	Cohort study	4 (8)	-	2	-	1	1	-
	Randomised trial	1 (2)	-	-	-	1	-	-
	Multiple study designs	1 (2)	-	-	-	-	1	-
Surgical Specialty	Urology	25 (52)	6	10	4	1	4	-
	Gastrointestinal surgery	7 (15)	1	2	1	1	2	-
	Colorectal surgery	5 (10)	1	2	-	-	2	-
	Gynaecology and obstetrics	2 (4)	1	-	1	-	-	-
	Breast surgery	3 (6)	-	2	-	-	-	1
	Otolaryngology	2 (4)	-	2	-	-	-	-
	Maxillofacial and dental	2 (4)	2	-	-	-	-	-
	Orthopaedics	1 (2)	-	-	-	-	1	-
	Paediatric	1 (2)	-	-	-	-	-	1
Country	European Economic Area (Non-UK)	30 (63)	6	11	6	1	5	1
	Other[1]	9 (19)	2	3	-	1	3	-
	UK	8 (16)	2	4	-	-	1	1
	North America	1 (2)	1	-	-	-	-	-
Publication year	2011 - 2015	12 (25)	3	4	-	-	3	-
	2016 - 2020	36 (75)	8	14	6	2	6	-
Single or multi-centre study	Single centre	41 (85)	11	15	5	1	7	2
	Multi centre	5 (10)	-	2	1	1	1	-
	Not reported	2 (4)	-	1	-	-	1	-
Number of surgeons performing the intervention	1 to 2	22 (46)	6	10	-	2	3	1
	3 to 7	5 (10)	-	2	2	-	1	-
	Not reported/Unclear	21 (44)	5	6	4	-	5	1
Number of participants	1-10	10 (21)	7	2	-	-	-	1
	11-50	26 (54)	4	14	2	-	5	1
	51-100	5 (10)	-	1	1	-	3	-
	101-400	5 (10)	-	1	2	1	1	-
	401-510	2 (4)	-	-	1	1	-	-

Table 1. Characteristics of included studies.

For peer review only

For peer review only - http://bmjopen.bmj.com/site/about/guidelines.xhtml

Study characteristics

Characteristics of included publications are detailed in Table 1. The majority of studies were case series (n=36, 75%) or case studies (n=6, 13%). Most studies were reported as IDEAL stage 2a (n=18) although two did not explicitly state a stage: one stated it was stage 1 or 2a (41) and the other self-reported as an early-stage assessment of technical problems during the learning curve "in line with the IDEAL recommendations" (42).

Table 2 details reported governance arrangements. Eighteen (36%) reported research governance, including approval from Institutional Review Boards or Research Ethics Committees overseen by the U.S. Food and Drug Administration (FDA) or the U.K NHS Health Research Authority (HRA). Most of these were reported as stage 2a (n=7). Thirteen (27%) papers reported obtaining local/institutional approval, using a variety of terms such as "local ethical committee" and "institutional medical ethical committee" to describe them. Five (10%) papers, including three reported as IDEAL stage 1, stated that ethical approval was not required or obtained. The remaining twelve (25%) papers, including IDEAL stage 1 (n=3) and stage 2a (n=5), did not report any governance approvals.

For peer review only

Reported governance approvals		Total (%) N=48	Author-reported IDEAL Stage					
			1 (n=11)	2a (n=18)	2b (n=6)	3 (n=2)	Multiple (n=9)	Not stated (n=2)
Formal research governance	Institutional Review Board (IRB)	14	4	5	2	1	2	-
	"REC ethical approval"	1	-	1	-	-	-	-
	"REC ethical approval (& NPC)"	1	-	1	-	-	-	-
	"Regional ethics commissions"	1	-	-	-	1	-	-
	"National ethics committee"	1	-	-	1	-	-	-
Local or institutional approvals	"Local ethical committee"	2	-	1	1	-	-	-
	"Local ethics board"	1	-	-	-	-	1	-
	"Local ethics review committee"	1	-	1	-	-	-	-
	"Local medical ethical committee"	2	-	1	1	-	-	-
	"Local research ethics committee"	3	1	1	-	-	1	-
	"Institution ethical committee"	1	-	1	-	-	-	-
	"Institutional health sciences research	1	-	-	-	-	1	-
	"Institutional medical ethical	1	-	-	-	-	1	-
	"Ethical commission of medical	1	-	-	-	-	1	-
No ethical approval obtained	"Current study: no ethical approval."	1	-	1	-	-	-	-
	"Audit - ethics not required"	1	-	-	-	-	1	-
	"Ethical approval not required"	1	1	-	-	-	-	-
	"No further ethics approval	1	1	-	-	-	-	-
	"Not needed due to retrospective	1	1	-	-	-	-	-
Not reported	N/A	12	3	5	1	-	1	2

Table 2. Governance approvals reported by 48 included studies.

For peer review only - http://bmjopen.bmj.com/site/about/guidelines.xhtml

(1) Type of innovation under evaluation

The types of innovations under evaluation were categorised into five themes related to 'newness' (Table 3); i) an entire new procedure (this included if a procedure had two or more new steps; n=19); ii) one step of the procedure is new; iii) a new device including robotic approach; iv) changes to the sequence of procedure components; and v) changes to the combination of procedure components.

i) An entire new procedure

Nineteen (39%) papers reported an entire new procedure. Most (n=15) referred to procedures being newly used in a different clinical context, for example, combining existing minimally invasive techniques into a new type of reconstruction or anastomosis in bowel (43) and urological (44) surgery. Of these, 8 were classified as IDEAL 1a. Three (6%) other papers described procedures being applied to a different clinical indication, for example high intensity focused ultrasound applied to focal therapy for prostate tumours (45).

ii) One step of the procedure is new

Nineteen other papers described procedures with one step changed. Most (n=8) were stage 2a. Ten involved one step of a procedure being applied to different clinical indication, for example an existing invasive imaging technique currently used for one type of cancer being used for a different type of cancer (46-50). Six described changes to access to a body cavity by using minimally invasive techniques instead of open surgery (41, 51-55). One paper described the application of one step of an invasive procedure to a different patient group (56).

iii) A new device including robotic approach

Eight (17%) papers described the evaluation of an invasive device/robot. Of these, four described changes to one component of an invasive device (n=1) or robot (n=3). These were a modification to the coating of a vaginal implant device (57), a new access port for use with a robot devices (58, 59) and changing part of the procedure from open to robotic (60). These were reported as IDEAL stage 1 (n=2), stage 2a (n=1) or stage 3 (n=1) respectively. Three papers described the development of an entirely new invasive device (n=1) or robot (n=2). (61).

For peer review only - http://bmjopen.bmj.com/site/about/guidelines.xhtml

Main theme	Subthemes (if applicable)	Total (n=48)	Author-reported IDEAL stage and number of papers with text coded to theme					
			1 (n=11)	2a (n=18)	2b (n=6)	3 (n=2)	Multiple Stages (n=9)	Not stated (n=2)
An entire new procedure	Procedure used for the first time in that clinical context	15 (31%)	3	8	-	-	3	1
	Procedure applied to a different clinical indication	3 (6%)	1	-	1	-	1	-
	Procedure previously used in different patient group	1 (2%)	-	-	1	-	-	-
One step of the procedure is new	Step of procedure applied to a different clinical indication	10 (21%)	-	7	2	-	1	-
	Change to access	6 (12%)	1	-	1	1	2	1
	Change to reconstruction	2 (4%)	1	1	-	-	-	-
	Step of a procedure previously used in different patient group	1 (2%)	1	-	-	-	-	-
A new device including robotic approach	Change to one component of a device/robotic instruments	4 (8%)	2	1	-	1	-	-
	Development of an entire device/robotic instruments for one component of procedure	3 (6%)	1	1	-	-	1	-
	Existing device applied to a different clinical indication	1 (2%)	1	-	-	-	-	-
Novel changes to the sequence of procedure components	N/A	1 (2%)	-	-	-	-	1	-
Changes to the combination of procedure components	N/A	1 (2%)	-	-	1	-	-	-

Table 3. Main themes and sub-themes describing the type of innovation, by author-reported IDEAL stage and in total.

iv) Changes to the sequence of procedure components

One paper, reported as a sequential IDEAL stages 2a and 2b study, evaluated changes to the timing of radiotherapy for unstable spinal metastases (62).

v) Changes to the combination of procedure components

One IDEAL stage 2b study described an evaluation of combined versus individual invasive diagnostic tests (63).

(2) Terminology used to describe stage of innovation

In addition to the four *a priori* themes, the theme of abandoned procedures was identified during data analyses. The final five themes, (i) new/novel; ii) modified; iii) stabilised; iv) adopted; and v) abandoned procedures, are summarised below.

i) New/novel

There was a lack of consistency across papers reporting all IDEAL stages in terms describing the 'newness' of the procedure. Commonly terms included 'innovation' (n=18), 'new' (n=14), 'novel' (n=13), 'introduction' (n=4) and 'first-in-human' (n=4). 'Innovation' was used in papers reporting all IDEAL stages, whereas 'introduction' was predominantly used in stage 1 papers (n=3). None of the papers defined these terms. 'First-in-human' was used in one IDEAL stage 1 study, one stage 3 and two multistage studies, but in these latter cases it described the earlier development of the innovation under evaluation. Twenty studies used terminology and phrases related to the first report of the innovation (e.g. "this is the first study" (64), "first clinical trial" (65) and "the first case" (66)). The majority of these were stage 1 (n=7, 16%) and stage 2a (n=8, 17%). Five papers described the procedure as "emerging" (41) or "promising" (67). These were reported as IDEAL stage 2a (n=3), 2b (n=1) or not stated (n=1).

ii) Modified

For peer review only - http://bmjopen.bmj.com/site/about/guidelines.xhtml

For peer review only

Twenty-three papers, mostly early stage 1-2b, used a variety of terms to describe modifications. Although the terms ‘modified’ or ‘modification’ were used in eleven (23%) papers, most (n=18, 38%) described modifications in terms of the evolution and ongoing development of the procedure. This included the “evolution of the new method” (68), describing the procedure as “still under development” (69). Ten (21%) papers described changes based on their experience, e.g. “from case 35 onward” (61), “based on the results of patient 1” (54) and “thereafter we noticed” (58). These tended to appear in stage 2a (n=4) and stage 1 (n=3) studies. Some papers used more precise terms, such as ‘refinements’ (n=7, 16%), ‘changes’ (n=5, 11%) and ‘adaptions/adjustments/alterations’ (n=4, 8%). Others were vaguer. Terms included “hybrid solutions” (58) and “continued…optimization” (70). Additional undefined terms for modifications included “experimented” (71) and “improvised” (72), and descriptions of steps of the procedure being “replaced” (55) or “tried [and] tested” (72).

iii) Stabilised

Eight papers described the procedure using terms synonymous with stabilised (n=2 stage 1; n=3 stage 2a; n=1 stage 3; n=2 multiple stages). Terms and phrases included having “achieved stability” (70), “appear[ing] stable” (69) or “standardized” (58). Some authors were slightly more specific and described when procedures were no longer undergoing modifications, in two cases explicitly stating that this was an indication of stabilisation as “all surgeons began learning” the procedure (51) or it “had entered IDEAL stage 3” (55).

iv) Adopted

Four papers described the procedure being adopted into clinical practice (n=2 stage 2a; n=1 stage 3; n=1 multiple stages). One said the procedure was “an established technique in our unit” (73) and another said the procedure “may become a standard” (46) upon wider adoption.

v) Abandoned

For peer review only - http://bmjopen.bmj.com/site/about/guidelines.xhtml

Three papers stated that the procedure had been abandoned (n=1 stage 2a; n=2 multiple stages), including descriptions of the procedure as “not a viable alternative” (64) or as being halted due to “loss of confidence” (51).

(3) Reported rationale for study design

Three main themes were identified (Table 4): i) the limitations and quality of previously published evidence; ii) unknown feasibility and safety outcomes; and iii) previous use in clinical practice.

(i) The limitations and quality of published evidence

Thirty-six papers described the limitations and quality of previously published evidence as a rationale for the current study design. Of these, 31 discussed the limited number of published studies evaluating the procedure. These authors highlighted the small numbers of participants and surgeons in previous studies (e.g. “treated 18 patients” (74) and “a few expert surgeons” (75)). Fifteen other papers highlighted limitations in the types of outcomes collected in previous studies and three (74, 76, 77) (6%) (n=2 stage 2a; n=1 stage 2b) highlighted a lack of long-term outcomes as rationale for the current study. Three papers (44, 55, 78) (stage 1, stage 2a and stage 2b) stated that the procedure was not standardised. Other papers (n=13) focused on previous study designs and stated that there were few prospective studies, and that most evidence was from case series, case control studies, proof-of-concept or animal model studies. Of these, 10 were reported as stage 2a (n=8) or stage 1 (n=2) studies. In addition, four papers (48, 69, 75, 79) highlighted that findings from previous studies were inconsistent, inconclusive or conflicting, suggesting further evaluation was required. Another two stage 1 papers (54, 56) commented on potential biases in previous studies, specifically gender bias, and noted that publication bias had potentially limited unfavourable reports of the procedure under evaluation, justifying their repeat evaluation in a stage 1 study.

For peer review only - http://bmjopen.bmj.com/site/about/guidelines.xhtml

Main theme	Subthemes (if applicable)	Total (n=48)[1]	Author-reported IDEAL stage and number of papers with text coded to theme					
			1 (n=11)	2a (n=18)	2b (n=6)	3 (n=2)	Multiple Stages (n=9)	Not stated (n=2)
Limitations and quality of published evidence	Limited numbers of published studies and small sample sizes	31 (65%)	6	13	5	2	3	2
	Limited outcomes reported	15 (%)	3	5	3	-	3	1
	Limitations of study designs	13 (%)	2	6	1	1	2	1
	Limitations in quality of previous studies	4 (8%)	-	2	1	-	-	1
	Biases in previous studies	2 (4%)	2	-	-	-	-	-
Unknown feasibility and safety outcomes of the procedure	Unknown feasibility outcomes	25 (%)	5	12	2	-	4	2
	Unknown risk or safety outcomes	25 (%)	6	10	3	-	4	2
	Study demonstrates proof of concept for use in humans	2 (4%)	-	-	-	-	2	-
	Outcomes used to determine progression of IDEAL Stage	1 (2%)	-	-	-	-	1	-
Previous use of the procedure in clinical practice	Procedure established in local hospital	2 (4%)	-	1	-	-	1	-
	Procedure is widely used in clinical practice	1 (2%)	-	-	1	-	-	-

[1]Studies may appear in multiple themes.

Table 4. Main themes and sub-themes describing the reported rationale for author reported study design and IDEAL stage.

For peer review only - http://bmjopen.bmj.com/site/about/guidelines.xhtml

(ii) Unknown feasibility and safety outcomes of the procedure

Thirty-two papers highlighted unknown feasibility and safety outcomes. Twenty-five specifically discussed feasibility, including thirteen that stated establishing technical feasibility was the main aim or primary outcome for the current study. These were IDEAL stage 2a (n=7), stage 1 (n=2), stage 2b (n=2) and multistage (n=2) studies. Twenty-five other papers described unknown risk or safety outcomes, the majority of which were stage 2a (n=10) and stage 1 (n=6). Two (80, 81) papers described establishing proof of concept and use in humans, both of which were reported as multiple IDEAL stages, including stage 1. One paper (51), which reported multiple studies across IDEAL stages, used the emergence of safety outcome data as rationale to progress through the IDEAL stages.

(iii) Previous use of the procedure in clinical practice

Three papers described the extent to which procedure had been used in clinical practice, including being "an established technique in our unit" (73) or as "gaining popularity" and as "increasingly being used…worldwide" (55). Another paper (80), reporting multiple IDEAL stages 1 to 3, described the need to evaluate the procedure against standard practice following its adoption by a second centre.

Discussion

In this in-depth examination of how the IDEAL framework has been applied in the published literature, we found that most papers reported early IDEAL stage 1 or 2a case series or case studies, reflecting similar findings from other recent literature reviews examining the use of the IDEAL Framework (8, 9). In these papers, reporting of entirely new procedures or those with substantial modifications were included. Governance approvals were varied and inconsistent across IDEAL stages. IDEAL recommendations specify that research ethical approval is required for stages 1-3. (4). However, around a third of papers did not report seeking any governance approvals, and 13

For peer review only - http://bmjopen.bmj.com/site/about/guidelines.xhtml

reported local or institutional approval, the processes and criteria for which are unclear. This has potential implications for patient harm and transparent consent procedures. Terminology used throughout papers varied and was inconsistent. Terms such as 'innovation' and 'modification' were used without supporting definitions. Some papers described the same procedure as both new, modified, and established (73, 80). Rationales for choice of study design were mainly based on published evidence or the need to assess safety or feasibility outcomes, in line with IDEAL recommendations (4). However, there was some inconsistency with IDEAL recommendations, such as citing the lack of long-term outcomes for the interventions as a reason for evaluating procedures as stage 2a or 2b, despite recommendations that these should measure safety, procedural and short-term outcomes (4). Clearer, more practically applicable methods are needed to identify the stage of innovation to inform study design and governance decisions.

The IDEAL Framework advises that published literature be examined to identify IDEAL stage for an intervention (26). The inconsistent and ambiguous language used to describe stage of innovation highlighted in the current review may impede this as there is disparity between author's reported IDEAL stage and the terminology used to describe the intervention. Ives *et al* (16) recommend a change in terminology used to instead reflect the level of risk associated with an intervention based on objective assessments of existing evidence. Existing definitions of 'innovation' have been described as overly broad (82) and lacking in practical utility because they do not distinguish between expected variation in standard clinical practice and changes made to procedures that are evaluated within research (24). The term 'innovation' is synonymous with positive progress and benefit (16, 19), and this may be a factor in researchers' choice of terminology for describing interventions as positive-outcome-bias is a recognised limitation in biomedical research (83-85). This bias may extend to a preference amongst journal editors to publish more eye-catching studies (86, 87). When introducing or evaluating innovations, instead of a focus on 'how new is this procedure?', a more appropriate question may be 'how does this change affect the procedure in terms of risks

For peer review only - http://bmjopen.bmj.com/site/about/guidelines.xhtml

and subsequent impact on outcomes?' (16, 19). Defining interventions in terms of degree of risk and evidence of safety and effectiveness, rather than undefined vague language (16, 19) would promote safe and transparent innovation by facilitating the appropriate choice of study design and governance.

In line with recommendations, authors commonly referred to the degree of published evidence as underpinning their choice of study design and IDEAL stage. However, many developments in surgery are not reported in the literature as they are conducted outside of the research framework (17, 88-91). Future work is needed to utilise methods that provide objective assessments of published literature and additional sources (92). This may be through systematic application of frameworks such as GRADE (Grading of Recommendations, Assessment, Development, and Evaluations)(93), for developing and summarising evidence, and by ensuring that a body of evidence is built through an incremental approach from case reports and series through to evidence synthesis in systematic reviews. This is essential to ensure patient safety and shared decision making, share learning and inform evidence based clinical decision making.

Strength and limitations

This systematic review benefits from a robust search and analysis strategy undertaken by a multidisciplinary team of researchers and clinicians. The screening process may have excluded papers which used the IDEAL Framework but did not describe an IDEAL stage in the title or abstract. However, the number of papers identified in this review is comparable to a similar review evaluating the use of the IDEAL framework in study design in paper published between 2009 and 2017 (8). Our searches took place in June 2020, more than two years prior to publication. However, since 2020, the Covid-19 pandemic has impacted the progression of many surgical studies (94-96) and the delivery of surgical interventions (97, 98). As such it is unlikely that the volume of IDEAL publications since our searches took place would be of the levels ordinarily expected within that timeframe.

For peer review only - http://bmjopen.bmj.com/site/about/guidelines.xhtml

Additionally, as this systematic review focusses on the methodology, rationale and terminology used within papers self-reporting as IDEAL stage studies, these factors are not as time dependent as those reviews that examine treatment effects.

Conclusion

This study summarises how the IDEAL framework has been applied in 'real-world' settings for a variety of interventions. It identifies inconsistencies in how authors conceptualise and describe innovation, and how they rationalise their choice of IDEAL stage and study design. Undefined terms were used to describe characteristics of the stage of innovation. It is suggested that innovation in surgery may be better conceptualised in terms of degree of evidence and magnitude of uncertainty about risk and clinical outcomes rather than the nature of the procedure itself. Future work is necessary to develop methods to facilitate this in practical terms and to inform future updates to the IDEAL framework.

Funding:

This study was funded by the National Institute for Health and Care Research Bristol Biomedical Research Centre. The views expressed are those of the author(s) and not necessarily those of the NIHR or the Department of Health and Social Care.

Authors' contributions:

J.M.B and N.B conceived the project; R.M, E.H, I.M, M.S & N.A developed and performed systematic review searches and screening; H.S.R, S.C, D.S, D.E & J.M.B developed the analysis approach and performed analysis. H.S.R & S.C. wrote the first draft of the manuscript with substantial input from J.M.B, D.S & D.E. H.S.R, S.C, D.S, D.E, R.M, N.B & J.M.B reviewed and reiterated the final manuscript.

Disclosure: The authors declare no conflicts of interest

For peer review only - http://bmjopen.bmj.com/site/about/guidelines.xhtml

Acknowledgements:

The authors acknowledge Christin Hoffman, Shelley Potter and Kerry Avery for their contribution to supervising M.S & N.A. We would also like to thank Elizabeth Hudson and Ian Mutanga for their contributions to screening.

REFERENCES

1. Kirkham EN, Main BG, Jones KJB, Blazeby JM, Blencowe NS. Systematic review of the introduction and evaluation of magnetic augmentation of the lower oesophageal sphincter for gastro-oesophageal reflux disease. Br J Surg. 2020;107(1):44-55.
2. Pathak S, Main BG, Blencowe NS, Rees JRE, Robertson HF, Abbadi RAG, et al. A Systematic Review of Minimally Invasive Trans-thoracic Liver Resection to Examine Intervention Description, Governance, and Outcome Reporting of an Innovative Technique. Ann Surg. 2020.
3. Sedrakyan A, Campbell B, Merino JG, Kuntz R, Hirst A, McCulloch P. IDEAL-D: a rational framework for evaluating and regulating the use of medical devices. Bmj. 2016;353:i2372.
4. Hirst A, Philippou Y, Blazeby J, Campbell B, Campbell M, Feinberg J, et al. No Surgical Innovation Without Evaluation: Evolution and Further Development of the IDEAL Framework and Recommendations. 2019;269(2):211-20.
5. Currie A, Brigic A, Blencowe NS, Potter S, Faiz OD, Kennedy RH, et al. Systematic review of surgical innovation reporting in laparoendoscopic colonic polyp resection. Br J Surg. 2015;102(2):e108-16.
6. Muskens IS, Diederen SJH, Senders JT, Zamanipoor Najafabadi AH, van Furth WR, May AM, et al. Innovation in neurosurgery: less than IDEAL? A systematic review. Acta Neurochir (Wien). 2017;159(10):1957-66.
7. Ergina PL, Cook JA, Blazeby JM, Boutron I, Clavien PA, Reeves BC, et al. Challenges in evaluating surgical innovation. Lancet. 2009;374(9695):1097-104.
8. Khachane A, Philippou Y, Hirst A, McCulloch P. Appraising the uptake and use of the IDEAL Framework and Recommendations: A review of the literature. International journal of surgery (London, England). 2018;57:84-90.
9. Tradewell MB, Albersheim J, Dahm P. Use of the IDEAL framework in the urological literature: where are we in 2018? BJU Int. 2019;123(6):1078-85.
10. Birchley G, Huxtable R, Ives J, Blazeby J. Have We Made Progress in Identifying (Surgical) Innovation? The American Journal of Bioethics. 2019;19(6):25-7.
11. Meyerson D. Innovative surgery and the precautionary principle. J Med Philos. 2013;38(6):605-24.
12. Danjoux NM, Martin DK, Lehoux PN, Harnish JL, Shaul RZ, Bernstein M, et al. Adoption of an innovation to repair aortic aneurysms at a Canadian hospital: a qualitative case study and evaluation. BMC Health Serv Res. 2007;7:182.
13. Lee CB. Surgical innovation and safety: femoroacetabular impingement and the IDEAL collaborative framework. J Hip Preserv Surg. 2016;3(2):89-96.
14. McKneally MF, Daar AS. Introducing new technologies: protecting subjects of surgical innovation and research. World journal of surgery. 2003;27(8):930-4; discussion 4-5.
15. Poulin P, Austen L, Kortbeek JB, Lafreniere R. New technologies and surgical innovation: five years of a local health technology assessment program in a surgical department. Surg Innov. 2012;19(2):187-99.

For peer review only - http://bmjopen.bmj.com/site/about/guidelines.xhtml

16. Ives J, Birchley G, Huxtable R, Blazeby J. Hiding behind 'innovation': the case for regulated risk assessment in surgery. Br J Surg. 2022.
17. Biffl WL, Spain DA, Reitsma AM, Minter RM, Upperman J, Wilson M, et al. Responsible development and application of surgical innovations: a position statement of the Society of University Surgeons. J Am Coll Surg. 2008;206(6):1204-9.
18. Jain M, Duh QY, Hirose R, Sosa JA, Suh I. A model for the institutional adoption of innovative surgical techniques. Surgery. 2020;168(2):238-43.
19. Birchley G, Ives J, Huxtable R, Blazeby J. Conceptualising Surgical Innovation: An Eliminativist Proposal. Health Care Anal. 2020;28(1):73-97.
20. Rogers WA, Hutchison K, McNair A. Ethical Issues Across the IDEAL Stages of Surgical Innovation. 2019;269(2):229-33.
21. Hutchison K, Rogers W, Eyers A, Lotz M. Getting Clearer About Surgical Innovation A New Definition and a New Tool to Support Responsible Practice. Annals of Surgery. 2015;262(6):949-54.
22. Zahra J, Paramasivan S, Blencowe NS, Cousins S, Avery K, Mathews J, et al. Discussing surgical innovation with patients: a qualitative study of surgeons' and governance representatives' views. BMJ Open. 2020;10(11):e035251.
23. Mastroianni AC. Liability, regulation and policy in surgical innovation: the cutting edge of research and therapy. Health Matrix Clevel. 2006;16(2):351-442.
24. Rogers WA, Lotz M, Hutchison K, Pourmoslemi A, Eyers A. Identifying surgical innovation: a qualitative study of surgeons' views. Ann Surg. 2014;259(2):273-8.
25. Zaki MM, Cote DJ, Muskens IS, Smith TR, Broekman ML. Defining Innovation in Neurosurgery: Results from an International Survey. World neurosurgery. 2018;114:e1038-e48.
26. Pennell CP, Hirst AD, Campbell WB, Sood A, Agha RA, Barkun JS, et al. Practical guide to the Idea, Development and Exploration stages of the IDEAL Framework and Recommendations. Br J Surg. 2016;103(5):607-15.
27. Macefield RC, Wilson N, Hoffmann C, Blazeby JM, McNair AGK, Avery KNL, et al. Outcome selection, measurement and reporting for new surgical procedures and devices: a systematic review of IDEAL/IDEAL-D studies to inform development of a core outcome set. BJS Open. 2020.
28. Pennell CP, Hirst A, Sedrakyan A, McCulloch PG. Adapting the IDEAL Framework and Recommendations for medical device evaluation: A modified Delphi survey. International journal of surgery (London, England). 2016;28:141-8.
29. McCulloch P, Cook JA, Altman DG, Heneghan C, Diener MK. IDEAL framework for surgical innovation 1: the idea and development stages. Bmj. 2013;346:f3012.
30. Hirst A, Agha RA, Rosin D, McCulloch P. How can we improve surgical research and innovation?: the IDEAL framework for action. International journal of surgery (London, England). 2013;11(10):1038-42.
31. Ergina PL, Barkun JS, McCulloch P, Cook JA, Altman DG. IDEAL framework for surgical innovation 2: observational studies in the exploration and assessment stages. Bmj. 2013;346:f3011.
32. Cook JA, McCulloch P, Blazeby JM, Beard DJ, Marinac-Dabic D, Sedrakyan A. IDEAL framework for surgical innovation 3: randomised controlled trials in the assessment stage and evaluations in the long term study stage. Bmj. 2013;346:f2820.
33. Barkun JS, Aronson JK, Feldman LS, Maddern GJ, Strasberg SM, Altman DG, et al. Evaluation and stages of surgical innovations. Lancet. 2009;374(9695):1089-96.
34. McCulloch P, Altman DG, Campbell WB, Flum DR, Glasziou P, Marshall JC, et al. No surgical innovation without evaluation: the IDEAL recommendations. Lancet. 2009;374(9695):1105-12.
35. Team TE. EndNote. Philidelphia, PA: Clarivate; 2013.
36. Cousins S, Blencowe NS, Blazeby JM. What is an invasive procedure? A definition to inform study design, evidence synthesis and research tracking. BMJ Open. 2019;9(7):e028576.
37. Harris PA, Taylor R, Minor BL, Elliott V, Fernandez M, O'Neal L, et al. The REDCap consortium: Building an international community of software platform partners. J Biomed Inform. 2019;95:103208.

For peer review only - http://bmjopen.bmj.com/site/about/guidelines.xhtml

38. Harris PA, Taylor R, Thielke R, Payne J, Gonzalez N, Conde JG. Research electronic data capture (REDCap)--a metadata-driven methodology and workflow process for providing translational research informatics support. J Biomed Inform. 2009;42(2):377-81.
39. National Institute for Health and Care Excellence. The NICE Glossary [Available from: https://www.nice.org.uk/Glossary.
40. Gale NK, Heath G, Cameron E, Rashid S, Redwood S. Using the framework method for the analysis of qualitative data in multi-disciplinary health research. BMC Med Res Methodol. 2013;13:117.
41. Solari V, Jawaid W, Jesudason EC. Enhancing safety of laparoscopic vascular control for neonatal sacrococcygeal teratoma. J Pediatr Surg. 2011;46(5):E5-E7.
42. Diepstraten SCE, Verkooijen HM, van Diest PJ, Veldhuis WB, Fernandez-Gallardo AM, Duvivier KM, et al. Radiofrequency-assisted intact specimen biopsy of breast tumors: critical evaluation according to the IDEAL recommendations. Cancer Imaging. 2011;11(1):247-52.
43. Minervini A, Vanacore D, Vittori G, Milanesi M, Tuccio A, Siena G, et al. Florence robotic intracorporeal neobladder (FloRIN): a new reconfiguration strategy developed following the IDEAL guidelines. Bju International. 2018;121(2):313-7.
44. Greco F, Pini G, Alba S, Altieri VM, Verze P, Mirone V. Minilaparoendoscopic Single-site Pyeloplasty: The Best Compromise Between Surgeon's Ergonomy and Patient's Cosmesis (IDEAL Phase 2a). European Urology Focus. 2016;2(3):319-26.
45. Rischmann P, Gelet A, Riche B, Villers A, Pasticier G, Bondil P, et al. Focal High Intensity Focused Ultrasound of Unilateral Localized Prostate Cancer: A Prospective Multicentric Hemiablation Study of 111 Patients. Eur Urol. 2017;71(2):267-73.
46. Breda A, Territo A, Guttilla A, Sanguedolce F, Manfredi M, Quaresima L, et al. Correlation Between Confocal Laser Endomicroscopy (Cellvizio(®)) and Histological Grading of Upper Tract Urothelial Carcinoma: A Step Forward for a Better Selection of Patients Suitable for Conservative Management. Eur Urol Focus. 2018;4(6):954-9.
47. Bus MTJ, de Bruin DM, Faber DJ, Kamphuis GM, Zondervan PJ, Laguna-Pes MP, et al. Optical Coherence Tomography as a Tool for In Vivo Staging and Grading of Upper Urinary Tract Urothelial Carcinoma: A Study of Diagnostic Accuracy. J Urology. 2016;196(6):1749-55.
48. Graff P, Portalez D, Lusque A, Brun T, Aziza R, Khalifa J, et al. IDEAL 2a Phase II Study of Ultrafocal Brachytherapy for Low- and Intermediate-risk Prostate Cancer. Int J Radiat Oncol. 2018;102(4):903-11.
49. Liem EIML, Freund JE, Baard J, de Bruin DM, Pes MPL, Savci-Heijink CD, et al. Confocal Laser Endomicroscopy for the Diagnosis of Urothelial Carcinoma in the Bladder and the Upper Urinary Tract: Protocols for Two Prospective Explorative Studies. Jmir Res Protoc. 2018;7(2).
50. Swaan A, Mannaerts CK, Scheltema MJV, Nieuwenhuijzen JA, Savci-Heijink CD, de la Rosette JJMCH, et al. Confocal Laser Endomicroscopy and Optical Coherence Tomography for the Diagnosis of Prostate Cancer: A Needle-Based, In Vivo Feasibility Study Protocol (IDEAL Phase 2A). Jmir Res Protoc. 2018;7(5).
51. Blazeby JM, Blencowe NS, Titcomb DR, Metcalfe C, Hollowood AD, Barham CP. Demonstration of the IDEAL recommendations for evaluating and reporting surgical innovation in minimally invasive oesophagectomy. Brit J Surg. 2011;98(4):544-51.
52. Dumont F, Duchalais E, Aumont A, Thibaudeau E. Cytoreductive surgery plus hyperthermic intraperitoneal chemotherapy by laparoscopy via a single-port approach for low-grade peritoneal malignancy. Surgical Endoscopy and Other Interventional Techniques. 2020;34(6):2789-95.
53. Goh BKP, Prieto M, Syn N, Koh YX, Lim KI. Critical appraisal of the learning curve of minimally invasive hepatectomy: experience with the first 200 cases of a Southeast Asian early adopter. Anz Journal of Surgery. 2020;90(6):1092-8.
54. Inoue Y, Saiura A, Sato T, Ishizawa T, Arita J, Takahashi Y, et al. Laparoscopic pancreatoduodenectomy combined with a novel self-assessment system and feedback discussion: a phase 1 surgical trial following the IDEAL guidelines. Langenbeck Arch Surg. 2016;401(8):1123-30.

For peer review only - http://bmjopen.bmj.com/site/about/guidelines.xhtml

55. Stenstra MHBC, van Workum F, van den Wildenberg FJH, Polat F, Rosman C. Evolution of the surgical technique of minimally invasive Ivor-Lewis esophagectomy: description according to the IDEAL framework. Diseases of the Esophagus. 2019;32(3).
56. Gerullis H, Ecke TH, Barski D, Bantel C, Weyland A, Uphoff J, et al. Retrospective analysis of a surgical innovation using the IDEAL framework: radical cystectomy with epidural anaesthesia. J Int Med Res. 2017;45(2):714-22.
57. Gerullis H, Barski D, Georgas E, Boros M, Ramon A, Ecke TH, et al. Protocol for a Randomized Phase II Trial for Mesh Optimization by Autologous Plasma Coating in Prolapse Repair: IDEAL Stage 3. Adv Ther. 2017;34(4):995-1006.
58. Gaboardi F, Pini G, Suardi N, Montorsi F, Passaretti G, Smelzo S. Robotic laparoendoscopic single-site radical prostatectomy (R-LESS-RP) with daVinci Single-Site (R) platform. Concept and evolution of the technique following an IDEAL phase 1. J Robot Surg. 2019;13(2):215-26.
59. Kaouk JH, Haber GP, Autorino R, Crouzet S, Ouzzane A, Flamand V, et al. A Novel Robotic System for Single-port Urologic Surgery: First Clinical Investigation. Eur Urol. 2014;66(6):1033-43.
60. del Val ID, Loureiro C, McCulloch P. The IDEAL prospective development study format for reporting surgical innovations. An illustrative case study of robotic oesophagectomy. International Journal of Surgery. 2015;19:104-11.
61. Barski D, Arndt C, Gerullis H, Yang J, Boros M, Otto T, et al. Transvaginal PVDF-mesh for cystocele repair: A cohort study. International Journal of Surgery. 2017;39:249-54.
62. Versteeg AL, van der Velden JM, Hes J, Eppinga W, Kasperts N, Verkooijen HM, et al. Stereotactic Radiotherapy Followed by Surgical Stabilization Within 24 h for Unstable Spinal Metastases; A Stage I/IIa Study According to the IDEAL Framework. Front Oncol. 2018;8.
63. Mannaerts CK, Wildeboer RR, Postema AW, Hagemann J, Budaus L, Tilki D, et al. Multiparametric ultrasound: evaluation of greyscale, shear wave elastography and contrast-enhanced ultrasound for prostate cancer detection and localization in correlation to radical prostatectomy specimens. Bmc Urol. 2018;18.
64. Barentsz MW, Verkooijen HM, Pijnappel RM, Fernandez MA, van Diest PJ, van der Pol CC, et al. Sentinel lymph node localization with contrast-enhanced ultrasound and an I-125 seed: An ideal prospective development study. International Journal of Surgery. 2015;14:1-6.
65. Chan JYK, Tsang RK, Holsinger FC, Tong MCF, Ng CWK, Chiu PWY, et al. Prospective clinical trial to evaluate safety and feasibility of using a single port flexible robotic system for transoral head and neck surgery. Oral Oncol. 2019;94:101-5.
66. Hallmann S, Petersein J, Ruttloff J, Ecke TH. Successful evacuation of large perirenal hematoma after extracorporeal shock wave lithotripsy (ESWL) - step 1 of the IDEAL recommendations of surgical innovation. Clin Case Rep. 2017;5(2):123-5.
67. Valerio M, Dickinson L, Ali A, Ramachandran N, Donaldson I, Freeman A, et al. A prospective development study investigating focal irreversible electroporation in men with localised prostate cancer: Nanoknife Electroporation Ablation Trial (NEAT). Contemporary clinical trials. 2014;39(1):57-65.
68. Gerullis H, Schwartmann K, Ecke TH, Georgas E, Herberg P, Winter A, et al. Transvesical Suprapubic Externalization of Ureteral Stents - Introduction of a Surgical Innovation at the Development Stage. Urol Int. 2017;99(1):69-76.
69. Currie AC, Blazeby JM, Suzuki N, Thomas-Gibson S, Reeves B, Morton D, et al. Evaluation of an early-stage innovation for full-thickness excision of benign colonic polyps using the IDEAL framework. Colorectal Disease. 2019;21(9):1004-16.
70. Sharma SK, Momose K, Sedrakyan A, Sonoda T, Sharaiha RZ. Endoscopic stabilization device evaluation using IDEAL framework: A quality improvement study. International Journal of Surgery. 2019;67:18-23.
71. Menon M, Abaza R, Sood A, Ahlawat R, Ghani KR, Jeong W, et al. Robotic Kidney Transplantation with Regional Hypothermia: Evolution of a Novel Procedure Utilizing the IDEAL Guidelines (IDEAL Phase 0 and 1). Eur Urol. 2014;65(5):1001-9.

For peer review only - http://bmjopen.bmj.com/site/about/guidelines.xhtml

72. Sood A, McCulloch P, Dahm P, Ahlawat R, Jeong WJ, Bhandari M, et al. Ontogeny of a surgical technique: Robotic kidney transplantation with regional hypothermia. International Journal of Surgery. 2016;25:158-61.
73. Banagala S, Jayarajah U, Almeida I, Samarasekera DN. Efficacy of irrigation tubes in the management of para rectal cavities associated with complex fistula-in-ano. Bmc Surgery. 2018;18.
74. van Kollenburg RAA, van Riel LAMJG, Bloemen PR, Oddens JR, de Reijke TM, Beerlage HP, et al. Transperineal Laser Ablation Treatment for Lower Urinary Tract Symptoms Due to Benign Prostatic Obstruction: Protocol for a Prospective In Vivo Pilot Study. Jmir Res Protoc. 2020;9(1).
75. Harvey KL, Mills N, White P, Holcombe C, Potter S, Steering P-BFS. The Pre-BRA (pre-pectoral Breast Reconstruction EvAluation) feasibility study: protocol for a mixed-methods IDEAL 2a/2b prospective cohort study to determine the safety and effectiveness of prepectoral implant-based breast reconstruction. Bmj Open. 2020;10(1).
76. Buijs M, van Lienden KP, Wagstaff PGK, Scheltema MJV, de Bruin DM, Zondervan PJ, et al. Irreversible Electroporation for the Ablation of Renal Cell Carcinoma: A Prospective, Human, In Vivo Study Protocol (IDEAL Phase 2b). Jmir Res Protoc. 2017;6(2).
77. Villers A, Puech P, Flamand V, Haber GP, Desai MM, Crouzet S, et al. Partial Prostatectomy for Anterior Cancer: Short-term Oncologic and Functional Outcomes. Eur Urol. 2017;72(3):333-42.
78. Barski D, Gerullis H, Ecke T, Varga G, Boros M, Pintelon I, et al. Repair of a vesico-vaginal fistula with amniotic membrane - Step 1 of the IDEAL recommendations of surgical innovation. Cent Eur J Urol. 2015;68(4):459-61.
79. Joukhadar R, Wockel A, Herr D, Paulus V, Radosa J, Hamza A, et al. Challenges of Longevity: Safety of Vaginal and Laparoscopic Urogynecological Procedures in Septuagenarians and Older Patients. Biomed Res Int. 2016;2016.
80. Heikens JT, Gooszen HG, Rovers MM, van Laarhoven CJHM. Stages and Evaluation of Surgical Innovation: A Clinical Example of the Ileo Neorectal Anastomosis After Ulcerative Colitis and Familial Adenomatous Polyposis. Surgical Innovation. 2013;20(5):459-65.
81. Saglam R, Muslumanoglu AY, Tokath Z, Caskurlu T, Sarica K, Tasci AI, et al. A New Robot for Flexible Ureteroscopy: Development and Early Clinical Results (IDEAL Stage 1-2b). Eur Urol. 2014;66(6):1092-100.
82. Lotz M. Surgical innovation as sui generis surgical research. Theoretical medicine and bioethics. 2013;34(6):447-59.
83. Hasenboehler EA, Choudhry IK, Newman JT, Smith WR, Ziran BH, Stahel PF. Bias towards publishing positive results in orthopedic and general surgery: a patient safety issue? Patient Saf Surg. 2007;1(1):4.
84. Ross JS, Mulvey GK, Hines EM, Nissen SE, Krumholz HM. Trial publication after registration in ClinicalTrials.Gov: a cross-sectional analysis. PLoS Med. 2009;6(9):e1000144.
85. Song F, Parekh S, Hooper L, Loke YK, Ryder J, Sutton AJ, et al. Dissemination and publication of research findings: an updated review of related biases. Health Technol Assess. 2010;14(8):iii, ix-xi, 1-193.
86. Schekman R. How journals like Nature, Cell and Science are damaging science: The Guardian; 2013 [Available from: https://www.theguardian.com/commentisfree/2013/dec/09/how-journals-nature-science-cell-damage-science.
87. Vigano L, Giuliani A, Calise F. The dilemma of surgical research between evidences and experience, impact factor and innovation. Updates Surg. 2019;71(1):3-5.
88. Cousins S, Richards HS, Zahra J, Robertson H, Mathews JA, Avery KNL, et al. Healthcare organization policy recommendations for the governance of surgical innovation: review of NHS policies. Br J Surg. 2022;109(10):1004-12.
89. Stefanidis D, Fanelli RD, Price R, Richardson W, Committee SG. SAGES guidelines for the introduction of new technology and techniques. Surg Endosc. 2014;28(8):2257-71.
90. Surgeons RCo. Surgical Innovation, New Techniques and Technologies. 2019.
91. Surgeons RCo. Learning from invited reviews. 2019.

For peer review only - http://bmjopen.bmj.com/site/about/guidelines.xhtml

92. Elliott D, Blencowe NS, Cousins S, Zahra J, Skilton A, Mathews J, et al. Using qualitative research methods to understand how surgical procedures and devices are introduced into NHS hospitals: the Lotus study protocol. BMJ Open. 2021;11(12):e049234.
93. Siemeiniuk RG, G. . What is GRADE? 2023 [Available from: https://bestpractice.bmj.com/info/toolkit/learn-ebm/what-is-grade/.
94. Emamaullee J, Bowdish M, Yan PY, Rodina V, Sher LS. Rapid Adaptation of a Surgical Research Unit to Conduct Clinical Trials During the COVID-19 Pandemic. J Surg Res. 2020;256:76-82.
95. Lupkin S. Coronavirus pandemic Brings Hundreds of U.S. Clinical trials to A Halt. : Heal News from NPR; 2020 [Available from: https://www.npr.org/sections/health-shots/2020/04/11/832210606/coronovirus-pandemic-brings-hundreds-of-u-s-clinical-trials-to-a-halt.
96. Thornton J. Clinical trials suspended in UK to prioritise covid-19 studies and free up staff. Bmj-Brit Med J. 2020;368.
97. Glasbey JC, Ademuyiwa A, Adisa A, AlAmeer E, Arnaud AP, Ayasra F, et al. Effect of COVID-19 pandemic lockdowns on planned cancer surgery for 15 tumour types in 61 countries: an international, prospective, cohort study. Lancet Oncology. 2021;22(11):1507-17.
98. Stoss C, Steffani M, Kohlhaw K, Rudroff C, Staib L, Hartmann D, et al. The COVID-19 pandemic: impact on surgical departments of non-university hospitals. Bmc Surgery. 2020;20(1).

For peer review only

For peer review only - http://bmjopen.bmj.com/site/about/guidelines.xhtml

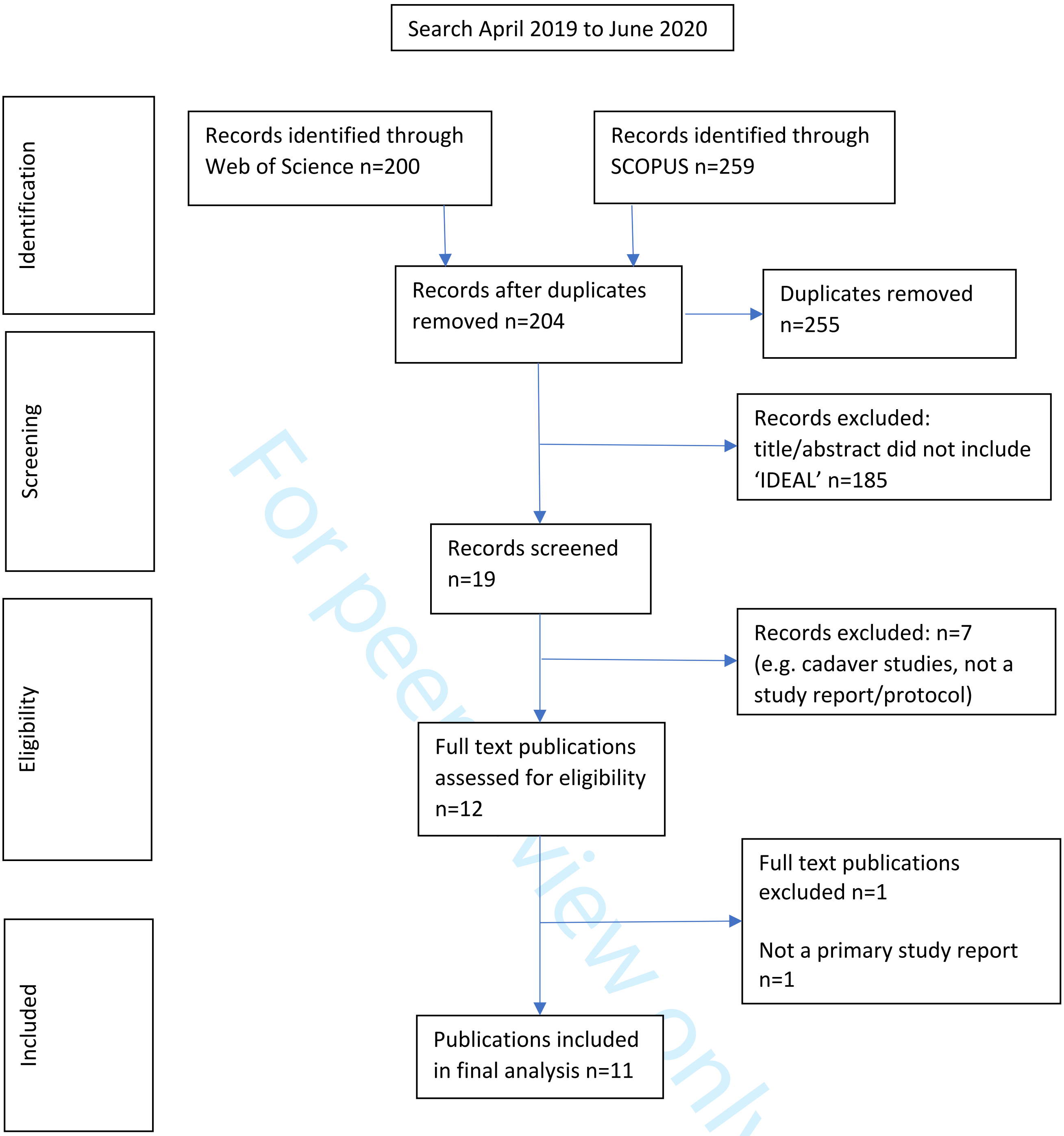
Search April 2019 to June 2020
Identification
Records identified through Web of Science n=200
Records identified through SCOPUS n=259
Records after duplicates removed n=204
Duplicates removed n=255
Screening
Records excluded: title/abstract did not include 'IDEAL' n=185
Records screened n=19
Eligibility
Records excluded: n=7 (e.g. cadaver studies, not a study report/protocol)
Full text publications assessed for eligibility n=12
Full text publications excluded n=1
Not a primary study report n=1
Included
Publications included in final analysis n=11

BMJ Open

Examining the application of the IDEAL Framework in the reporting and evaluation of innovative invasive procedures: secondary qualitative analysis of a systematic review.

Journal:	*BMJ Open*
Manuscript ID	bmjopen-2023-079654.R1
Article Type:	Original research
Date Submitted by the Author:	21-Mar-2024
Complete List of Authors:	Richards, Hollie; University of Bristol Medical School, Population Health Sciences<br>Cousins, Sian; University of Bristol<br>Scroggie, Darren; University of Bristol<br>Elliott, Daisy; University of Bristol<br>Macefield, Rhiannon; University of Bristol Medical School, Population Health Sciences<br>Alford, Natasha; University of Bristol Medical School<br>Shah, Maximilian; University of Bristol Medical School<br>Blencowe, Natalie; University of Bristol Medical School, Population Health Sciences<br>Blazeby, Jane; University of Bristol Medical School, Population Health Sciences
**Primary Subject Heading**:	Surgery
Secondary Subject Heading:	Ethics
Keywords:	SURGERY, QUALITATIVE RESEARCH, Systematic Review

SCHOLARONE™
Manuscripts

I, the Submitting Author has the right to grant and does grant on behalf of all authors of the Work (as defined in the below author licence), an exclusive licence and/or a non-exclusive licence for contributions from authors who are: i) UK Crown employees; ii) where BMJ has agreed a CC-BY licence shall apply, and/or iii) in accordance with the terms applicable for US Federal Government officers or employees acting as part of their official duties; on a worldwide, perpetual, irrevocable, royalty-free basis to BMJ Publishing Group Ltd ("BMJ") its licensees and where the relevant Journal is co-owned by BMJ to the co-owners of the Journal, to publish the Work in this journal and any other BMJ products and to exploit all rights, as set out in our licence.

The Submitting Author accepts and understands that any supply made under these terms is made by BMJ to the Submitting Author unless you are acting as an employee on behalf of your employer or a postgraduate student of an affiliated institution which is paying any applicable article publishing charge ("APC") for Open Access articles. Where the Submitting Author wishes to make the Work available on an Open Access basis (and intends to pay the relevant APC), the terms of reuse of such Open Access shall be governed by a Creative Commons licence – details of these licences and which Creative Commons licence will apply to this Work are set out in our licence referred to above.

Other than as permitted in any relevant BMJ Author's Self Archiving Policies, I confirm this Work has not been accepted for publication elsewhere, is not being considered for publication elsewhere and does not duplicate material already published. I confirm all authors consent to publication of this Work and authorise the granting of this licence.

For peer review only

Examining the application of the IDEAL Framework in the reporting and evaluation of innovative invasive procedures: secondary qualitative analysis of a systematic review.

[1]Richards, H.S*., [1]Cousins, S., [1]Scroggie, D., [1]Elliott, D., [1]Macefield, R., [1]Hudson, E., [1]Mutanga, I., [1]Shah, M., [1]Alford, N., [1]Blencowe, N.S & [1]Blazeby, J.M.

[1]National Institute for Health and Care Research Bristol Biomedical Research Centre, University Hospitals Bristol and Weston NHS Foundation Trust and University of Bristol, Bristol, United Kingdom.

*Corresponding author: Bristol Medical School: Population Health Sciences, University of Bristol, 39 Whatley Road, Bristol, BS8 2PS, United Kingdom. Email: hollie.richards@bristol.ac.uk,

ABSTRACT

Objectives

The development of new surgical procedures is fundamental to advancing patient care. The IDEAL framework describes study designs for stages of innovation. It can be difficult to apply due to challenges in defining and identifying innovative procedures. This study examined how the IDEAL framework is operationalised in real-world settings; specifically, types of innovations evaluated using the framework and how authors justify their choice of IDEAL study design.

Design

Secondary qualitative analysis of a systematic review.

Data Sources

Citation searches (Web of Science and Scopus) identified studies following the IDEAL framework and citing any of the ten key IDEAL/IDEAL_D papers.

Eligibility Criteria

Studies of invasive procedures/devices of any design citing any of the ten key IDEAL/IDEAL_D papers.

Data extraction and synthesis

For peer review only - http://bmjopen.bmj.com/site/about/guidelines.xhtml

All relevant text was extracted. Three frameworks were developed: i) type of innovation under evaluation; ii) terminology used to describe stage of innovation; and iii) reported rationale for IDEAL stage.

Results

Forty-eight articles were included. 19/48 described entirely new procedures, including those used for the first time in a different clinical context (n=15/48), reported as IDEAL stage 2a (n=8, 53%). Terminology describing stage of innovation was varied, inconsistent and ambiguous, and were not defined. Authors justified their choice of IDEAL study design based on limitations in published evidence (n=36) and unknown feasibility and safety (n=32) outcomes.

Conclusion

Identifying stage of innovation is crucial to inform appropriate study design and governance decisions. Authors' rationale for choice of IDEAL stage related to the existing evidence base or lack of sufficient outcome data for procedures. Stage of innovation was poorly defined with inconsistent descriptions. Further work is needed to develop methods to identify innovation to inform practical application of the IDEAL framework. Defining the concept of innovation in terms of uncertainty, risk and degree of evidence may help to inform decision making.

Strengths and limitations of this study

- This work provides an in-depth review of how the IDEAL Framework has been applied and reported by examining how authors self-report stage of innovation in relation to IDEAL and use this to inform study design decisions.
- There are discrepancies in how the IDEAL framework is operationalised in real-world settings, demonstrating the need for improved methods for identifying innovation in surgery.

For peer review only - http://bmjopen.bmj.com/site/about/guidelines.xhtml

For peer review only

- Limitations include the omission of potentially relevant papers where authors did not explicitly reference their use of the IDEAL Framework and that database searches took place in 2020.

INTRODUCTION

Innovations in healthcare requires evidence of safety and effectiveness before widespread implementation. Although pharmaceutical development occurs through a clearly delineated pathway, surgical innovation often happens haphazardly without incremental building of evidence through evaluation within formal research studies (1, 2). The IDEAL (Idea, Developments, Exploration, Assessment and Long-term follow-up) framework was developed to facilitate the evaluation of new surgical procedures and devices (IDEAL-D)(3, 4). It provides study design and governance recommendations for new procedures and devices at different stages of development. Stage 0 deals with pre-clinical studies, prior to stage 1 first in human studies. Stage 1 is followed by stage 2a developmental studies, which focus on technical details and ongoing modifications of the intervention, moving to stage 2b exploratory studies where the intervention and patient group are defined in multiple centres prior to proceeding to stage 3 comparative studies. These are typically randomised controlled trials (RCTs) comparing the now stabilised intervention against standard treatment. Stage 4 consists of long-term monitoring in registries (3, 4). Although IDEAL is an important contribution to the field, it has not been widely applied (1, 5, 6) and is not always used appropriately (7-9). Challenges to the use of the IDEAL recommendations include that this relies on the user determining the stage of innovation of a procedure *a priori*, usually by examining the previous literature, and that ‘innovation’ in itself is not well defined within the IDEAL Framework (10)

Commonly, definitions of surgical innovation are intrinsically linked to concepts of ‘newness’, including the extent to which a procedure differs from standard practice (11) or is being modified (11-15). It can also be defined as something with uncertain levels of risk (16). Requirements for additional training

For peer review only - http://bmjopen.bmj.com/site/about/guidelines.xhtml

(13, 15) and the degree of evidence of safety and efficacy (17, 18) have been used to determine how innovative an intervention is. However, some of these definitions are difficult to apply in practice (19). For example, it is often unclear what degree of modification constitutes innovation (10, 20). Guidance has been developed to help surgeons identify innovative procedures (21). The Macquarie Tool bases the degree of innovation on surgeons' previous experiences and how much the procedure varies from routine practice. This places the responsibility for identifying innovation on the surgeon (19), and as such may be open to individual interpretations (19, 22) and biases (23-25). IDEAL suggests that examination of published studies may help determine stage of innovation (26). Although this is useful for stage 1 proof-of-concept studies, where there is very limited or no published evidence about the innovation, the guidance acknowledges that the literature may be of limited use when interventions are at a later stage of innovation. Distinguishing IDEAL stage 2a from 2b may be problematic as centres achieve stability in a procedure at different times (26). Given these challenges, examination of how stage of innovation is determined and used by surgeons and is needed.

The aim of this study is to examine how innovative surgical procedures are conceptualised, evaluated and reported using the IDEAL Framework and to understand authors' rationale for choice of IDEAL study design. This will inform work to develop practical methods to facilitate determination of stage of innovation, to ensure appropriate study design and governance (4, 20).

METHODS

A secondary qualitative analysis of papers selected for a previous systematics review(27) was undertaken. Published papers self-reported as IDEAL stage 1 to 4 studies, reporting evaluations of innovative surgical procedures, were examined using a qualitative framework approach(28). (See Supplementary File 1 for PRISMA and ENTREQ checklists).

Patient and Public Involvement

For peer review only - http://bmjopen.bmj.com/site/about/guidelines.xhtml

Patients and members of the public were not directly involved in this secondary analysis of a systematic review. However, this work took place within the wider context of the Bristol Biomedical Research Centre Surgical and Orthopaedic Innovation Theme, which has engaged extensively with PPIE activities. This includes an active PPIE group involved in the co-production of projects and public engagement events about the definitions of new procedures and how they are introduced into clinical practice.

Searches

Articles identified in a previous systematic review(27) were retrieved. In this review, electronic citation searches of the Web of Science and Scopus databases were undertaken to identify all papers citing any of the ten key IDEAL papers (3, 7, 26, 29-35) published between January 2009 and April 2019 (Supplementary File 2). Records were further filtered to select only those with 'IDEAL' or 'IDEAL-D' in the title or abstract. Searches were updated in June 2020 using the same databases and search criteria to identify further studies. Articles were imported into reference management software (36) and duplicates were removed. No further searches were undertaken after 2020 because the current review focussed on authors' conceptualisation and description of innovation, and justification of their choice of study design. Such factors are unlikely to change across the time since the searches were undertaken.

Eligibility criteria and definitions

Eligibility criteria for inclusion in the previous review and updated search were studies of invasive procedures and devices of any design. Secondary studies and those not involving living human participants, were excluded, along with editorials, commentaries, conference proceedings and letters (27). Further exclusion criteria for the current review were studies where the intervention under evaluation was not the invasive procedure itself (e.g. non-invasive co-interventions). Invasive procedures were defined as interventions where access to the body was gained by incision, natural orifices or endoscopic techniques, or percutaneous puncture where instrumentation is used in

For peer review only - http://bmjopen.bmj.com/site/about/guidelines.xhtml

For peer review only

addition to the puncture needle (37). This could include for example a diagnostic endoscopy procedure that does not involve an incision. Invasive devices were defined as any instrument, apparatus or material that is used within or implanted into the body.

Screening

All studies included in the previous review and update were screened to ensure they met the updated eligibility criteria. Titles and abstracts were reviewed initially by one reviewer (HSR) and ineligible studies excluded. Full texts of all remaining articles were then screened. At each stage, 20% of articles were screened by a second reviewer (SC). Disagreements were resolved by discussion amongst the wider study team.

Data extraction and analysis

Study characteristics

Study characteristics were extracted using a standardised REDCap electronic data capture form (38, 39). Author-reported IDEAL stage, year of publication, country, the invasive procedure or device under evaluation, number of centres and surgeons, and sample size are reported in the previous systematic review (27). Additional details about reported oversight (e.g. research governance), study design, and surgical specialty were extracted by one reviewer (HSR). Study designs, determined by NICE definitions (40) were extracted from all papers by two independent reviewers (HSR, SC). To ensure clinical accuracy, data relating to surgical specialties was double-extracted from 20% of papers by a second independent surgeon reviewer (DS). Disagreements were resolved by discussion. Characteristics were reported using descriptive statistics, where appropriate.

Type of surgical innovation under evaluation and rationale for study design

Papers were analysed using a thematic framework approach (28). This structured approach involves coding of data to identify themes to form a framework, which was applied in an iterative and reflexive

For peer review only - http://bmjopen.bmj.com/site/about/guidelines.xhtml

manner (28). Full publications were imported into NVivo (version 13) and analysed line by line. Text was coded (whereby text of interest is identified, and descriptive labels attached) using a combination of deductive and inductive approaches, allowing for any unexpected themes to develop. This ensured that analysis was data driven. Three frameworks were developed according to study aims. The first (1) related to the type of innovation under evaluation. All text describing the surgical innovation under evaluation was extracted and coded inductively. For each study, the innovative component under evaluation was identified, for example if the procedure was described as being used for the first time or if changes to a procedure were reported. Where procedural changes were reported, a typology that facilitates the deconstruction of invasive procedures into their constituent parts was utilised to identify how many, and which, steps of the procedure were changed (37). Where codes shared commonalities, these were grouped together into themes and sub-themes to form a framework. Where the innovation under evaluation involved a device or robot, these formed a separate theme. The second (2) framework related to the terminology used to describe the stage of innovation of the procedure. This was analysed according to pre-agreed themes: 'novel/new' – the first or early use of the procedure/device; 'modified' – planned or unplanned changes to the procedure/device or patient group; 'stabilised' – the finalisation of iterative changes or lack of subsequent changes/modifications; and 'adopted' – the wider use of the procedure/device in clinical practice. If the terminology describing stage of innovation could not be coded to these themes, additional themes were created. The third (3) framework related to the reported rationale for study design. All text describing the author's reported rationale for choice of study design was extracted and coded inductively. A narrative summary was produced to illustrate findings with reference to study characteristics.

Framework development

Three multi-disciplinary (qualitative research (HSR), health services research (SC) and surgical (DS)) members of the team independently coded an initial five articles and discussed how the text had been interpreted. This allowed similarities and differences to be incorporated into an initial set of

For peer review only - http://bmjopen.bmj.com/site/about/guidelines.xhtml

codes and themes with clear definitions (28). This process was repeated iteratively until agreement was reached on the definitions of codes and themes/sub-themes. A senior member of the team (JMB) reviewed coded text and the iterative development of frameworks. Each framework was then applied to all articles by one researcher (HSR). The research team (HSR, SC, DC and JMB) met regularly throughout this process to discuss analysis, ensuring agreement was reached regarding the appropriate interpretation of the text.

RESULTS

From the 74 papers retrieved from the previous review (27) and update (Supplementary File 3), 48 were included.

For peer review only

For peer review only - http://bmjopen.bmj.com/site/about/guidelines.xhtml

Study characteristics		Total, n=48 (%)	Author-reported IDEAL Stage					
			1 (n=11)	2a (n=18)	2b (n=6)	3 (n=2)	Multiple (n=9)	Not stated (n=2)
Study Design	Case series	36 (75)	7	15	6	-	7	1
	Case study	6 (13)	4	1	-	-	-	1
	Cohort study	4 (8)	-	2	-	1	1	-
	Randomised trial	1 (2)	-	-	-	1	-	-
	Multiple study designs	1 (2)	-	-	-	-	1	-
Surgical Specialty	Urology	25 (52)	6	10	4	1	4	-
	Gastrointestinal surgery	7 (15)	1	2	1	1	2	-
	Colorectal surgery	5 (10)	1	2	-	-	2	-
	Gynaecology and obstetrics	2 (4)	1	-	1	-	-	-
	Breast surgery	3 (6)	-	2	-	-	-	1
	Otolaryngology	2 (4)	-	2	-	-	-	-
	Maxillofacial and dental	2 (4)	2	-	-	-	-	-
	Orthopaedics	1 (2)	-	-	-	-	1	-
	Paediatric	1 (2)	-	-	-	-	-	1
Country	European Economic Area (Non-UK)	30 (63)	6	11	6	1	5	1
	Other[1]	9 (19)	2	3	-	1	3	-
	UK	8 (16)	2	4	-	-	1	1
	North America	1 (2)	1	-	-	-	-	-
Publication year	2011 - 2015	12 (25)	3	4	-	-	3	-
	2016 - 2020	36 (75)	8	14	6	2	6	-
Single or multi-centre study	Single centre	41 (85)	11	15	5	1	7	2
	Multi centre	5 (10)	-	2	1	1	1	-
	Not reported	2 (4)	-	1	-	-	1	-
Number of surgeons performing the intervention	1 to 2	22 (46)	6	10	-	2	3	1
	3 to 7	5 (10)	-	2	2	-	1	-
	Not reported/Unclear	21 (44)	5	6	4	-	5	1
Number of participants	1-10	10 (21)	7	2	-	-	-	1
	11-50	26 (54)	4	14	2	-	5	1
	51-100	5 (10)	-	1	1	-	3	-
	101-400	5 (10)	-	1	2	1	1	-
	401-510	2 (4)	-	-	1	1	-	-

Table 1. Characteristics of included studies.

For peer review only

For peer review only - http://bmjopen.bmj.com/site/about/guidelines.xhtml

Study characteristics

Characteristics of included publications are detailed in Table 1. The majority of studies were case series (n=36, 75%) or case studies (n=6, 13%). Most studies were reported as IDEAL stage 2a (n=18) although two did not explicitly state a stage: one stated it was stage 1 or 2a (41) and the other self-reported as an early-stage assessment of technical problems during the learning curve “in line with the IDEAL recommendations” (42). Nine papers reported the progression of an intervention through multiple IDEAL stages.

Table 2 details reported governance arrangements. Eighteen (36%) reported research governance, including approval from Institutional Review Boards or Research Ethics Committees overseen by the U.S. Food and Drug Administration (FDA) or the U.K NHS Health Research Authority (HRA). Most of these were reported as stage 2a (n=7). Thirteen (27%) papers reported obtaining local/institutional approval, using a variety of terms such as “local ethical committee” and “institutional medical ethical committee” to describe them. Five (10%) papers, including three reported as IDEAL stage 1, stated that ethical approval was not required or obtained. The remaining twelve (25%) papers, including IDEAL stage 1 (n=3) and stage 2a (n=5), did not report any governance approvals.

For peer review only

Reported governance approvals		Total (%) N=48	Author-reported IDEAL Stage					
			1 (n=11)	2a (n=18)	2b (n=6)	3 (n=2)	Multiple (n=9)	Not stated (n=2)
Formal research governance	Institutional Review Board (IRB)	14	4	5	2	1	2	-
	“REC ethical approval”	1	-	1	-	-	-	-
	“REC ethical approval (& NPC)”	1	-	1	-	-	-	-
	“Regional ethics commissions”	1	-	-	-	1	-	-
	“National ethics committee”	1	-	-	1	-	-	-
Local or institutional approvals	“Local ethical committee”	2	-	1	1	-	-	-
	“Local ethics board”	1	-	-	-	-	1	-
	“Local ethics review committee”	1	-	1	-	-	-	-
	“Local medical ethical committee”	2	-	1	1	-	-	-
	“Local research ethics committee”	3	1	1	-	-	1	-
	“Institution ethical committee”	1	-	1	-	-	-	-
	“Institutional health sciences research ethics board”	1	-	-	-	-	1	-
	“Institutional medical ethical committee”	1	-	-	-	-	1	-
	“Ethical commission of medical school”	1	-	-	-	-	1	-
No ethical approval obtained	"Current study: no ethical approval."	1	-	1	-	-	-	-
	“Audit - ethics not required”	1	-	-	-	-	1	-
	“Ethical approval not required”	1	1	-	-	-	-	-
	“No further ethics approval required.”	1	1	-	-	-	-	-
	“Not needed due to retrospective design”	1	1	-	-	-	-	-
Not reported	N/A	12	3	5	1	-	1	2

Table 2. Governance approvals reported by 48 included studies.

For peer review only - http://bmjopen.bmj.com/site/about/guidelines.xhtml

(1) Type of innovation under evaluation

The types of innovations under evaluation were categorised into five themes related to 'newness' (Table 3); i) an entire new procedure (this included if a procedure had two or more new steps; n=19); ii) one step of the procedure is new; iii) a new device including robotic approach; iv) changes to the sequence of procedure components; and v) changes to the combination of procedure components.

i) An entire new procedure

Nineteen (39%) papers reported an entire new procedure. Most (n=15) referred to procedures being newly used in a different clinical context, for example, combining existing minimally invasive techniques into a new type of reconstruction or anastomosis in bowel (43) and urological (44) surgery. Of these, 8 were classified as IDEAL 1a. Three (6%) other papers described procedures being applied to a different clinical indication, for example high intensity focused ultrasound applied to focal therapy for prostate tumours (45).

ii) One step of the procedure is new

Nineteen other papers described procedures with one step changed. Most (n=8) were stage 2a. Ten involved one step of a procedure being applied to different clinical indication, for example an existing invasive imaging technique currently used for one type of cancer being used for a different type of cancer (46-50). Six described changes to access to a body cavity by using minimally invasive techniques instead of open surgery (41, 51-55). One paper described the application of one step of an invasive procedure to a different patient group (56).

iii) A new device including robotic approach

Eight (17%) papers described the evaluation of an invasive device/robot. Of these, four described changes to one component of an invasive device (n=1) or robot (n=3). These were a modification to the coating of a vaginal implant device (57), a new access port for use with a robot devices (58, 59) and changing part of the procedure from open to robotic (60). These were reported as IDEAL stage 1 (n=2), stage 2a (n=1) or stage 3 (n=1) respectively. Three papers described the development of an entirely new invasive device (n=1) or robot (n=2). (61).

For peer review only - http://bmjopen.bmj.com/site/about/guidelines.xhtml

Main theme	Subthemes (if applicable)	Total (n=48)	Author-reported IDEAL stage and number of papers with text coded to theme					
			1 (n=11)	2a (n=18)	2b (n=6)	3 (n=2)	Multiple Stages (n=9)	Not stated (n=2)
An entire new procedure	Procedure used for the first time in that clinical context	15 (31%)	3	8	-	-	3	1
	Procedure applied to a different clinical indication	3 (6%)	1	-	1	-	1	-
	Procedure previously used in different patient group	1 (2%)	-	-	1	-	-	-
One step of the procedure is new	Step of procedure applied to a different clinical indication	10 (21%)	-	7	2	-	1	-
	Change to access	6 (12%)	1	-	1	1	2	1
	Change to reconstruction	2 (4%)	1	1	-	-	-	-
	Step of a procedure previously used in different patient group	1 (2%)	1	-	-	-	-	-
A new device including robotic approach	Change to one component of a device/robotic instruments	4 (8%)	2	1	-	1	-	-
	Development of an entire device/robotic instruments for one component of procedure	3 (6%)	1	1	-	-	1	-
	Existing device applied to a different clinical indication	1 (2%)	1	-	-	-	-	-
Novel changes to the sequence of procedure components	N/A	1 (2%)	-	-	-	-	1	-
Changes to the combination of procedure components	N/A	1 (2%)	-	-	1	-	-	-

Table 3. Main themes and sub-themes describing the type of innovation, by author-reported IDEAL stage and in total.

For peer review only

For peer review only - http://bmjopen.bmj.com/site/about/guidelines.xhtml

iv) Changes to the sequence of procedure components

One paper reporting a sequential IDEAL stages 2a and 2b study evaluated changes to the timing of radiotherapy for unstable spinal metastases (62).

v) Changes to the combination of procedure components

One IDEAL stage 2b study described an evaluation of combined versus individual invasive diagnostic tests (63).

(2) Terminology used to describe stage of innovation

In addition to the four *a priori* themes, the theme of abandoned procedures was identified during data analyses. The final five themes, (i) new/novel; ii) modified; iii) stabilised; iv) adopted; and v) abandoned procedures, are summarised below.

i) New/novel

There was a lack of consistency across papers reporting all IDEAL stages in terms describing the ‘newness’ of the procedure. Commonly terms included ‘innovation’ (n=18), ‘new’ (n=14), ‘novel’ (n=13), ‘introduction’ (n=4) and ‘first-in-human’ (n=4). ‘Innovation’ was used in papers reporting all IDEAL stages, whereas ‘introduction’ was predominantly used in stage 1 papers (n=3). None of the papers defined these terms. ‘First-in-human’ was used in one IDEAL stage 1 study, one stage 3 and two multistage studies, but in these latter cases it described the earlier development of the innovation under evaluation. Twenty studies used terminology and phrases related to the first report of the innovation (e.g. “this is the first study” (64), “first clinical trial” (65) and “the first case” (66)). The majority of these were stage 1 (n=7, 16%) and stage 2a (n=8, 17%). Five papers described the procedure as “emerging” (41) or “promising” (67). These were reported as IDEAL stage 2a (n=3), 2b (n=1) or not stated (n=1).

ii) Modified

For peer review only - http://bmjopen.bmj.com/site/about/guidelines.xhtml

For peer review only

Twenty-three papers, mostly early stage 1-2b, used a variety of terms to describe modifications. Although the terms ‘modified’ or ‘modification’ were used in eleven (23%) papers, most (n=18, 38%) described modifications in terms of the evolution and ongoing development of the procedure. This included the “evolution of the new method” (68), describing the procedure as “still under development” (69). Ten (21%) papers described changes based on their experience, e.g. “from case 35 onward” (61), “based on the results of patient 1” (54) and “thereafter we noticed” (58). These tended to appear in stage 2a (n=4) and stage 1 (n=3) studies. Some papers used more precise terms, such as ‘refinements’ (n=7, 16%), ‘changes’ (n=5, 11%) and ‘adaptions/adjustments/alterations’ (n=4, 8%). Others were vaguer. Terms included “hybrid solutions” (58) and “continued…optimization” (70). Additional undefined terms for modifications included “experimented” (71) and “improvised” (72), and descriptions of steps of the procedure being “replaced” (55) or “tried [and] tested” (72).

iii) Stabilised

Eight papers described the procedure using terms synonymous with stabilised (n=2 stage 1; n=3 stage 2a; n=1 stage 3; n=2 multiple stages). Terms and phrases included having “achieved stability” (70), “appear[ing] stable” (69) or “standardized” (58). Some authors were slightly more specific and described when procedures were no longer undergoing modifications, in two cases explicitly stating that this was an indication of stabilisation as “all surgeons began learning” the procedure (51) or it “had entered IDEAL stage 3” (55).

iv) Adopted

Four papers described the procedure being adopted into clinical practice (n=2 stage 2a; n=1 stage 3; n=1 multiple stages). One said the procedure was “an established technique in our unit” (73) and another said the procedure “may become a standard” (46) upon wider adoption.

v) Abandoned

For peer review only - http://bmjopen.bmj.com/site/about/guidelines.xhtml
For peer review only

Three papers stated that the procedure had been abandoned (n=1 stage 2a; n=2 multiple stages), including descriptions of the procedure as “not a viable alternative” (64) or as being halted due to “loss of confidence” (51).

(3) Reported rationale for study design

Three main themes were identified (Table 4): i) the limitations and quality of previously published evidence; ii) unknown feasibility and safety outcomes; and iii) previous use in clinical practice.

(i) The limitations and quality of published evidence

Thirty-six papers described the limitations and quality of previously published evidence as a rationale for the current study design. Of these, 31 discussed the limited number of published studies evaluating the procedure. These authors highlighted the small numbers of participants and surgeons in previous studies (e.g. “treated 18 patients” (74) and “a few expert surgeons” (75)). Fifteen other papers highlighted limitations in the types of outcomes collected in previous studies and three (74, 76, 77) (6%) (n=2 stage 2a; n=1 stage 2b) highlighted a lack of long-term outcomes as rationale for the current study. Three papers (44, 55, 78) (stage 1, stage 2a and stage 2b) stated that the procedure was not standardised. Other papers (n=13) focused on previous study designs and stated that there were few prospective studies, and that most evidence was from case series, case control studies, proof-of-concept or animal model studies. Of these, 10 were reported as stage 2a (n=8) or stage 1 (n=2) studies. In addition, four papers (48, 69, 75, 79) highlighted that findings from previous studies were inconsistent, inconclusive or conflicting, suggesting further evaluation was required. Another two stage 1 papers (54, 56) commented on potential biases in previous studies, specifically gender bias, and noted that publication bias had potentially limited unfavourable reports of the procedure under evaluation, justifying their repeat evaluation in a stage 1 study.

For peer review only - http://bmjopen.bmj.com/site/about/guidelines.xhtml

Main theme	Subthemes (if applicable)	Total (n=48)[1]	Author-reported IDEAL stage and number of papers with text coded to theme					
			1 (n=11)	2a (n=18)	2b (n=6)	3 (n=2)	Multiple Stages (n=9)	Not stated (n=2)
Limitations and quality of published evidence	Limited numbers of published studies and small sample sizes	31 (65%)	6	13	5	2	3	2
	Limited outcomes reported	15 (%)	3	5	3	-	3	1
	Limitations of study designs	13 (%)	2	6	1	1	2	1
	Limitations in quality of previous studies	4 (8%)	-	2	1	-	-	1
	Biases in previous studies	2 (4%)	2	-	-	-	-	-
Unknown feasibility and safety outcomes of the procedure	Unknown feasibility outcomes	25 (%)	5	12	2	-	4	2
	Unknown risk or safety outcomes	25 (%)	6	10	3	-	4	2
	Study demonstrates proof of concept for use in humans	2 (4%)	-	-	-	-	2	-
	Outcomes used to determine progression of IDEAL Stage	1 (2%)	-	-	-	-	1	-
Previous use of the procedure in clinical practice	Procedure established in local hospital	2 (4%)	-	1	-	-	1	-
	Procedure is widely used in clinical practice	1 (2%)	-	-	1	-	-	-

[1]Studies may appear in multiple themes.

Table 4. Main themes and sub-themes describing the reported rationale for author reported study design and IDEAL stage.

For peer review only

For peer review only - http://bmjopen.bmj.com/site/about/guidelines.xhtml

(ii) Unknown feasibility and safety outcomes of the procedure

Thirty-two papers highlighted unknown feasibility and safety outcomes. Twenty-five specifically discussed feasibility, including thirteen that stated establishing technical feasibility was the main aim or primary outcome for the current study. These were IDEAL stage 2a (n=7), stage 1 (n=2), stage 2b (n=2) and multistage (n=2) studies. Twenty-five other papers described unknown risk or safety outcomes, the majority of which were stage 2a (n=10) and stage 1 (n=6). Two (80, 81) papers described establishing proof of concept and use in humans, both of which were reported as multiple IDEAL stages, including stage 1. One paper (51), which reported multiple studies across IDEAL stages, used the emergence of safety outcome data as rationale to progress through the IDEAL stages.

(iii) Previous use of the procedure in clinical practice

Three papers described the extent to which procedure had been used in clinical practice, including being "an established technique in our unit" (73) or as "gaining popularity" and as "increasingly being used…worldwide" (55). Another paper (80), reporting multiple IDEAL stages 1 to 3, described the need to evaluate the procedure against standard practice following its adoption by a second centre.

Discussion

In this in-depth examination of how the IDEAL framework has been applied in the published literature, we found that most papers reported early IDEAL stage 1 or 2a case series or case studies, reflecting similar findings from other recent literature reviews examining the use of the IDEAL Framework (8, 9). In these papers, reporting of entirely new procedures or those with substantial modifications were included. Governance approvals were varied and inconsistent across IDEAL stages. IDEAL recommendations specify that research ethical approval is required for stages 1-3. (4). However, around a third of papers did not report seeking any governance approvals, and 13

For peer review only - http://bmjopen.bmj.com/site/about/guidelines.xhtml

reported local or institutional approval, the processes and criteria for which are unclear. This has potential implications for patient harm and transparent consent procedures. Terminology used throughout papers varied and was inconsistent. Terms such as 'innovation' and 'modification' were used without supporting definitions. Some papers described the same procedure as both new, modified, and established (73, 80). Rationales for choice of study design were mainly based on published evidence or the need to assess safety or feasibility outcomes, in line with IDEAL recommendations (4). However, there was some inconsistency with IDEAL recommendations, such as citing the lack of long-term outcomes for the interventions as a reason for evaluating procedures as stage 2a or 2b, despite recommendations that these should measure safety, procedural and short-term outcomes (4). Clearer, more practically applicable methods are needed to identify the stage of innovation to inform study design and governance decisions.

The IDEAL Framework advises that published literature be examined to identify IDEAL stage for an intervention (26). The inconsistent and ambiguous language used in the published literature to describe stage of innovation highlighted in the current review demonstrates that a clearer approach is required. Others have noted this problem with existing definitions of 'innovation' being described as overly broad(82) and lacking in practical utility because they do not distinguish between expected variation in standard clinical practice and changes made to procedures that are evaluated within research(24). Ives *et al* (16) recommend a change in terminology is needed around the term innovation. Rather than using words such as "new, innovative, or modified", Ives recommended that invasive procedures are classified by the level of known risk associated with them based on objective assessments of existing evidence. A new system for categorising published risk for modified or wholly new innovations would allow researchers to map the defined category of risk onto the IDEAL framework to inform study design. The terminology of such a system needs to avoid words that are loaded with inherent bias. The term 'innovation' is synonymous with positive progress and benefit(16, 19), and this may be a factor in influencing research. Positive-outcome-bias is a

For peer review only - http://bmjopen.bmj.com/site/about/guidelines.xhtml

recognised limitation in biomedical research (83-85). This bias may extend to a preference amongst journal editors to publish more eye-catching studies (86, 87). When introducing or evaluating innovations, instead of a focus on ‘how new is this procedure?’, a more appropriate question may be ‘how does this change affect the procedure in terms of risks and benefits, and subsequent impact on outcomes?’(16, 19). Defining and categorising new and modified interventions in terms of degree of risk and evidence of safety and effectiveness, rather than undefined vague language (16, 19) would promote safe and transparent innovation by facilitating the appropriate choice of study design and governance.

In line with recommendations, authors commonly referred to the degree of published evidence as underpinning their choice of study design and IDEAL stage. However, many developments in surgery are not reported in the literature as they are conducted outside of the research framework (17, 88-91). Future work is needed to utilise methods that provide objective assessments of published literature and additional sources (92). This may be through systematic application of frameworks such as GRADE (Grading of Recommendations, Assessment, Development, and Evaluations)(93), for developing and summarising evidence, and by ensuring that a body of evidence is built through an incremental approach from case reports and series through to evidence synthesis in systematic reviews. This is essential to ensure patient safety and shared decision making, share learning and inform evidence based clinical decision making.

Strength and limitations

This review benefits from a robust search and analysis strategy undertaken by a multidisciplinary team of researchers and clinicians. The screening process may have excluded papers which used the IDEAL Framework but did not describe an IDEAL stage in the title or abstract. However, the number of papers identified in this review is comparable to a similar review evaluating the use of the IDEAL

For peer review only - http://bmjopen.bmj.com/site/about/guidelines.xhtml

framework in study design in paper published between 2009 and 2017 (8). Our searches took place in June 2020, more than two years prior to publication. However, since 2020, the Covid-19 pandemic has impacted the progression of many surgical studies (94-96) and the delivery of surgical interventions (97, 98). As such it is unlikely that the volume of IDEAL publications since our searches took place would be of the levels ordinarily expected within that timeframe. We also acknowledge that the introduction of innovative procedures may have been affected by the pandemic and post-pandemic changes to clinical practice, which will not be reflected in this data. However, as this review focusses on the methodology, rationale and terminology used within papers self-reporting as IDEAL stage studies, these factors are not as time dependent as those reviews that examine treatment effects.

Conclusion

This study summarises how the IDEAL framework has been applied in 'real-world' settings for a variety of interventions. It identifies inconsistencies in how authors conceptualise and describe innovation, and how they rationalise their choice of IDEAL stage and study design. Undefined terms were used to describe characteristics of the stage of innovation. It is suggested that innovation in surgery may be better conceptualised in terms of degree of evidence and magnitude of uncertainty about risk and clinical outcomes rather than the nature of the procedure itself. Future work is necessary to develop methods to facilitate this in practical terms and to inform future updates to the IDEAL framework.

Authors' contributions:

J.M.B and N.B conceived the project; R.M, E.H, I.M, M.S & N.A developed and performed systematic review searches and screening; H.S.R, S.C, D.S, D.E & J.M.B developed the analysis approach and

For peer review only

For peer review only - http://bmjopen.bmj.com/site/about/guidelines.xhtml

performed analysis. H.S.R & S.C. wrote the first draft of the manuscript with substantial input from J.M.B, D.S & D.E. H.S.R, S.C, D.S, D.E, R.M, N.B & J.M.B reviewed and reiterated the final manuscript.

Disclosure: The authors declare no conflicts of interest

Funding:

This study was funded by the National Institute for Health and Care Research Bristol Biomedical Research Centre. The views expressed are those of the author(s) and not necessarily those of the NIHR or the Department of Health and Social Care.

Data sharing:

Data relating to qualitative analysis is available upon request.

Acknowledgements:

The authors acknowledge Christin Hoffman, Shelley Potter and Kerry Avery for their contribution to supervising M.S & N.A. We would also like to thank Elizabeth Hudson and Ian Mutanga for their contributions to screening.

REFERENCES

1. Kirkham EN, Main BG, Jones KJB, Blazeby JM, Blencowe NS. Systematic review of the introduction and evaluation of magnetic augmentation of the lower oesophageal sphincter for gastro-oesophageal reflux disease. Br J Surg. 2020;107(1):44-55.
2. Pathak S, Main BG, Blencowe NS, Rees JRE, Robertson HF, Abbadi RAG, et al. A Systematic Review of Minimally Invasive Trans-thoracic Liver Resection to Examine Intervention Description, Governance, and Outcome Reporting of an Innovative Technique. Ann Surg. 2020.
3. Sedrakyan A, Campbell B, Merino JG, Kuntz R, Hirst A, McCulloch P. IDEAL-D: a rational framework for evaluating and regulating the use of medical devices. Bmj. 2016;353:i2372.
4. Hirst A, Philippou Y, Blazeby J, Campbell B, Campbell M, Feinberg J, et al. No Surgical Innovation Without Evaluation: Evolution and Further Development of the IDEAL Framework and Recommendations. 2019;269(2):211-20.
5. Currie A, Brigic A, Blencowe NS, Potter S, Faiz OD, Kennedy RH, et al. Systematic review of surgical innovation reporting in laparoendoscopic colonic polyp resection. Br J Surg. 2015;102(2):e108-16.
6. Muskens IS, Diederen SJH, Senders JT, Zamanipoor Najafabadi AH, van Furth WR, May AM, et al. Innovation in neurosurgery: less than IDEAL? A systematic review. Acta Neurochir (Wien). 2017;159(10):1957-66.

For peer review only - http://bmjopen.bmj.com/site/about/guidelines.xhtml

7. Ergina PL, Cook JA, Blazeby JM, Boutron I, Clavien PA, Reeves BC, et al. Challenges in evaluating surgical innovation. Lancet. 2009;374(9695):1097-104.
8. Khachane A, Philippou Y, Hirst A, McCulloch P. Appraising the uptake and use of the IDEAL Framework and Recommendations: A review of the literature. International journal of surgery (London, England). 2018;57:84-90.
9. Tradewell MB, Albersheim J, Dahm P. Use of the IDEAL framework in the urological literature: where are we in 2018? BJU Int. 2019;123(6):1078-85.
10. Birchley G, Huxtable R, Ives J, Blazeby J. Have We Made Progress in Identifying (Surgical) Innovation? The American Journal of Bioethics. 2019;19(6):25-7.
11. Meyerson D. Innovative surgery and the precautionary principle. J Med Philos. 2013;38(6):605-24.
12. Danjoux NM, Martin DK, Lehoux PN, Harnish JL, Shaul RZ, Bernstein M, et al. Adoption of an innovation to repair aortic aneurysms at a Canadian hospital: a qualitative case study and evaluation. BMC Health Serv Res. 2007;7:182.
13. Lee CB. Surgical innovation and safety: femoroacetabular impingement and the IDEAL collaborative framework. J Hip Preserv Surg. 2016;3(2):89-96.
14. McKneally MF, Daar AS. Introducing new technologies: protecting subjects of surgical innovation and research. World journal of surgery. 2003;27(8):930-4; discussion 4-5.
15. Poulin P, Austen L, Kortbeek JB, Lafreniere R. New technologies and surgical innovation: five years of a local health technology assessment program in a surgical department. Surg Innov. 2012;19(2):187-99.
16. Ives J, Birchley G, Huxtable R, Blazeby J. Hiding behind 'innovation': the case for regulated risk assessment in surgery. Br J Surg. 2022.
17. Biffl WL, Spain DA, Reitsma AM, Minter RM, Upperman J, Wilson M, et al. Responsible development and application of surgical innovations: a position statement of the Society of University Surgeons. J Am Coll Surg. 2008;206(6):1204-9.
18. Jain M, Duh QY, Hirose R, Sosa JA, Suh I. A model for the institutional adoption of innovative surgical techniques. Surgery. 2020;168(2):238-43.
19. Birchley G, Ives J, Huxtable R, Blazeby J. Conceptualising Surgical Innovation: An Eliminativist Proposal. Health Care Anal. 2020;28(1):73-97.
20. Rogers WA, Hutchison K, McNair A. Ethical Issues Across the IDEAL Stages of Surgical Innovation. 2019;269(2):229-33.
21. Hutchison K, Rogers W, Eyers A, Lotz M. Getting Clearer About Surgical Innovation A New Definition and a New Tool to Support Responsible Practice. Annals of Surgery. 2015;262(6):949-54.
22. Zahra J, Paramasivan S, Blencowe NS, Cousins S, Avery K, Mathews J, et al. Discussing surgical innovation with patients: a qualitative study of surgeons' and governance representatives' views. BMJ Open. 2020;10(11):e035251.
23. Mastroianni AC. Liability, regulation and policy in surgical innovation: the cutting edge of research and therapy. Health Matrix Clevel. 2006;16(2):351-442.
24. Rogers WA, Lotz M, Hutchison K, Pourmoslemi A, Eyers A. Identifying surgical innovation: a qualitative study of surgeons' views. Ann Surg. 2014;259(2):273-8.
25. Zaki MM, Cote DJ, Muskens IS, Smith TR, Broekman ML. Defining Innovation in Neurosurgery: Results from an International Survey. World neurosurgery. 2018;114:e1038-e48.
26. Pennell CP, Hirst AD, Campbell WB, Sood A, Agha RA, Barkun JS, et al. Practical guide to the Idea, Development and Exploration stages of the IDEAL Framework and Recommendations. Br J Surg. 2016;103(5):607-15.
27. Macefield RC, Wilson N, Hoffmann C, Blazeby JM, McNair AGK, Avery KNL, et al. Outcome selection, measurement and reporting for new surgical procedures and devices: a systematic review of IDEAL/IDEAL-D studies to inform development of a core outcome set. BJS Open. 2020.

For peer review only

For peer review only - http://bmjopen.bmj.com/site/about/guidelines.xhtml

28. Gale NK, Heath G, Cameron E, Rashid S, Redwood S. Using the framework method for the analysis of qualitative data in multi-disciplinary health research. BMC Med Res Methodol. 2013;13:117.
29. Pennell CP, Hirst A, Sedrakyan A, McCulloch PG. Adapting the IDEAL Framework and Recommendations for medical device evaluation: A modified Delphi survey. International journal of surgery (London, England). 2016;28:141-8.
30. McCulloch P, Cook JA, Altman DG, Heneghan C, Diener MK. IDEAL framework for surgical innovation 1: the idea and development stages. Bmj. 2013;346:f3012.
31. Hirst A, Agha RA, Rosin D, McCulloch P. How can we improve surgical research and innovation?: the IDEAL framework for action. International journal of surgery (London, England). 2013;11(10):1038-42.
32. Ergina PL, Barkun JS, McCulloch P, Cook JA, Altman DG. IDEAL framework for surgical innovation 2: observational studies in the exploration and assessment stages. Bmj. 2013;346:f3011.
33. Cook JA, McCulloch P, Blazeby JM, Beard DJ, Marinac-Dabic D, Sedrakyan A. IDEAL framework for surgical innovation 3: randomised controlled trials in the assessment stage and evaluations in the long term study stage. Bmj. 2013;346:f2820.
34. Barkun JS, Aronson JK, Feldman LS, Maddern GJ, Strasberg SM, Altman DG, et al. Evaluation and stages of surgical innovations. Lancet. 2009;374(9695):1089-96.
35. McCulloch P, Altman DG, Campbell WB, Flum DR, Glasziou P, Marshall JC, et al. No surgical innovation without evaluation: the IDEAL recommendations. Lancet. 2009;374(9695):1105-12.
36. Team TE. EndNote. Philidelphia, PA: Clarivate; 2013.
37. Cousins S, Blencowe NS, Blazeby JM. What is an invasive procedure? A definition to inform study design, evidence synthesis and research tracking. BMJ Open. 2019;9(7):e028576.
38. Harris PA, Taylor R, Minor BL, Elliott V, Fernandez M, O'Neal L, et al. The REDCap consortium: Building an international community of software platform partners. J Biomed Inform. 2019;95:103208.
39. Harris PA, Taylor R, Thielke R, Payne J, Gonzalez N, Conde JG. Research electronic data capture (REDCap)--a metadata-driven methodology and workflow process for providing translational research informatics support. J Biomed Inform. 2009;42(2):377-81.
40. Excellence NIfHaC. The NICE Glossary [Available from: https://www.nice.org.uk/Glossary.
41. Solari V, Jawaid W, Jesudason EC. Enhancing safety of laparoscopic vascular control for neonatal sacrococcygeal teratoma. J Pediatr Surg. 2011;46(5):E5-E7.
42. Diepstraten SCE, Verkooijen HM, van Diest PJ, Veldhuis WB, Fernandez-Gallardo AM, Duvivier KM, et al. Radiofrequency-assisted intact specimen biopsy of breast tumors: critical evaluation according to the IDEAL recommendations. Cancer Imaging. 2011;11(1):247-52.
43. Minervini A, Vanacore D, Vittori G, Milanesi M, Tuccio A, Siena G, et al. Florence robotic intracorporeal neobladder (FloRIN): a new reconfiguration strategy developed following the IDEAL guidelines. Bju International. 2018;121(2):313-7.
44. Greco F, Pini G, Alba S, Altieri VM, Verze P, Mirone V. Minilaparoendoscopic Single-site Pyeloplasty: The Best Compromise Between Surgeon's Ergonomy and Patient's Cosmesis (IDEAL Phase 2a). European Urology Focus. 2016;2(3):319-26.
45. Rischmann P, Gelet A, Riche B, Villers A, Pasticier G, Bondil P, et al. Focal High Intensity Focused Ultrasound of Unilateral Localized Prostate Cancer: A Prospective Multicentric Hemiablation Study of 111 Patients. Eur Urol. 2017;71(2):267-73.
46. Breda A, Territo A, Guttilla A, Sanguedolce F, Manfredi M, Quaresima L, et al. Correlation Between Confocal Laser Endomicroscopy (Cellvizio(®)) and Histological Grading of Upper Tract Urothelial Carcinoma: A Step Forward for a Better Selection of Patients Suitable for Conservative Management. Eur Urol Focus. 2018;4(6):954-9.
47. Bus MTJ, de Bruin DM, Faber DJ, Kamphuis GM, Zondervan PJ, Laguna-Pes MP, et al. Optical Coherence Tomography as a Tool for In Vivo Staging and Grading of Upper Urinary Tract Urothelial Carcinoma: A Study of Diagnostic Accuracy. J Urology. 2016;196(6):1749-55.

For peer review only - http://bmjopen.bmj.com/site/about/guidelines.xhtml

48. Graff P, Portalez D, Lusque A, Brun T, Aziza R, Khalifa J, et al. IDEAL 2a Phase II Study of Ultrafocal Brachytherapy for Low- and Intermediate-risk Prostate Cancer. Int J Radiat Oncol. 2018;102(4):903-11.
49. Liem EIML, Freund JE, Baard J, de Bruin DM, Pes MPL, Savci-Heijink CD, et al. Confocal Laser Endomicroscopy for the Diagnosis of Urothelial Carcinoma in the Bladder and the Upper Urinary Tract: Protocols for Two Prospective Explorative Studies. Jmir Res Protoc. 2018;7(2).
50. Swaan A, Mannaerts CK, Scheltema MJV, Nieuwenhuijzen JA, Savci-Heijink CD, de la Rosette JJMCH, et al. Confocal Laser Endomicroscopy and Optical Coherence Tomography for the Diagnosis of Prostate Cancer: A Needle-Based, In Vivo Feasibility Study Protocol (IDEAL Phase 2A). Jmir Res Protoc. 2018;7(5).
51. Blazeby JM, Blencowe NS, Titcomb DR, Metcalfe C, Hollowood AD, Barham CP. Demonstration of the IDEAL recommendations for evaluating and reporting surgical innovation in minimally invasive oesophagectomy. Brit J Surg. 2011;98(4):544-51.
52. Dumont F, Duchalais E, Aumont A, Thibaudeau E. Cytoreductive surgery plus hyperthermic intraperitoneal chemotherapy by laparoscopy via a single-port approach for low-grade peritoneal malignancy. Surgical Endoscopy and Other Interventional Techniques. 2020;34(6):2789-95.
53. Goh BKP, Prieto M, Syn N, Koh YX, Lim KI. Critical appraisal of the learning curve of minimally invasive hepatectomy: experience with the first 200 cases of a Southeast Asian early adopter. Anz Journal of Surgery. 2020;90(6):1092-8.
54. Inoue Y, Saiura A, Sato T, Ishizawa T, Arita J, Takahashi Y, et al. Laparoscopic pancreatoduodenectomy combined with a novel self-assessment system and feedback discussion: a phase 1 surgical trial following the IDEAL guidelines. Langenbeck Arch Surg. 2016;401(8):1123-30.
55. Stenstra MHBC, van Workum F, van den Wildenberg FJH, Polat F, Rosman C. Evolution of the surgical technique of minimally invasive Ivor-Lewis esophagectomy: description according to the IDEAL framework. Diseases of the Esophagus. 2019;32(3).
56. Gerullis H, Ecke TH, Barski D, Bantel C, Weyland A, Uphoff J, et al. Retrospective analysis of a surgical innovation using the IDEAL framework: radical cystectomy with epidural anaesthesia. J Int Med Res. 2017;45(2):714-22.
57. Gerullis H, Barski D, Georgas E, Boros M, Ramon A, Ecke TH, et al. Protocol for a Randomized Phase II Trial for Mesh Optimization by Autologous Plasma Coating in Prolapse Repair: IDEAL Stage 3. Adv Ther. 2017;34(4):995-1006.
58. Gaboardi F, Pini G, Suardi N, Montorsi F, Passaretti G, Smelzo S. Robotic laparoendoscopic single-site radical prostatectomy (R-LESS-RP) with daVinci Single-Site (R) platform. Concept and evolution of the technique following an IDEAL phase 1. J Robot Surg. 2019;13(2):215-26.
59. Kaouk JH, Haber GP, Autorino R, Crouzet S, Ouzzane A, Flamand V, et al. A Novel Robotic System for Single-port Urologic Surgery: First Clinical Investigation. Eur Urol. 2014;66(6):1033-43.
60. del Val ID, Loureiro C, McCulloch P. The IDEAL prospective development study format for reporting surgical innovations. An illustrative case study of robotic oesophagectomy. International Journal of Surgery. 2015;19:104-11.
61. Barski D, Arndt C, Gerullis H, Yang J, Boros M, Otto T, et al. Transvaginal PVDF-mesh for cystocele repair: A cohort study. International Journal of Surgery. 2017;39:249-54.
62. Versteeg AL, van der Velden JM, Hes J, Eppinga W, Kasperts N, Verkooijen HM, et al. Stereotactic Radiotherapy Followed by Surgical Stabilization Within 24 h for Unstable Spinal Metastases; A Stage I/IIa Study According to the IDEAL Framework. Front Oncol. 2018;8.
63. Mannaerts CK, Wildeboer RR, Postema AW, Hagemann J, Budaus L, Tilki D, et al. Multiparametric ultrasound: evaluation of greyscale, shear wave elastography and contrast-enhanced ultrasound for prostate cancer detection and localization in correlation to radical prostatectomy specimens. Bmc Urol. 2018;18.
64. Barentsz MW, Verkooijen HM, Pijnappel RM, Fernandez MA, van Diest PJ, van der Pol CC, et al. Sentinel lymph node localization with contrast-enhanced ultrasound and an I-125 seed: An ideal prospective development study. International Journal of Surgery. 2015;14:1-6.

For peer review only - http://bmjopen.bmj.com/site/about/guidelines.xhtml

65. Chan JYK, Tsang RK, Holsinger FC, Tong MCF, Ng CWK, Chiu PWY, et al. Prospective clinical trial to evaluate safety and feasibility of using a single port flexible robotic system for transoral head and neck surgery. Oral Oncol. 2019;94:101-5.
66. Hallmann S, Petersein J, Ruttloff J, Ecke TH. Successful evacuation of large perirenal hematoma after extracorporeal shock wave lithotripsy (ESWL) - step 1 of the IDEAL recommendations of surgical innovation. Clin Case Rep. 2017;5(2):123-5.
67. Valerio M, Dickinson L, Ali A, Ramachandran N, Donaldson I, Freeman A, et al. A prospective development study investigating focal irreversible electroporation in men with localised prostate cancer: Nanoknife Electroporation Ablation Trial (NEAT). Contemporary clinical trials. 2014;39(1):57-65.
68. Gerullis H, Schwartmann K, Ecke TH, Georgas E, Herberg P, Winter A, et al. Transvesical Suprapubic Externalization of Ureteral Stents - Introduction of a Surgical Innovation at the Development Stage. Urol Int. 2017;99(1):69-76.
69. Currie AC, Blazeby JM, Suzuki N, Thomas-Gibson S, Reeves B, Morton D, et al. Evaluation of an early-stage innovation for full-thickness excision of benign colonic polyps using the IDEAL framework. Colorectal Disease. 2019;21(9):1004-16.
70. Sharma SK, Momose K, Sedrakyan A, Sonoda T, Sharaiha RZ. Endoscopic stabilization device evaluation using IDEAL framework: A quality improvement study. International Journal of Surgery. 2019;67:18-23.
71. Menon M, Abaza R, Sood A, Ahlawat R, Ghani KR, Jeong W, et al. Robotic Kidney Transplantation with Regional Hypothermia: Evolution of a Novel Procedure Utilizing the IDEAL Guidelines (IDEAL Phase 0 and 1). Eur Urol. 2014;65(5):1001-9.
72. Sood A, McCulloch P, Dahm P, Ahlawat R, Jeong WJ, Bhandari M, et al. Ontogeny of a surgical technique: Robotic kidney transplantation with regional hypothermia. International Journal of Surgery. 2016;25:158-61.
73. Banagala S, Jayarajah U, Almeida I, Samarasekera DN. Efficacy of irrigation tubes in the management of para rectal cavities associated with complex fistula-in-ano. Bmc Surgery. 2018;18.
74. van Kollenburg RAA, van Riel LAMJG, Bloemen PR, Oddens JR, de Reijke TM, Beerlage HP, et al. Transperineal Laser Ablation Treatment for Lower Urinary Tract Symptoms Due to Benign Prostatic Obstruction: Protocol for a Prospective In Vivo Pilot Study. Jmir Res Protoc. 2020;9(1).
75. Harvey KL, Mills N, White P, Holcombe C, Potter S, Steering P-BFS. The Pre-BRA (pre-pectoral Breast Reconstruction EvAluation) feasibility study: protocol for a mixed-methods IDEAL 2a/2b prospective cohort study to determine the safety and effectiveness of prepectoral implant-based breast reconstruction. Bmj Open. 2020;10(1).
76. Buijs M, van Lienden KP, Wagstaff PGK, Scheltema MJV, de Bruin DM, Zondervan PJ, et al. Irreversible Electroporation for the Ablation of Renal Cell Carcinoma: A Prospective, Human, In Vivo Study Protocol (IDEAL Phase 2b). Jmir Res Protoc. 2017;6(2).
77. Villers A, Puech P, Flamand V, Haber GP, Desai MM, Crouzet S, et al. Partial Prostatectomy for Anterior Cancer: Short-term Oncologic and Functional Outcomes. Eur Urol. 2017;72(3):333-42.
78. Barski D, Gerullis H, Ecke T, Varga G, Boros M, Pintelon I, et al. Repair of a vesico-vaginal fistula with amniotic membrane - Step 1 of the IDEAL recommendations of surgical innovation. Cent Eur J Urol. 2015;68(4):459-61.
79. Joukhadar R, Wockel A, Herr D, Paulus V, Radosa J, Hamza A, et al. Challenges of Longevity: Safety of Vaginal and Laparoscopic Urogynecological Procedures in Septuagenarians and Older Patients. Biomed Res Int. 2016;2016.
80. Heikens JT, Gooszen HG, Rovers MM, van Laarhoven CJHM. Stages and Evaluation of Surgical Innovation: A Clinical Example of the Ileo Neorectal Anastomosis After Ulcerative Colitis and Familial Adenomatous Polyposis. Surgical Innovation. 2013;20(5):459-65.
81. Saglam R, Muslumanoglu AY, Tokath Z, Caskurlu T, Sarica K, Tasci AI, et al. A New Robot for Flexible Ureteroscopy: Development and Early Clinical Results (IDEAL Stage 1-2b). Eur Urol. 2014;66(6):1092-100.

For peer review only - http://bmjopen.bmj.com/site/about/guidelines.xhtml

82. Lotz M. Surgical innovation as sui generis surgical research. Theoretical medicine and bioethics. 2013;34(6):447-59.
83. Hasenboehler EA, Choudhry IK, Newman JT, Smith WR, Ziran BH, Stahel PF. Bias towards publishing positive results in orthopedic and general surgery: a patient safety issue? Patient Saf Surg. 2007;1(1):4.
84. Ross JS, Mulvey GK, Hines EM, Nissen SE, Krumholz HM. Trial publication after registration in ClinicalTrials.Gov: a cross-sectional analysis. PLoS Med. 2009;6(9):e1000144.
85. Song F, Parekh S, Hooper L, Loke YK, Ryder J, Sutton AJ, et al. Dissemination and publication of research findings: an updated review of related biases. Health Technol Assess. 2010;14(8):iii, ix-xi, 1-193.
86. Schekman R. How journals like Nature, Cell and Science are damaging science: The Guardian; 2013 [Available from: https://www.theguardian.com/commentisfree/2013/dec/09/how-journals-nature-science-cell-damage-science.
87. Vijayan J, Lachma RN, Mohan Rao PS, Bhat AS. Autologous pericardial aortic valve reconstruction: early results and comparison with mechanical valve replacement. Indian journal of thoracic and cardiovascular surgery. 2020;36(3):186-92.
88. Cousins S, Richards HS, Zahra J, Robertson H, Mathews JA, Avery KNL, et al. Healthcare organization policy recommendations for the governance of surgical innovation: review of NHS policies. Br J Surg. 2022;109(10):1004-12.
89. Stefanidis D, Fanelli RD, Price R, Richardson W, Committee SG. SAGES guidelines for the introduction of new technology and techniques. Surg Endosc. 2014;28(8):2257-71.
90. Surgeons RCo. Surgical Innovation, New Techniques and Technologies. 2019.
91. Surgeons RCo. Learning from invited reviews. 2019.
92. Elliott D, Blencowe NS, Cousins S, Zahra J, Skilton A, Mathews J, et al. Using qualitative research methods to understand how surgical procedures and devices are introduced into NHS hospitals: the Lotus study protocol. BMJ Open. 2021;11(12):e049234.
93. Siemeiniuk RG, G. . What is GRADE? 2023 [Available from: https://bestpractice.bmj.com/info/toolkit/learn-ebm/what-is-grade/.
94. Emamaullee J, Bowdish M, Yan PY, Rodina V, Sher LS. Rapid Adaptation of a Surgical Research Unit to Conduct Clinical Trials During the COVID-19 Pandemic. J Surg Res. 2020;256:76-82.
95. Lupkin S. Coronavirus pandemic Brings Hundreds of U.S. Clinical trials to A Halt. : Heal News from NPR; 2020 [Available from: https://www.npr.org/sections/health-shots/2020/04/11/832210606/coronovirus-pandemic-brings-hundreds-of-u-s-clinical-trials-to-a-halt.
96. Thornton J. Clinical trials suspended in UK to prioritise covid-19 studies and free up staff. Bmj-Brit Med J. 2020;368.
97. Glasbey JC, Ademuyiwa A, Adisa A, AlAmeer E, Arnaud AP, Ayasra F, et al. Effect of COVID-19 pandemic lockdowns on planned cancer surgery for 15 tumour types in 61 countries: an international, prospective, cohort study. Lancet Oncology. 2021;22(11):1507-17.
98. Stoss C, Steffani M, Kohlhaw K, Rudroff C, Staib L, Hartmann D, et al. The COVID-19 pandemic: impact on surgical departments of non-university hospitals. Bmc Surgery. 2020;20(1).

For peer review only

For peer review only - http://bmjopen.bmj.com/site/about/guidelines.xhtml

Supplementary file 1: ENTREQ and PRISMA Checklists

Enhancing transparency in reporting the synthesis of qualitative research (ENTREQ) statement items

No.	Item	Guide and description	Reported on page #
1	Aim	State the research question the synthesis addresses	4
2	Synthesis methodology	Identify the synthesis methodology or theoretical framework which underpins the synthesis, and describe the rationale for choice of methodology (e.g. metaethnography, thematic synthesis, critical interpretive synthesis, grounded theory synthesis, realist synthesis, meta-aggregation, meta-study, framework synthesis).	6-7
3	Approach to searching	Indicate whether the search was pre-planned (comprehensive search strategies to seek all available studies) or iterative (to seek all available concepts until they theoretical saturation is achieved).	4 and Supplementary File 2
4	Inclusion criteria	Specify the inclusion/exclusion criteria (e.g. in terms of population, language, year limits, type of publication, study type).	5
5	Data sources	Describe the information sources used (e.g. electronic databases (MEDLINE, EMBASE, CINAHL, psycINFO, Econlit), grey literature databases (digital thesis, policy reports), relevant organisational websites, experts, information specialists, generic web searches (Google Scholar) hand searching, reference lists) and when the searches conducted; provide the rationale for using the data sources.	4 and Supplementary File 2
6	Electronic Search strategy	Describe the literature search (e.g. provide electronic search strategies with population terms, clinical or health topic terms, experiential or social phenomena related terms, filters for qualitative research, and search limits).	Supplementary File 2
7	Study screening methods	Describe the process of study screening and sifting (e.g. title, abstract and full text review, number of independent reviewers who screened studies).	5
8	Study characteristics	Present the characteristics of the included studies (e.g. year of publication, country, population, number of participants, data collection, methodology, analysis, research questions).	9 & Table 1
9	Study selection results	Identify the number of studies screened and provide reasons for study exclusion (e,g, for comprehensive searching, provide numbers of studies screened and reasons for exclusion indicated in a figure/flowchart; for iterative searching describe reasons	7 & Figure 1

For peer review only

Supplementary file 1: ENTREQ and PRISMA Checklists

		for study exclusion and inclusion based on modifications t the research question and/or contribution to theory development).	
10	Rationale for appraisal	Describe the rationale and approach used to appraise the included studies or selected findings (e.g. assessment of conduct (validity and robustness), assessment of reporting (transparency), assessment of content and utility of the findings).	N/A
11	Appraisal items	State the tools, frameworks and criteria used to appraise the studies or selected findings (e.g. Existing tools: CASP, QARI, COREQ, Mays and Pope [25]; reviewer developed tools; describe the domains assessed: research team, study design, data analysis and interpretations, reporting).	N/A
12	Appraisal process	Indicate whether the appraisal was conducted independently by more than one reviewer and if consensus was required.	N/A
13	Appraisal results	Present results of the quality assessment and indicate which articles, if any, were weighted/excluded based on the assessment and give the rationale.	N/A
14	Data extraction	Indicate which sections of the primary studies were analysed and how were the data extracted from the primary studies? (e.g. all text under the headings "results /conclusions" were extracted electronically and entered into a computer software).	5-6
15	Software	State the computer software used, if any.	6
16	Number of reviewers	Identify who was involved in coding and analysis.	7
17	Coding	Describe the process for coding of data (e.g. line by line coding to search for concepts).	6
18	Study comparison	Describe how were comparisons made within and across studies (e.g. subsequent studies were coded into pre-existing concepts, and new concepts were created when deemed necessary).	7
19	Derivation of themes	Explain whether the process of deriving the themes or constructs was inductive or deductive.	6-7
20	Quotations	Provide quotations from the primary studies to illustrate themes/constructs, and identify whether the quotations were participant quotations of the author's interpretation.	13-17 & Table 2
21	Synthesis output	Present rich, compelling and useful results that go beyond a summary of the primary studies (e.g. new interpretation, models of evidence, conceptual models, analytical framework, development of a new theory or construct).	11-17

For peer review only

Supplementary file 1: ENTREQ and PRISMA Checklists

Section/topic	#	Checklist item	Reported on page #
**TITLE**			
Title	1	Identify the report as a systematic review, meta-analysis, or both.	1
**ABSTRACT**			
Structured summary	2	Provide a structured summary including, as applicable: background; objectives; data sources; study eligibility criteria, participants, and interventions; study appraisal and synthesis methods; results; limitations; conclusions and implications of key findings; systematic review registration number.	1
**INTRODUCTION**			
Rationale	3	Describe the rationale for the review in the context of what is already known.	4
Objectives	4	Provide an explicit statement of questions being addressed with reference to participants, interventions, comparisons, outcomes, and study design (PICOS).	N/A
**METHODS**			
Protocol and registration	5	Indicate if a review protocol exists, if and where it can be accessed (e.g., Web address), and, if available, provide registration information including registration number.	N/A
Eligibility criteria	6	Specify study characteristics (e.g., PICOS, length of follow-up) and report characteristics (e.g., years considered, language, publication status) used as criteria for eligibility, giving rationale.	5
Information sources	7	Describe all information sources (e.g., databases with dates of coverage, contact with study authors to identify additional studies) in the search and date last searched.	4
Search	8	Present full electronic search strategy for at least one database, including any limits used, such that it could be repeated.	Supplementary File 2
Study selection	9	State the process for selecting studies (i.e., screening, eligibility, included in systematic review, and, if applicable, included in the meta-analysis).	5
Data collection process	10	Describe method of data extraction from reports (e.g., piloted forms, independently, in duplicate) and any processes for obtaining and confirming data from investigators.	5-7
Data items	11	List and define all variables for which data were sought (e.g., PICOS, funding sources) and any assumptions and simplifications made.	9-17

For peer review only

Supplementary file 1: ENTREQ and PRISMA Checklists

Risk of bias in individual studies	12	Describe methods used for assessing risk of bias of individual studies (including specification of whether this was done at the study or outcome level), and how this information is to be used in any data synthesis.	N/A
Summary measures	13	State the principal summary measures (e.g., risk ratio, difference in means).	N/A
Synthesis of results	14	Describe the methods of handling data and combining results of studies, if done, including measures of consistency (e.g., I^2) for each meta-analysis.	N/A
Risk of bias across studies	15	Specify any assessment of risk of bias that may affect the cumulative evidence (e.g., publication bias, selective reporting within studies).	N/A
Additional analyses	16	Describe methods of additional analyses (e.g., sensitivity or subgroup analyses, meta-regression), if done, indicating which were pre-specified.	N/A
**RESULTS**			
Study selection	17	Give numbers of studies screened, assessed for eligibility, and included in the review, with reasons for exclusions at each stage, ideally with a flow diagram.	Figure 1
Study characteristics	18	For each study, present characteristics for which data were extracted (e.g., study size, PICOS, follow-up period) and provide the citations.	9-17
Risk of bias within studies	19	Present data on risk of bias of each study and, if available, any outcome level assessment (see item 12).	N/A
Results of individual studies	20	For all outcomes considered (benefits or harms), present, for each study: (a) simple summary data for each intervention group (b) effect estimates and confidence intervals, ideally with a forest plot.	N/A
Synthesis of results	21	Present results of each meta-analysis done, including confidence intervals and measures of consistency.	N/A
Risk of bias across studies	22	Present results of any assessment of risk of bias across studies (see Item 15).	N/A
Additional analysis	23	Give results of additional analyses, if done (e.g., sensitivity or subgroup analyses, meta-regression [see Item 16]).	N/A
**DISCUSSION**			
Summary of evidence	24	Summarize the main findings including the strength of evidence for each main outcome; consider their relevance to key groups (e.g., healthcare providers, users, and policy makers).	17-19
Limitations	25	Discuss limitations at study and outcome level (e.g., risk of bias), and at review-level (e.g., incomplete retrieval of identified research, reporting bias).	19-20
Conclusions	26	Provide a general interpretation of the results in the context of other evidence, and implications for future research.	19-20

For peer review only

Supplementary file 1: ENTREQ and PRISMA Checklists

FUNDING			
Funding	27	Describe sources of funding for the systematic review and other support (e.g., supply of data); role of funders for the systematic review.	20

For peer review only

Supplementary Material 2

Citation searches

Electronic searches in databases with citation tools (web of Science and Scopus) were performed to identify studies reported as following the IDEAL/IDEAL-D framework. This was defined as a study siting any of the ten key IDEAL/IDEAL-D papers that members of the IDEAL collaboration deemed significant in describing the framework (see Khachane *et al*, 2018). No restrictions to study design or publication dates were applied. Searches were conducted in April 2019, and were updated in June 2020.

Web of Science
Search date: 1st April 2019
1018 records identified.
Searches repeated: 26th June 2020 (limited to since 01/04/2019)
200 records identified.

SCOPUS
Search date: 1st April 2019
1112 records identified.
Searches repeated: 26th June 2020 (limited to since 01/04/2019)
259 records identified

Citation searches were conducted for the following papers:

McCulloch P, Altman DG, Campbell WB, Flum DR, Glasziou P, Marshall JC et al. No surgical innovation without evaluation: the IDEAL recommendations. Lancet 2009; 374: 1105–1112.

Sedrakyan A, Campbell B, Merino JG, Kuntz R, Hirst A, McCulloch P. IDEAL-D: a rational framework for evaluating and regulating the use of medical devices. BMJ 2016; 353: i2372.

Barkun JS, Aronson JK, Feldman LS, Maddern GJ, Strasberg SM, Altman DG et al. Evaluation and stages of surgical innovations. Lancet 2009; 374: 1089–1096.

Ergina PL, Cook JA, Blazeby JM, Boutron I, Clavien PA, Reeves BC et al. Challenges in evaluating surgical innovation. Lancet 2009; 374: 1097–1104.

Cook JA, McCulloch P, Blazeby JM, Beard DJ, Marinac-Dabic D, Sedrakyan A; IDEAL Group. IDEAL framework for surgical innovation 3: randomised controlled trials in the assessment stage and evaluations in the long term study stage. BMJ 2013; 346: f2820.

Ergina PL, Barkun JS, McCulloch P, Cook JA, Altman DG; IDEAL Group. IDEAL framework for surgical innovation 2: observational studies in the exploration and assessment stages. BMJ 2013; 346: f3011.

Hirst A, Agha RA, Rosin D, McCulloch P. How can we improve surgical research and innovation?: the IDEAL framework for action. Int J Surg 2013; 11: 1038–1042.

McCulloch P, Cook JA, Altman DG, Heneghan C, Diener MK; IDEAL Group. IDEAL framework for surgical innovation 1: the idea and development stages. BMJ 2013; 346: f3012.

For peer review only

Pennell CP, Hirst A, Sedrakyan A, McCulloch PG. Adapting the IDEAL framework and recommendations for medical device evaluation: a modified Delphi survey. Int J Surg 2016; 28: 141–148.

Pennell CP, Hirst AD, Campbell WB, Sood A, Agha RA, Barkun JS et al. Practical guide to the idea, development and exploration stages of the IDEAL framework and recommendations. Br J Surg 2016; 103: 607–615.

For peer review only

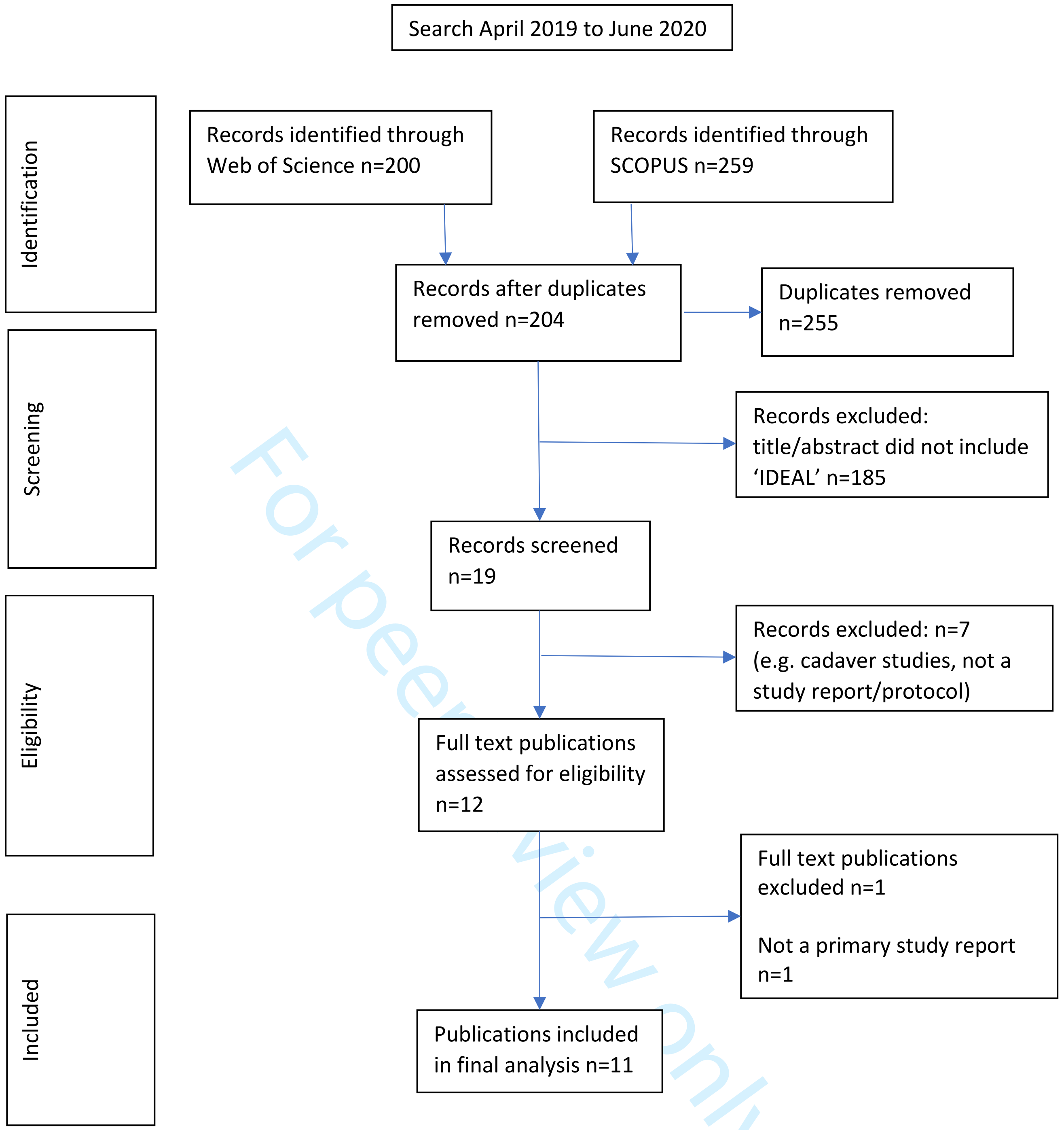
Search April 2019 to June 2020
Identification
Records identified through Web of Science n=200
Records identified through SCOPUS n=259
Records after duplicates removed n=204
Duplicates removed n=255
Screening
Records excluded: title/abstract did not include 'IDEAL' n=185
Records screened n=19
Eligibility
Records excluded: n=7 (e.g. cadaver studies, not a study report/protocol)
Full text publications assessed for eligibility n=12
Full text publications excluded n=1
Not a primary study report n=1
Included
Publications included in final analysis n=11

BMJ Open

Examining the application of the IDEAL Framework in the reporting and evaluation of innovative invasive procedures: secondary qualitative analysis of a systematic review.

Journal:	*BMJ Open*
Manuscript ID	bmjopen-2023-079654.R2
Article Type:	Original research
Date Submitted by the Author:	26-Apr-2024

Complete List of Authors:	Richards, Hollie; University of Bristol Medical School, Population Health Sciences<br>Cousins, Sian; University of Bristol<br>Scroggie, Darren; University of Bristol<br>Elliott, Daisy; University of Bristol<br>Macefield, Rhiannon; University of Bristol Medical School, Population Health Sciences<br>Alford, Natasha; University of Bristol Medical School<br>Shah, Maximilian; University of Bristol Medical School<br>Blencowe, Natalie; University of Bristol Medical School, Population Health Sciences<br>Blazeby, Jane; University of Bristol Medical School, Population Health Sciences

**Primary Subject Heading**:	Surgery
Secondary Subject Heading:	Ethics
Keywords:	SURGERY, QUALITATIVE RESEARCH, Systematic Review

SCHOLARONE™ Manuscripts

I, the Submitting Author has the right to grant and does grant on behalf of all authors of the Work (as defined in the below author licence), an exclusive licence and/or a non-exclusive licence for contributions from authors who are: i) UK Crown employees; ii) where BMJ has agreed a CC-BY licence shall apply, and/or iii) in accordance with the terms applicable for US Federal Government officers or employees acting as part of their official duties; on a worldwide, perpetual, irrevocable, royalty-free basis to BMJ Publishing Group Ltd ("BMJ") its licensees and where the relevant Journal is co-owned by BMJ to the co-owners of the Journal, to publish the Work in this journal and any other BMJ products and to exploit all rights, as set out in our licence.

The Submitting Author accepts and understands that any supply made under these terms is made by BMJ to the Submitting Author unless you are acting as an employee on behalf of your employer or a postgraduate student of an affiliated institution which is paying any applicable article publishing charge ("APC") for Open Access articles. Where the Submitting Author wishes to make the Work available on an Open Access basis (and intends to pay the relevant APC), the terms of reuse of such Open Access shall be governed by a Creative Commons licence – details of these licences and which Creative Commons licence will apply to this Work are set out in our licence referred to above.

Other than as permitted in any relevant BMJ Author's Self Archiving Policies, I confirm this Work has not been accepted for publication elsewhere, is not being considered for publication elsewhere and does not duplicate material already published. I confirm all authors consent to publication of this Work and authorise the granting of this licence.

For peer review only

Examining the application of the IDEAL Framework in the reporting and evaluation of innovative invasive procedures: secondary qualitative analysis of a systematic review.

[1]Richards, H.S*., [1]Cousins, S., [1]Scroggie, D., [1]Elliott, D., [1]Macefield, R., [1]Hudson, E., [1]Mutanga, I., [1]Shah, M., [1]Alford, N., [1]Blencowe, N.S & [1]Blazeby, J.M.

[1]National Institute for Health and Care Research Bristol Biomedical Research Centre, University Hospitals Bristol and Weston NHS Foundation Trust and University of Bristol, Bristol, United Kingdom.

*Corresponding author: Bristol Medical School: Population Health Sciences, University of Bristol, 39 Whatley Road, Bristol, BS8 2PS, United Kingdom. Email: hollie.richards@bristol.ac.uk,

For peer review only

ABSTRACT

Objectives

The development of new surgical procedures is fundamental to advancing patient care. The IDEAL framework describes study designs for stages of innovation. It can be difficult to apply due to challenges in defining and identifying innovative procedures. This study examined how the IDEAL framework is operationalised in real-world settings; specifically, types of innovations evaluated using the framework and how authors justify their choice of IDEAL study design.

Design

Secondary qualitative analysis of a systematic review.

Data Sources

Citation searches (Web of Science and Scopus) identified studies following the IDEAL framework and citing any of the ten key IDEAL/IDEAL_D papers.

Eligibility Criteria

Studies of invasive procedures/devices of any design citing any of the ten key IDEAL/IDEAL_D papers.

Data extraction and synthesis

For peer review only - http://bmjopen.bmj.com/site/about/guidelines.xhtml

All relevant text was extracted. Three frameworks were developed: i) type of innovation under evaluation; ii) terminology used to describe stage of innovation; and iii) reported rationale for IDEAL stage.

Results

Forty-eight articles were included. 19/48 described entirely new procedures, including those used for the first time in a different clinical context (n=15/48), reported as IDEAL stage 2a (n=8, 53%). Terminology describing stage of innovation was varied, inconsistent and ambiguous, and were not defined. Authors justified their choice of IDEAL study design based on limitations in published evidence (n=36) and unknown feasibility and safety (n=32) outcomes.

Conclusion

Identifying stage of innovation is crucial to inform appropriate study design and governance decisions. Authors' rationale for choice of IDEAL stage related to the existing evidence base or lack of sufficient outcome data for procedures. Stage of innovation was poorly defined with inconsistent descriptions. Further work is needed to develop methods to identify innovation to inform practical application of the IDEAL framework. Defining the concept of innovation in terms of uncertainty, risk and degree of evidence may help to inform decision making.

Strengths and limitations of this study

- This work provides an in-depth review of how the IDEAL Framework has been applied and reported by examining how authors self-report stage of innovation in relation to IDEAL and use this to inform study design decisions.
- There are discrepancies in how the IDEAL framework is operationalised in real-world settings, demonstrating the need for improved methods for identifying innovation in surgery.

For peer review only - http://bmjopen.bmj.com/site/about/guidelines.xhtml

- Limitations include the omission of potentially relevant papers where authors did not explicitly reference their use of the IDEAL Framework and that database searches took place in 2020.

INTRODUCTION

Innovations in healthcare requires evidence of safety and effectiveness before widespread implementation. Although pharmaceutical development occurs through a clearly delineated pathway, surgical innovation often happens haphazardly without incremental building of evidence through evaluation within formal research studies (1, 2). The IDEAL (Idea, Developments, Exploration, Assessment and Long-term follow-up) framework was developed to facilitate the evaluation of new surgical procedures and devices (IDEAL-D)(3, 4). It provides study design and governance recommendations for new procedures and devices at different stages of development. Stage 0 deals with pre-clinical studies, prior to stage 1 first in human studies. Stage 1 is followed by stage 2a developmental studies, which focus on technical details and ongoing modifications of the intervention, moving to stage 2b exploratory studies where the intervention and patient group are defined in multiple centres prior to proceeding to stage 3 comparative studies. These are typically randomised controlled trials (RCTs) comparing the now stabilised intervention against standard treatment. Stage 4 consists of long-term monitoring in registries (3, 4). Although IDEAL is an important contribution to the field, it has not been widely applied (1, 5, 6) and is not always used appropriately (7-9). Challenges to the use of the IDEAL recommendations include that this relies on the user determining the stage of innovation of a procedure *a priori*, usually by examining the previous literature, and that 'innovation' in itself is not well defined within the IDEAL Framework (10)

Commonly, definitions of surgical innovation are intrinsically linked to concepts of 'newness', including the extent to which a procedure differs from standard practice (11) or is being modified (11-15). It can also be defined as something with uncertain levels of risk (16). Requirements for additional training

For peer review only - http://bmjopen.bmj.com/site/about/guidelines.xhtml

(13, 15) and the degree of evidence of safety and efficacy (17, 18) have been used to determine how innovative an intervention is. However, some of these definitions are difficult to apply in practice (19). For example, it is often unclear what degree of modification constitutes innovation (10, 20). Guidance has been developed to help surgeons identify innovative procedures (21). The Macquarie Tool bases the degree of innovation on surgeons' previous experiences and how much the procedure varies from routine practice. This places the responsibility for identifying innovation on the surgeon (19), and as such may be open to individual interpretations (19, 22) and biases (23-25). IDEAL suggests that examination of published studies may help determine stage of innovation (26). Although this is useful for stage 1 proof-of-concept studies, where there is very limited or no published evidence about the innovation, the guidance acknowledges that the literature may be of limited use when interventions are at a later stage of innovation. Distinguishing IDEAL stage 2a from 2b may be problematic as centres achieve stability in a procedure at different times (26). Given these challenges, examination of how stage of innovation is determined and used by surgeons and is needed.

A systematic review in 2020 examined outcome selection in IDEAL/IDEAL-D studies to inform a core outcome set for new surgical procedures (27). Following that review, a secondary analysis of identified papers was conducted. The aim of this secondary analysis is to examine how innovative surgical procedures are conceptualised, evaluated and reported using the IDEAL Framework and to understand authors' rationale for choice of IDEAL study design. This will inform work to develop practical methods to facilitate determination of stage of innovation, to ensure appropriate study design and governance (4, 20).

METHODS

A secondary qualitative analysis of papers selected for a previous systematics review(27) was undertaken. Published papers self-reported as IDEAL stage 1 to 4 studies, reporting evaluations of

For peer review only - http://bmjopen.bmj.com/site/about/guidelines.xhtml

innovative surgical procedures, were examined using a qualitative framework approach(28). (See Supplementary File 1 for PRISMA and ENTREQ checklists).

Patient and Public Involvement

Patients and members of the public were not directly involved in this secondary analysis of a systematic review. However, this work took place within the wider context of the Bristol Biomedical Research Centre Surgical and Orthopaedic Innovation Theme, which has engaged extensively with PPIE activities. This includes an active PPIE group involved in the co-production of projects and public engagement events about the definitions of new procedures and how they are introduced into clinical practice.

Searches

Articles identified in a previous systematic review(27) were retrieved. In this review, citation searches of the Web of Science and Scopus databases were undertaken to identify all papers citing any of the ten key IDEAL papers (3, 7, 26, 29-35) published between January 2009 and April 2019 (Supplementary File 2). Records were further filtered to select only those with 'IDEAL' or 'IDEAL-D' in the title or abstract. Searches were updated in June 2020 using the same databases and citation search criteria to identify further studies. Articles were imported into reference management software (36) and duplicates were removed. No further searches were undertaken after 2020 because the current review focussed on authors' conceptualisation and description of innovation, and justification of their choice of study design. Such factors are unlikely to change across the time since the searches were undertaken.

Eligibility criteria and definitions

Eligibility criteria for inclusion in the previous review and updated search were studies of invasive procedures and devices of any design. Secondary studies and those not involving living human participants, were excluded, along with editorials, commentaries, conference proceedings and letters (27). Further exclusion criteria for the current review were studies where the intervention under

For peer review only - http://bmjopen.bmj.com/site/about/guidelines.xhtml

evaluation was not the invasive procedure itself (e.g. non-invasive co-interventions). Invasive procedures were defined as interventions where access to the body was gained by incision, natural orifices or endoscopic techniques, or percutaneous puncture where instrumentation is used in addition to the puncture needle (37). This could include for example a diagnostic endoscopy procedure that does not involve an incision. Invasive devices were defined as any instrument, apparatus or material that is used within or implanted into the body.

Screening

All studies included in the previous review and update were screened to ensure they met the updated eligibility criteria. Titles and abstracts were reviewed initially by one reviewer (HSR) and ineligible studies excluded. Full texts of all remaining articles were then screened. At each stage, 20% of articles were screened by a second reviewer (SC). Disagreements were resolved by discussion amongst the wider study team.

Data extraction and analysis

Study characteristics

Study characteristics were extracted using a standardised REDCap electronic data capture form (38, 39). Author-reported IDEAL stage, year of publication, country, the invasive procedure or device under evaluation, number of centres and surgeons, and sample size are reported in the previous systematic review (27). Additional details about reported oversight (e.g. research governance), study design, and surgical specialty were extracted by one reviewer (HSR). Study designs, determined by NICE definitions (40) were extracted from all papers by two independent reviewers (HSR, SC). To ensure clinical accuracy, data relating to surgical specialties was double-extracted from 20% of papers by a second independent surgeon reviewer (DS). Disagreements were resolved by discussion. Characteristics were reported using descriptive statistics, where appropriate.

For peer review only - http://bmjopen.bmj.com/site/about/guidelines.xhtml

Type of surgical innovation under evaluation and rationale for study design

Papers were analysed using a thematic framework approach (28). This structured approach involves coding of data to identify themes to form a framework, which was applied in an iterative and reflexive manner (28). Full publications were imported into NVivo (version 13) and analysed line by line. Text was coded (whereby text of interest is identified, and descriptive labels attached) using a combination of deductive and inductive approaches, allowing for any unexpected themes to develop. This ensured that analysis was data driven. Three frameworks were developed according to study aims. The first (1) related to the type of innovation under evaluation. All text describing the surgical innovation under evaluation was extracted and coded inductively. For each study, the innovative component under evaluation was identified, for example if the procedure was described as being used for the first time or if changes to a procedure were reported. Where procedural changes were reported, a typology that facilitates the deconstruction of invasive procedures into their constituent parts was utilised to identify how many, and which, steps of the procedure were changed (37). Where codes shared commonalities, these were grouped together into themes and sub-themes to form a framework. Where the innovation under evaluation involved a device or robot, these formed a separate theme. The second (2) framework related to the terminology used to describe the stage of innovation of the procedure. This was analysed according to pre-agreed themes: 'novel/new' – the first or early use of the procedure/device; 'modified' – planned or unplanned changes to the procedure/device or patient group; 'stabilised' – the finalisation of iterative changes or lack of subsequent changes/modifications; and 'adopted' – the wider use of the procedure/device in clinical practice. If the terminology describing stage of innovation could not be coded to these themes, additional themes were created. The third (3) framework related to the reported rationale for study design. All text describing the author's reported rationale for choice of study design was extracted and coded inductively. A narrative summary was produced to illustrate findings with reference to study characteristics.

Framework development

For peer review only - http://bmjopen.bmj.com/site/about/guidelines.xhtml

Three multi-disciplinary (qualitative research (HSR), health services research (SC) and surgical (DS)) members of the team independently coded an initial five articles and discussed how the text had been interpreted. This allowed similarities and differences to be incorporated into an initial set of codes and themes with clear definitions (28). This process was repeated iteratively until agreement was reached on the definitions of codes and themes/sub-themes. A senior member of the team (JMB) reviewed coded text and the iterative development of frameworks. Each framework was then applied to all articles by one researcher (HSR). The research team (HSR, SC, DC and JMB) met regularly throughout this process to discuss analysis, ensuring agreement was reached regarding the appropriate interpretation of the text.

RESULTS

From the 74 papers retrieved from the previous review (27) and update (Supplementary File 3), 48 were included.

For peer review only

Study characteristics		Total, n=48 (%)	Author-reported IDEAL Stage					
			1 (n=11)	2a (n=18)	2b (n=6)	3 (n=2)	Multiple (n=9)	Not stated (n=2)
Study Design	Case series	36 (75)	7	15	6	-	7	1
	Case study	6 (13)	4	1	-	-	-	1
	Cohort study	4 (8)	-	2	-	1	1	-
	Randomised trial	1 (2)	-	-	-	1	-	-
	Multiple study designs	1 (2)	-	-	-	-	1	-
Surgical Specialty	Urology	25 (52)	6	10	4	1	4	-
	Gastrointestinal surgery	7 (15)	1	2	1	1	2	-
	Colorectal surgery	5 (10)	1	2	-	-	2	-
	Gynaecology and obstetrics	2 (4)	1	-	1	-	-	-
	Breast surgery	3 (6)	-	2	-	-	-	1
	Otolaryngology	2 (4)	-	2	-	-	-	-
	Maxillofacial and dental	2 (4)	2	-	-	-	-	-
	Orthopaedics	1 (2)	-	-	-	-	1	-
	Paediatric	1 (2)	-	-	-	-	-	1
Country	European Economic Area (Non-UK)	30 (63)	6	11	6	1	5	1
	Other[1]	9 (19)	2	3	-	1	3	-
	UK	8 (16)	2	4	-	-	1	1
	North America	1 (2)	1	-	-	-	-	-
Publication year	2011 - 2015	12 (25)	3	4	-	-	3	-
	2016 - 2020	36 (75)	8	14	6	2	6	-
Single or multi-centre study	Single centre	41 (85)	11	15	5	1	7	2
	Multi centre	5 (10)	-	2	1	1	1	-
	Not reported	2 (4)	-	1	-	-	1	-
Number of surgeons performing the intervention	1 to 2	22 (46)	6	10	-	2	3	1
	3 to 7	5 (10)	-	2	2	-	1	-
	Not reported/Unclear	21 (44)	5	6	4	-	5	1
Number of participants	1-10	10 (21)	7	2	-	-	-	1
	11-50	26 (54)	4	14	2	-	5	1
	51-100	5 (10)	-	1	1	-	3	-
	101-400	5 (10)	-	1	2	1	1	-
	401-510	2 (4)	-	-	1	1	-	-

Table 1. Characteristics of included studies.

For peer review only

For peer review only - http://bmjopen.bmj.com/site/about/guidelines.xhtml

Study characteristics

Characteristics of included publications are detailed in Table 1. The majority of studies were case series (n=36, 75%) or case studies (n=6, 13%). Most studies were reported as IDEAL stage 2a (n=18) although two did not explicitly state a stage: one stated it was stage 1 or 2a (41) and the other self-reported as an early-stage assessment of technical problems during the learning curve “in line with the IDEAL recommendations” (42). Nine papers reported the progression of an intervention through multiple IDEAL stages.

Table 2 details reported governance arrangements. Eighteen (36%) reported research governance, including approval from Institutional Review Boards or Research Ethics Committees overseen by the U.S. Food and Drug Administration (FDA) or the U.K NHS Health Research Authority (HRA). Most of these were reported as stage 2a (n=7). Thirteen (27%) papers reported obtaining local/institutional approval, using a variety of terms such as “local ethical committee” and “institutional medical ethical committee” to describe them. Five (10%) papers, including three reported as IDEAL stage 1, stated that ethical approval was not required or obtained. The remaining twelve (25%) papers, including IDEAL stage 1 (n=3) and stage 2a (n=5), did not report any governance approvals.

For peer review only - http://bmjopen.bmj.com/site/about/guidelines.xhtml

Reported governance approvals		Total (%) N=48	Author-reported IDEAL Stage					
			1 (n=11)	2a (n=18)	2b (n=6)	3 (n=2)	Multiple (n=9)	Not stated (n=2)
Formal research governance	Institutional Review Board (IRB)	14	4	5	2	1	2	-
	“REC ethical approval”	1	-	1	-	-	-	-
	“REC ethical approval (& NPC)”	1	-	1	-	-	-	-
	“Regional ethics commissions”	1	-	-	-	1	-	-
	“National ethics committee”	1	-	-	1	-	-	-
Local or institutional approvals	“Local ethical committee”	2	-	1	1	-	-	-
	“Local ethics board”	1	-	-	-	-	1	-
	“Local ethics review committee”	1	-	1	-	-	-	-
	“Local medical ethical committee”	2	-	1	1	-	-	-
	“Local research ethics committee”	3	1	1	-	-	1	-
	“Institution ethical committee”	1	-	1	-	-	-	-
	“Institutional health sciences research ethics board”	1	-	-	-	-	1	-
	“Institutional medical ethical committee”	1	-	-	-	-	1	-
	“Ethical commission of medical school”	1	-	-	-	-	1	-
No ethical approval obtained	"Current study: no ethical approval."	1	-	1	-	-	-	-
	“Audit - ethics not required”	1	-	-	-	-	1	-
	“Ethical approval not required”	1	1	-	-	-	-	-
	“No further ethics approval required.”	1	1	-	-	-	-	-
	“Not needed due to retrospective design”	1	1	-	-	-	-	-
Not reported	N/A	12	3	5	1	-	1	2

Table 2. Governance approvals reported by 48 included studies.

For peer review only

For peer review only - http://bmjopen.bmj.com/site/about/guidelines.xhtml

(1) Type of innovation under evaluation

The types of innovations under evaluation were categorised into five themes related to 'newness' (Table 3); i) an entire new procedure (this included if a procedure had two or more new steps; n=19); ii) one step of the procedure is new; iii) a new device including robotic approach; iv) changes to the sequence of procedure components; and v) changes to the combination of procedure components.

i) An entire new procedure

Nineteen (39%) papers reported an entire new procedure. Most (n=15) referred to procedures being newly used in a different clinical context, for example, combining existing minimally invasive techniques into a new type of reconstruction or anastomosis in bowel (43) and urological (44) surgery. Of these, 8 were classified as IDEAL 1a. Three (6%) other papers described procedures being applied to a different clinical indication, for example high intensity focused ultrasound applied to focal therapy for prostate tumours (45).

ii) One step of the procedure is new

Nineteen other papers described procedures with one step changed. Most (n=8) were stage 2a. Ten involved one step of a procedure being applied to different clinical indication, for example an existing invasive imaging technique currently used for one type of cancer being used for a different type of cancer (46-50). Six described changes to access to a body cavity by using minimally invasive techniques instead of open surgery (41, 51-55). One paper described the application of one step of an invasive procedure to a different patient group (56).

iii) A new device including robotic approach

Eight (17%) papers described the evaluation of an invasive device/robot. Of these, four described changes to one component of an invasive device (n=1) or robot (n=3). These were a modification to the coating of a vaginal implant device (57), a new access port for use with a robot devices (58, 59) and changing part of the procedure from open to robotic (60). These were reported as IDEAL stage 1 (n=2), stage 2a (n=1) or stage 3 (n=1) respectively. Three papers described the development of an entirely new invasive device (n=1) or robot (n=2). (61).

For peer review only - http://bmjopen.bmj.com/site/about/guidelines.xhtml

Main theme	Subthemes (if applicable)	Total (n=48)	Author-reported IDEAL stage and number of papers with text coded to theme					
			1 (n=11)	2a (n=18)	2b (n=6)	3 (n=2)	Multiple Stages (n=9)	Not stated (n=2)
An entire new procedure	Procedure used for the first time in that clinical context	15 (31%)	3	8	-	-	3	1
	Procedure applied to a different clinical indication	3 (6%)	1	-	1	-	1	-
	Procedure previously used in different patient group	1 (2%)	-	-	1	-	-	-
One step of the procedure is new	Step of procedure applied to a different clinical indication	10 (21%)	-	7	2	-	1	-
	Change to access	6 (12%)	1	-	1	1	2	1
	Change to reconstruction	2 (4%)	1	1	-	-	-	-
	Step of a procedure previously used in different patient group	1 (2%)	1	-	-	-	-	-
A new device including robotic approach	Change to one component of a device/robotic instruments	4 (8%)	2	1	-	1	-	-
	Development of an entire device/robotic instruments for one component of procedure	3 (6%)	1	1	-	-	1	-
	Existing device applied to a different clinical indication	1 (2%)	1	-	-	-	-	-
Novel changes to the sequence of procedure components	N/A	1 (2%)	-	-	-	-	1	-
Changes to the combination of procedure components	N/A	1 (2%)	-	-	1	-	-	-

Table 3. Main themes and sub-themes describing the type of innovation, by author-reported IDEAL stage and in total.

For peer review only

For peer review only - http://bmjopen.bmj.com/site/about/guidelines.xhtml

iv) Changes to the sequence of procedure components

One paper reporting a sequential IDEAL stages 2a and 2b study evaluated changes to the timing of radiotherapy for unstable spinal metastases (62).

v) Changes to the combination of procedure components

One IDEAL stage 2b study described an evaluation of combined versus individual invasive diagnostic tests (63).

For peer review only

(2) Terminology used to describe stage of innovation

In addition to the four *a priori* themes, the theme of abandoned procedures was identified during data analyses. The final five themes, (i) new/novel; ii) modified; iii) stabilised; iv) adopted; and v) abandoned procedures, are summarised below.

i) New/novel

There was a lack of consistency across papers reporting all IDEAL stages in terms describing the 'newness' of the procedure. Commonly terms included 'innovation' (n=18), 'new' (n=14), 'novel' (n=13), 'introduction' (n=4) and 'first-in-human' (n=4). 'Innovation' was used in papers reporting all IDEAL stages, whereas 'introduction' was predominantly used in stage 1 papers (n=3). None of the papers defined these terms. 'First-in-human' was used in one IDEAL stage 1 study, one stage 3 and two multistage studies, but in these latter cases it described the earlier development of the innovation under evaluation. Twenty studies used terminology and phrases related to the first report of the innovation (e.g. "this is the first study" (64), "first clinical trial" (65) and "the first case" (66)). The majority of these were stage 1 (n=7, 16%) and stage 2a (n=8, 17%). Five papers described the procedure as "emerging" (41) or "promising" (67). These were reported as IDEAL stage 2a (n=3), 2b (n=1) or not stated (n=1).

ii) Modified

For peer review only - http://bmjopen.bmj.com/site/about/guidelines.xhtml

Twenty-three papers, mostly early stage 1-2b, used a variety of terms to describe modifications. Although the terms 'modified' or 'modification' were used in eleven (23%) papers, most (n=18, 38%) described modifications in terms of the evolution and ongoing development of the procedure. This included the "evolution of the new method" (68), describing the procedure as "still under development" (69). Ten (21%) papers described changes based on their experience, e.g. "from case 35 onward" (61), "based on the results of patient 1" (54) and "thereafter we noticed" (58). These tended to appear in stage 2a (n=4) and stage 1 (n=3) studies. Some papers used more precise terms, such as 'refinements' (n=7, 16%), 'changes' (n=5, 11%) and 'adaptions/adjustments/alterations' (n=4, 8%). Others were vaguer. Terms included "hybrid solutions" (58) and "continued…optimization" (70). Additional undefined terms for modifications included "experimented" (71) and "improvised" (72), and descriptions of steps of the procedure being "replaced" (55) or "tried [and] tested" (72).

iii) Stabilised

Eight papers described the procedure using terms synonymous with stabilised (n=2 stage 1; n=3 stage 2a; n=1 stage 3; n=2 multiple stages). Terms and phrases included having "achieved stability" (70), "appear[ing] stable" (69) or "standardized" (58). Some authors were slightly more specific and described when procedures were no longer undergoing modifications, in two cases explicitly stating that this was an indication of stabilisation as "all surgeons began learning" the procedure (51) or it "had entered IDEAL stage 3" (55).

iv) Adopted

Four papers described the procedure being adopted into clinical practice (n=2 stage 2a; n=1 stage 3; n=1 multiple stages). One said the procedure was "an established technique in our unit" (73) and another said the procedure "may become a standard" (46) upon wider adoption.

v) Abandoned

For peer review only - http://bmjopen.bmj.com/site/about/guidelines.xhtml

Three papers stated that the procedure had been abandoned (n=1 stage 2a; n=2 multiple stages), including descriptions of the procedure as “not a viable alternative” (64) or as being halted due to “loss of confidence” (51).

(3) Reported rationale for study design

Three main themes were identified (Table 4): i) the limitations and quality of previously published evidence; ii) unknown feasibility and safety outcomes; and iii) previous use in clinical practice.

(i) The limitations and quality of published evidence

Thirty-six papers described the limitations and quality of previously published evidence as a rationale for the current study design. Of these, 31 discussed the limited number of published studies evaluating the procedure. These authors highlighted the small numbers of participants and surgeons in previous studies (e.g. “treated 18 patients” (74) and “a few expert surgeons” (75)). Fifteen other papers highlighted limitations in the types of outcomes collected in previous studies and three (74, 76, 77) (6%) (n=2 stage 2a; n=1 stage 2b) highlighted a lack of long-term outcomes as rationale for the current study. Three papers (44, 55, 78) (stage 1, stage 2a and stage 2b) stated that the procedure was not standardised. Other papers (n=13) focused on previous study designs and stated that there were few prospective studies, and that most evidence was from case series, case control studies, proof-of-concept or animal model studies. Of these, 10 were reported as stage 2a (n=8) or stage 1 (n=2) studies. In addition, four papers (48, 69, 75, 79) highlighted that findings from previous studies were inconsistent, inconclusive or conflicting, suggesting further evaluation was required. Another two stage 1 papers (54, 56) commented on potential biases in previous studies, specifically gender bias, and noted that publication bias had potentially limited unfavourable reports of the procedure under evaluation, justifying their repeat evaluation in a stage 1 study.

For peer review only

Main theme	Subthemes (if applicable)	Total (n=48)[1]	Author-reported IDEAL stage and number of papers with text coded to theme					
			1 (n=11)	2a (n=18)	2b (n=6)	3 (n=2)	Multiple Stages (n=9)	Not stated (n=2)
Limitations and quality of published evidence	Limited numbers of published studies and small sample sizes	31 (65%)	6	13	5	2	3	2
	Limited outcomes reported	15 (%)	3	5	3	-	3	1
	Limitations of study designs	13 (%)	2	6	1	1	2	1
	Limitations in quality of previous studies	4 (8%)	-	2	1	-	-	1
	Biases in previous studies	2 (4%)	2	-	-	-	-	-
Unknown feasibility and safety outcomes of the procedure	Unknown feasibility outcomes	25 (%)	5	12	2	-	4	2
	Unknown risk or safety outcomes	25 (%)	6	10	3	-	4	2
	Study demonstrates proof of concept for use in humans	2 (4%)	-	-	-	-	2	-
	Outcomes used to determine progression of IDEAL Stage	1 (2%)	-	-	-	-	1	-
Previous use of the procedure in clinical practice	Procedure established in local hospital	2 (4%)	-	1	-	-	1	-
	Procedure is widely used in clinical practice	1 (2%)	-	-	1	-	-	-

[1]Studies may appear in multiple themes.

Table 4. Main themes and sub-themes describing the reported rationale for author reported study design and IDEAL stage.

For peer review only

For peer review only - http://bmjopen.bmj.com/site/about/guidelines.xhtml

(ii) Unknown feasibility and safety outcomes of the procedure

Thirty-two papers highlighted unknown feasibility and safety outcomes. Twenty-five specifically discussed feasibility, including thirteen that stated establishing technical feasibility was the main aim or primary outcome for the current study. These were IDEAL stage 2a (n=7), stage 1 (n=2), stage 2b (n=2) and multistage (n=2) studies. Twenty-five other papers described unknown risk or safety outcomes, the majority of which were stage 2a (n=10) and stage 1 (n=6). Two (80, 81) papers described establishing proof of concept and use in humans, both of which were reported as multiple IDEAL stages, including stage 1. One paper (51), which reported multiple studies across IDEAL stages, used the emergence of safety outcome data as rationale to progress through the IDEAL stages.

(iii) Previous use of the procedure in clinical practice

Three papers described the extent to which procedure had been used in clinical practice, including being "an established technique in our unit" (73) or as "gaining popularity" and as "increasingly being used…worldwide" (55). Another paper (80), reporting multiple IDEAL stages 1 to 3, described the need to evaluate the procedure against standard practice following its adoption by a second centre.

Discussion

In this in-depth examination of how the IDEAL framework has been applied in the published literature, we found that most papers reported early IDEAL stage 1 or 2a case series or case studies, reflecting similar findings from other recent literature reviews examining the use of the IDEAL Framework (8, 9). In these papers, reporting of entirely new procedures or those with substantial modifications were included. Governance approvals were varied and inconsistent across IDEAL stages. IDEAL recommendations specify that research ethical approval is required for stages 1-3. (4). However, around a third of papers did not report seeking any governance approvals, and 13

For peer review only - http://bmjopen.bmj.com/site/about/guidelines.xhtml

reported local or institutional approval, the processes and criteria for which are unclear. This has potential implications for patient harm and transparent consent procedures. Terminology used throughout papers varied and was inconsistent. Terms such as 'innovation' and 'modification' were used without supporting definitions. Some papers described the same procedure as both new, modified, and established (73, 80). Rationales for choice of study design were mainly based on published evidence or the need to assess safety or feasibility outcomes, in line with IDEAL recommendations (4). However, there was some inconsistency with IDEAL recommendations, such as citing the lack of long-term outcomes for the interventions as a reason for evaluating procedures as stage 2a or 2b, despite recommendations that these should measure safety, procedural and short-term outcomes (4). Clearer, more practically applicable methods are needed to identify the stage of innovation to inform study design and governance decisions.

The IDEAL Framework advises that published literature be examined to identify IDEAL stage for an intervention (26). The inconsistent and ambiguous language used in the published literature to describe stage of innovation highlighted in the current review demonstrates that a clearer approach is required. Others have noted this problem with existing definitions of 'innovation' being described as overly broad(82) and lacking in practical utility because they do not distinguish between expected variation in standard clinical practice and changes made to procedures that are evaluated within research(24). Ives *et al* (16) recommend a change in terminology is needed around the term innovation. Rather than using words such as "new, innovative, or modified", Ives recommended that invasive procedures are classified by the level of known risk associated with them based on objective assessments of existing evidence. A new system for categorising published risk for modified or wholly new innovations would allow researchers to map the defined category of risk onto the IDEAL framework to inform study design. The terminology of such a system needs to avoid words that are loaded with inherent bias. The term 'innovation' is synonymous with positive progress and benefit(16, 19), and this may be a factor in influencing research. Positive-outcome-bias is a

For peer review only - http://bmjopen.bmj.com/site/about/guidelines.xhtml

For peer review only

recognised limitation in biomedical research (83-85). This bias may extend to a preference amongst journal editors to publish more eye-catching studies (86, 87). When introducing or evaluating innovations, instead of a focus on 'how new is this procedure?', a more appropriate question may be 'how does this change affect the procedure in terms of risks and benefits, and subsequent impact on outcomes?'(16, 19). Defining and categorising new and modified interventions in terms of degree of risk and evidence of safety and effectiveness, rather than undefined vague language (16, 19) would promote safe and transparent innovation by facilitating the appropriate choice of study design and governance.

In line with recommendations, authors commonly referred to the degree of published evidence as underpinning their choice of study design and IDEAL stage. However, many developments in surgery are not reported in the literature as they are conducted outside of the research framework (17, 88-91). Future work is needed to utilise methods that provide objective assessments of published literature and additional sources (92). This may be through systematic application of frameworks such as GRADE (Grading of Recommendations, Assessment, Development, and Evaluations)(93), for developing and summarising evidence, and by ensuring that a body of evidence is built through an incremental approach from case reports and series through to evidence synthesis in systematic reviews. This is essential to ensure patient safety and shared decision making, share learning and inform evidence based clinical decision making.

Strength and limitations

This review benefits from a robust search and analysis strategy undertaken by a multidisciplinary team of researchers and clinicians. The screening process may have excluded papers which used the IDEAL Framework but did not describe an IDEAL stage in the title or abstract. However, the number of papers identified in this review is comparable to a similar review evaluating the use of the IDEAL

For peer review only - http://bmjopen.bmj.com/site/about/guidelines.xhtml

framework in study design in paper published between 2009 and 2017 (8). Our searches took place in June 2020, more than two years prior to publication. However, since 2020, the Covid-19 pandemic has impacted the progression of many surgical studies (94-96) and the delivery of surgical interventions (97, 98). As such it is unlikely that the volume of IDEAL publications since our searches took place would be of the levels ordinarily expected within that timeframe. We also acknowledge that the introduction of innovative procedures may have been affected by the pandemic and post-pandemic changes to clinical practice, which will not be reflected in this data. However, as this review focusses on the methodology, rationale and terminology used within papers self-reporting as IDEAL stage studies, these factors are not as time dependent as those reviews that examine treatment effects.

Conclusion

This study summarises how the IDEAL framework has been applied in 'real-world' settings for a variety of interventions. It identifies inconsistencies in how authors conceptualise and describe innovation, and how they rationalise their choice of IDEAL stage and study design. Undefined terms were used to describe characteristics of the stage of innovation. It is suggested that innovation in surgery may be better conceptualised in terms of degree of evidence and magnitude of uncertainty about risk and clinical outcomes rather than the nature of the procedure itself. Future work is necessary to develop methods to facilitate this in practical terms and to inform future updates to the IDEAL framework.

Authors' contributions:

J.M.B and N.B conceived the project; R.M, E.H, I.M, M.S & N.A developed and performed systematic review searches and screening; H.S.R, S.C, D.S, D.E & J.M.B developed the analysis approach and

For peer review only - http://bmjopen.bmj.com/site/about/guidelines.xhtml

For peer review only

performed analysis. H.S.R & S.C. wrote the first draft of the manuscript with substantial input from J.M.B, D.S & D.E. H.S.R, S.C, D.S, D.E, R.M, N.B & J.M.B reviewed and reiterated the final manuscript.

Disclosure: The authors declare no conflicts of interest

Funding:

This study was funded by the National Institute for Health and Care Research Bristol Biomedical Research Centre. The views expressed are those of the author(s) and not necessarily those of the NIHR or the Department of Health and Social Care.

Data sharing:

Data relating to qualitative analysis is available upon request.

Acknowledgements:

The authors acknowledge Christin Hoffman, Shelley Potter and Kerry Avery for their contribution to supervising M.S & N.A. We would also like to thank Elizabeth Hudson and Ian Mutanga for their contributions to screening.

For peer review only

REFERENCES

1. Kirkham EN, Main BG, Jones KJB, Blazeby JM, Blencowe NS. Systematic review of the introduction and evaluation of magnetic augmentation of the lower oesophageal sphincter for gastro-oesophageal reflux disease. Br J Surg. 2020;107(1):44-55.
2. Pathak S, Main BG, Blencowe NS, Rees JRE, Robertson HF, Abbadi RAG, et al. A Systematic Review of Minimally Invasive Trans-thoracic Liver Resection to Examine Intervention Description, Governance, and Outcome Reporting of an Innovative Technique. Ann Surg. 2020.
3. Sedrakyan A, Campbell B, Merino JG, Kuntz R, Hirst A, McCulloch P. IDEAL-D: a rational framework for evaluating and regulating the use of medical devices. Bmj. 2016;353:i2372.
4. Hirst A, Philippou Y, Blazeby J, Campbell B, Campbell M, Feinberg J, et al. No Surgical Innovation Without Evaluation: Evolution and Further Development of the IDEAL Framework and Recommendations. 2019;269(2):211-20.
5. Currie A, Brigic A, Blencowe NS, Potter S, Faiz OD, Kennedy RH, et al. Systematic review of surgical innovation reporting in laparoendoscopic colonic polyp resection. Br J Surg. 2015;102(2):e108-16.
6. Muskens IS, Diederen SJH, Senders JT, Zamanipoor Najafabadi AH, van Furth WR, May AM, et al. Innovation in neurosurgery: less than IDEAL? A systematic review. Acta Neurochir (Wien). 2017;159(10):1957-66.

For peer review only - http://bmjopen.bmj.com/site/about/guidelines.xhtml

7. Ergina PL, Cook JA, Blazeby JM, Boutron I, Clavien PA, Reeves BC, et al. Challenges in evaluating surgical innovation. Lancet. 2009;374(9695):1097-104.
8. Khachane A, Philippou Y, Hirst A, McCulloch P. Appraising the uptake and use of the IDEAL Framework and Recommendations: A review of the literature. International journal of surgery (London, England). 2018;57:84-90.
9. Tradewell MB, Albersheim J, Dahm P. Use of the IDEAL framework in the urological literature: where are we in 2018? BJU Int. 2019;123(6):1078-85.
10. Birchley G, Huxtable R, Ives J, Blazeby J. Have We Made Progress in Identifying (Surgical) Innovation? The American Journal of Bioethics. 2019;19(6):25-7.
11. Meyerson D. Innovative surgery and the precautionary principle. J Med Philos. 2013;38(6):605-24.
12. Danjoux NM, Martin DK, Lehoux PN, Harnish JL, Shaul RZ, Bernstein M, et al. Adoption of an innovation to repair aortic aneurysms at a Canadian hospital: a qualitative case study and evaluation. BMC Health Serv Res. 2007;7:182.
13. Lee CB. Surgical innovation and safety: femoroacetabular impingement and the IDEAL collaborative framework. J Hip Preserv Surg. 2016;3(2):89-96.
14. McKneally MF, Daar AS. Introducing new technologies: protecting subjects of surgical innovation and research. World journal of surgery. 2003;27(8):930-4; discussion 4-5.
15. Poulin P, Austen L, Kortbeek JB, Lafreniere R. New technologies and surgical innovation: five years of a local health technology assessment program in a surgical department. Surg Innov. 2012;19(2):187-99.
16. Ives J, Birchley G, Huxtable R, Blazeby J. Hiding behind 'innovation': the case for regulated risk assessment in surgery. Br J Surg. 2022.
17. Biffl WL, Spain DA, Reitsma AM, Minter RM, Upperman J, Wilson M, et al. Responsible development and application of surgical innovations: a position statement of the Society of University Surgeons. J Am Coll Surg. 2008;206(6):1204-9.
18. Jain M, Duh QY, Hirose R, Sosa JA, Suh I. A model for the institutional adoption of innovative surgical techniques. Surgery. 2020;168(2):238-43.
19. Birchley G, Ives J, Huxtable R, Blazeby J. Conceptualising Surgical Innovation: An Eliminativist Proposal. Health Care Anal. 2020;28(1):73-97.
20. Rogers WA, Hutchison K, McNair A. Ethical Issues Across the IDEAL Stages of Surgical Innovation. 2019;269(2):229-33.
21. Hutchison K, Rogers W, Eyers A, Lotz M. Getting Clearer About Surgical Innovation A New Definition and a New Tool to Support Responsible Practice. Annals of Surgery. 2015;262(6):949-54.
22. Zahra J, Paramasivan S, Blencowe NS, Cousins S, Avery K, Mathews J, et al. Discussing surgical innovation with patients: a qualitative study of surgeons' and governance representatives' views. BMJ Open. 2020;10(11):e035251.
23. Mastroianni AC. Liability, regulation and policy in surgical innovation: the cutting edge of research and therapy. Health Matrix Clevel. 2006;16(2):351-442.
24. Rogers WA, Lotz M, Hutchison K, Pourmoslemi A, Eyers A. Identifying surgical innovation: a qualitative study of surgeons' views. Ann Surg. 2014;259(2):273-8.
25. Zaki MM, Cote DJ, Muskens IS, Smith TR, Broekman ML. Defining Innovation in Neurosurgery: Results from an International Survey. World neurosurgery. 2018;114:e1038-e48.
26. Pennell CP, Hirst AD, Campbell WB, Sood A, Agha RA, Barkun JS, et al. Practical guide to the Idea, Development and Exploration stages of the IDEAL Framework and Recommendations. Br J Surg. 2016;103(5):607-15.
27. Macefield RC, Wilson N, Hoffmann C, Blazeby JM, McNair AGK, Avery KNL, et al. Outcome selection, measurement and reporting for new surgical procedures and devices: a systematic review of IDEAL/IDEAL-D studies to inform development of a core outcome set. BJS Open. 2020.

For peer review only - http://bmjopen.bmj.com/site/about/guidelines.xhtml

28. Gale NK, Heath G, Cameron E, Rashid S, Redwood S. Using the framework method for the analysis of qualitative data in multi-disciplinary health research. BMC Med Res Methodol. 2013;13:117.
29. Pennell CP, Hirst A, Sedrakyan A, McCulloch PG. Adapting the IDEAL Framework and Recommendations for medical device evaluation: A modified Delphi survey. International journal of surgery (London, England). 2016;28:141-8.
30. McCulloch P, Cook JA, Altman DG, Heneghan C, Diener MK. IDEAL framework for surgical innovation 1: the idea and development stages. Bmj. 2013;346:f3012.
31. Hirst A, Agha RA, Rosin D, McCulloch P. How can we improve surgical research and innovation?: the IDEAL framework for action. International journal of surgery (London, England). 2013;11(10):1038-42.
32. Ergina PL, Barkun JS, McCulloch P, Cook JA, Altman DG. IDEAL framework for surgical innovation 2: observational studies in the exploration and assessment stages. Bmj. 2013;346:f3011.
33. Cook JA, McCulloch P, Blazeby JM, Beard DJ, Marinac-Dabic D, Sedrakyan A. IDEAL framework for surgical innovation 3: randomised controlled trials in the assessment stage and evaluations in the long term study stage. Bmj. 2013;346:f2820.
34. Barkun JS, Aronson JK, Feldman LS, Maddern GJ, Strasberg SM, Altman DG, et al. Evaluation and stages of surgical innovations. Lancet. 2009;374(9695):1089-96.
35. McCulloch P, Altman DG, Campbell WB, Flum DR, Glasziou P, Marshall JC, et al. No surgical innovation without evaluation: the IDEAL recommendations. Lancet. 2009;374(9695):1105-12.
36. Team TE. EndNote. Philidelphia, PA: Clarivate; 2013.
37. Cousins S, Blencowe NS, Blazeby JM. What is an invasive procedure? A definition to inform study design, evidence synthesis and research tracking. BMJ Open. 2019;9(7):e028576.
38. Harris PA, Taylor R, Minor BL, Elliott V, Fernandez M, O'Neal L, et al. The REDCap consortium: Building an international community of software platform partners. J Biomed Inform. 2019;95:103208.
39. Harris PA, Taylor R, Thielke R, Payne J, Gonzalez N, Conde JG. Research electronic data capture (REDCap)--a metadata-driven methodology and workflow process for providing translational research informatics support. J Biomed Inform. 2009;42(2):377-81.
40. Excellence NIfHaC. The NICE Glossary [Available from: https://www.nice.org.uk/Glossary.
41. Solari V, Jawaid W, Jesudason EC. Enhancing safety of laparoscopic vascular control for neonatal sacrococcygeal teratoma. J Pediatr Surg. 2011;46(5):E5-E7.
42. Diepstraten SCE, Verkooijen HM, van Diest PJ, Veldhuis WB, Fernandez-Gallardo AM, Duvivier KM, et al. Radiofrequency-assisted intact specimen biopsy of breast tumors: critical evaluation according to the IDEAL recommendations. Cancer Imaging. 2011;11(1):247-52.
43. Minervini A, Vanacore D, Vittori G, Milanesi M, Tuccio A, Siena G, et al. Florence robotic intracorporeal neobladder (FloRIN): a new reconfiguration strategy developed following the IDEAL guidelines. Bju International. 2018;121(2):313-7.
44. Greco F, Pini G, Alba S, Altieri VM, Verze P, Mirone V. Minilaparoendoscopic Single-site Pyeloplasty: The Best Compromise Between Surgeon's Ergonomy and Patient's Cosmesis (IDEAL Phase 2a). European Urology Focus. 2016;2(3):319-26.
45. Rischmann P, Gelet A, Riche B, Villers A, Pasticier G, Bondil P, et al. Focal High Intensity Focused Ultrasound of Unilateral Localized Prostate Cancer: A Prospective Multicentric Hemiablation Study of 111 Patients. Eur Urol. 2017;71(2):267-73.
46. Breda A, Territo A, Guttilla A, Sanguedolce F, Manfredi M, Quaresima L, et al. Correlation Between Confocal Laser Endomicroscopy (Cellvizio(®)) and Histological Grading of Upper Tract Urothelial Carcinoma: A Step Forward for a Better Selection of Patients Suitable for Conservative Management. Eur Urol Focus. 2018;4(6):954-9.
47. Bus MTJ, de Bruin DM, Faber DJ, Kamphuis GM, Zondervan PJ, Laguna-Pes MP, et al. Optical Coherence Tomography as a Tool for In Vivo Staging and Grading of Upper Urinary Tract Urothelial Carcinoma: A Study of Diagnostic Accuracy. J Urology. 2016;196(6):1749-55.

For peer review only - http://bmjopen.bmj.com/site/about/guidelines.xhtml

48. Graff P, Portalez D, Lusque A, Brun T, Aziza R, Khalifa J, et al. IDEAL 2a Phase II Study of Ultrafocal Brachytherapy for Low- and Intermediate-risk Prostate Cancer. Int J Radiat Oncol. 2018;102(4):903-11.
49. Liem EIML, Freund JE, Baard J, de Bruin DM, Pes MPL, Savci-Heijink CD, et al. Confocal Laser Endomicroscopy for the Diagnosis of Urothelial Carcinoma in the Bladder and the Upper Urinary Tract: Protocols for Two Prospective Explorative Studies. Jmir Res Protoc. 2018;7(2).
50. Swaan A, Mannaerts CK, Scheltema MJV, Nieuwenhuijzen JA, Savci-Heijink CD, de la Rosette JJMCH, et al. Confocal Laser Endomicroscopy and Optical Coherence Tomography for the Diagnosis of Prostate Cancer: A Needle-Based, In Vivo Feasibility Study Protocol (IDEAL Phase 2A). Jmir Res Protoc. 2018;7(5).
51. Blazeby JM, Blencowe NS, Titcomb DR, Metcalfe C, Hollowood AD, Barham CP. Demonstration of the IDEAL recommendations for evaluating and reporting surgical innovation in minimally invasive oesophagectomy. Brit J Surg. 2011;98(4):544-51.
52. Dumont F, Duchalais E, Aumont A, Thibaudeau E. Cytoreductive surgery plus hyperthermic intraperitoneal chemotherapy by laparoscopy via a single-port approach for low-grade peritoneal malignancy. Surgical Endoscopy and Other Interventional Techniques. 2020;34(6):2789-95.
53. Goh BKP, Prieto M, Syn N, Koh YX, Lim KI. Critical appraisal of the learning curve of minimally invasive hepatectomy: experience with the first 200 cases of a Southeast Asian early adopter. Anz Journal of Surgery. 2020;90(6):1092-8.
54. Inoue Y, Saiura A, Sato T, Ishizawa T, Arita J, Takahashi Y, et al. Laparoscopic pancreatoduodenectomy combined with a novel self-assessment system and feedback discussion: a phase 1 surgical trial following the IDEAL guidelines. Langenbeck Arch Surg. 2016;401(8):1123-30.
55. Stenstra MHBC, van Workum F, van den Wildenberg FJH, Polat F, Rosman C. Evolution of the surgical technique of minimally invasive Ivor-Lewis esophagectomy: description according to the IDEAL framework. Diseases of the Esophagus. 2019;32(3).
56. Gerullis H, Ecke TH, Barski D, Bantel C, Weyland A, Uphoff J, et al. Retrospective analysis of a surgical innovation using the IDEAL framework: radical cystectomy with epidural anaesthesia. J Int Med Res. 2017;45(2):714-22.
57. Gerullis H, Barski D, Georgas E, Boros M, Ramon A, Ecke TH, et al. Protocol for a Randomized Phase II Trial for Mesh Optimization by Autologous Plasma Coating in Prolapse Repair: IDEAL Stage 3. Adv Ther. 2017;34(4):995-1006.
58. Gaboardi F, Pini G, Suardi N, Montorsi F, Passaretti G, Smelzo S. Robotic laparoendoscopic single-site radical prostatectomy (R-LESS-RP) with daVinci Single-Site (R) platform. Concept and evolution of the technique following an IDEAL phase 1. J Robot Surg. 2019;13(2):215-26.
59. Kaouk JH, Haber GP, Autorino R, Crouzet S, Ouzzane A, Flamand V, et al. A Novel Robotic System for Single-port Urologic Surgery: First Clinical Investigation. Eur Urol. 2014;66(6):1033-43.
60. del Val ID, Loureiro C, McCulloch P. The IDEAL prospective development study format for reporting surgical innovations. An illustrative case study of robotic oesophagectomy. International Journal of Surgery. 2015;19:104-11.
61. Barski D, Arndt C, Gerullis H, Yang J, Boros M, Otto T, et al. Transvaginal PVDF-mesh for cystocele repair: A cohort study. International Journal of Surgery. 2017;39:249-54.
62. Versteeg AL, van der Velden JM, Hes J, Eppinga W, Kasperts N, Verkooijen HM, et al. Stereotactic Radiotherapy Followed by Surgical Stabilization Within 24 h for Unstable Spinal Metastases; A Stage I/IIa Study According to the IDEAL Framework. Front Oncol. 2018;8.
63. Mannaerts CK, Wildeboer RR, Postema AW, Hagemann J, Budaus L, Tilki D, et al. Multiparametric ultrasound: evaluation of greyscale, shear wave elastography and contrast-enhanced ultrasound for prostate cancer detection and localization in correlation to radical prostatectomy specimens. Bmc Urol. 2018;18.
64. Barentsz MW, Verkooijen HM, Pijnappel RM, Fernandez MA, van Diest PJ, van der Pol CC, et al. Sentinel lymph node localization with contrast-enhanced ultrasound and an I-125 seed: An ideal prospective development study. International Journal of Surgery. 2015;14:1-6.

For peer review only - http://bmjopen.bmj.com/site/about/guidelines.xhtml

65. Chan JYK, Tsang RK, Holsinger FC, Tong MCF, Ng CWK, Chiu PWY, et al. Prospective clinical trial to evaluate safety and feasibility of using a single port flexible robotic system for transoral head and neck surgery. Oral Oncol. 2019;94:101-5.
66. Hallmann S, Petersein J, Ruttloff J, Ecke TH. Successful evacuation of large perirenal hematoma after extracorporeal shock wave lithotripsy (ESWL) - step 1 of the IDEAL recommendations of surgical innovation. Clin Case Rep. 2017;5(2):123-5.
67. Valerio M, Dickinson L, Ali A, Ramachandran N, Donaldson I, Freeman A, et al. A prospective development study investigating focal irreversible electroporation in men with localised prostate cancer: Nanoknife Electroporation Ablation Trial (NEAT). Contemporary clinical trials. 2014;39(1):57-65.
68. Gerullis H, Schwartmann K, Ecke TH, Georgas E, Herberg P, Winter A, et al. Transvesical Suprapubic Externalization of Ureteral Stents - Introduction of a Surgical Innovation at the Development Stage. Urol Int. 2017;99(1):69-76.
69. Currie AC, Blazeby JM, Suzuki N, Thomas-Gibson S, Reeves B, Morton D, et al. Evaluation of an early-stage innovation for full-thickness excision of benign colonic polyps using the IDEAL framework. Colorectal Disease. 2019;21(9):1004-16.
70. Sharma SK, Momose K, Sedrakyan A, Sonoda T, Sharaiha RZ. Endoscopic stabilization device evaluation using IDEAL framework: A quality improvement study. International Journal of Surgery. 2019;67:18-23.
71. Menon M, Abaza R, Sood A, Ahlawat R, Ghani KR, Jeong W, et al. Robotic Kidney Transplantation with Regional Hypothermia: Evolution of a Novel Procedure Utilizing the IDEAL Guidelines (IDEAL Phase 0 and 1). Eur Urol. 2014;65(5):1001-9.
72. Sood A, McCulloch P, Dahm P, Ahlawat R, Jeong WJ, Bhandari M, et al. Ontogeny of a surgical technique: Robotic kidney transplantation with regional hypothermia. International Journal of Surgery. 2016;25:158-61.
73. Banagala S, Jayarajah U, Almeida I, Samarasekera DN. Efficacy of irrigation tubes in the management of para rectal cavities associated with complex fistula-in-ano. Bmc Surgery. 2018;18.
74. van Kollenburg RAA, van Riel LAMJG, Bloemen PR, Oddens JR, de Reijke TM, Beerlage HP, et al. Transperineal Laser Ablation Treatment for Lower Urinary Tract Symptoms Due to Benign Prostatic Obstruction: Protocol for a Prospective In Vivo Pilot Study. Jmir Res Protoc. 2020;9(1).
75. Harvey KL, Mills N, White P, Holcombe C, Potter S, Steering P-BFS. The Pre-BRA (pre-pectoral Breast Reconstruction EvAluation) feasibility study: protocol for a mixed-methods IDEAL 2a/2b prospective cohort study to determine the safety and effectiveness of prepectoral implant-based breast reconstruction. Bmj Open. 2020;10(1).
76. Buijs M, van Lienden KP, Wagstaff PGK, Scheltema MJV, de Bruin DM, Zondervan PJ, et al. Irreversible Electroporation for the Ablation of Renal Cell Carcinoma: A Prospective, Human, In Vivo Study Protocol (IDEAL Phase 2b). Jmir Res Protoc. 2017;6(2).
77. Villers A, Puech P, Flamand V, Haber GP, Desai MM, Crouzet S, et al. Partial Prostatectomy for Anterior Cancer: Short-term Oncologic and Functional Outcomes. Eur Urol. 2017;72(3):333-42.
78. Barski D, Gerullis H, Ecke T, Varga G, Boros M, Pintelon I, et al. Repair of a vesico-vaginal fistula with amniotic membrane - Step 1 of the IDEAL recommendations of surgical innovation. Cent Eur J Urol. 2015;68(4):459-61.
79. Joukhadar R, Wockel A, Herr D, Paulus V, Radosa J, Hamza A, et al. Challenges of Longevity: Safety of Vaginal and Laparoscopic Urogynecological Procedures in Septuagenarians and Older Patients. Biomed Res Int. 2016;2016.
80. Heikens JT, Gooszen HG, Rovers MM, van Laarhoven CJHM. Stages and Evaluation of Surgical Innovation: A Clinical Example of the Ileo Neorectal Anastomosis After Ulcerative Colitis and Familial Adenomatous Polyposis. Surgical Innovation. 2013;20(5):459-65.
81. Saglam R, Muslumanoglu AY, Tokath Z, Caskurlu T, Sarica K, Tasci AI, et al. A New Robot for Flexible Ureteroscopy: Development and Early Clinical Results (IDEAL Stage 1-2b). Eur Urol. 2014;66(6):1092-100.

For peer review only - http://bmjopen.bmj.com/site/about/guidelines.xhtml

82. Lotz M. Surgical innovation as sui generis surgical research. Theoretical medicine and bioethics. 2013;34(6):447-59.
83. Hasenboehler EA, Choudhry IK, Newman JT, Smith WR, Ziran BH, Stahel PF. Bias towards publishing positive results in orthopedic and general surgery: a patient safety issue? Patient Saf Surg. 2007;1(1):4.
84. Ross JS, Mulvey GK, Hines EM, Nissen SE, Krumholz HM. Trial publication after registration in ClinicalTrials.Gov: a cross-sectional analysis. PLoS Med. 2009;6(9):e1000144.
85. Song F, Parekh S, Hooper L, Loke YK, Ryder J, Sutton AJ, et al. Dissemination and publication of research findings: an updated review of related biases. Health Technol Assess. 2010;14(8):iii, ix-xi, 1-193.
86. Schekman R. How journals like Nature, Cell and Science are damaging science: The Guardian; 2013 [Available from: https://www.theguardian.com/commentisfree/2013/dec/09/how-journals-nature-science-cell-damage-science.
87. Vijayan J, Lachma RN, Mohan Rao PS, Bhat AS. Autologous pericardial aortic valve reconstruction: early results and comparison with mechanical valve replacement. Indian journal of thoracic and cardiovascular surgery. 2020;36(3):186-92.
88. Cousins S, Richards HS, Zahra J, Robertson H, Mathews JA, Avery KNL, et al. Healthcare organization policy recommendations for the governance of surgical innovation: review of NHS policies. Br J Surg. 2022;109(10):1004-12.
89. Stefanidis D, Fanelli RD, Price R, Richardson W, Committee SG. SAGES guidelines for the introduction of new technology and techniques. Surg Endosc. 2014;28(8):2257-71.
90. Surgeons RCo. Surgical Innovation, New Techniques and Technologies. 2019.
91. Surgeons RCo. Learning from invited reviews. 2019.
92. Elliott D, Blencowe NS, Cousins S, Zahra J, Skilton A, Mathews J, et al. Using qualitative research methods to understand how surgical procedures and devices are introduced into NHS hospitals: the Lotus study protocol. BMJ Open. 2021;11(12):e049234.
93. Siemeiniuk RG, G. . What is GRADE? 2023 [Available from: https://bestpractice.bmj.com/info/toolkit/learn-ebm/what-is-grade/.
94. Emamaullee J, Bowdish M, Yan PY, Rodina V, Sher LS. Rapid Adaptation of a Surgical Research Unit to Conduct Clinical Trials During the COVID-19 Pandemic. J Surg Res. 2020;256:76-82.
95. Lupkin S. Coronavirus pandemic Brings Hundreds of U.S. Clinical trials to A Halt. : Heal News from NPR; 2020 [Available from: https://www.npr.org/sections/health-shots/2020/04/11/832210606/coronovirus-pandemic-brings-hundreds-of-u-s-clinical-trials-to-a-halt.
96. Thornton J. Clinical trials suspended in UK to prioritise covid-19 studies and free up staff. Bmj-Brit Med J. 2020;368.
97. Glasbey JC, Ademuyiwa A, Adisa A, AlAmeer E, Arnaud AP, Ayasra F, et al. Effect of COVID-19 pandemic lockdowns on planned cancer surgery for 15 tumour types in 61 countries: an international, prospective, cohort study. Lancet Oncology. 2021;22(11):1507-17.
98. Stoss C, Steffani M, Kohlhaw K, Rudroff C, Staib L, Hartmann D, et al. The COVID-19 pandemic: impact on surgical departments of non-university hospitals. Bmc Surgery. 2020;20(1).

For peer review only - http://bmjopen.bmj.com/site/about/guidelines.xhtml

Supplementary file 1: ENTREQ and PRISMA Checklists

Enhancing transparency in reporting the synthesis of qualitative research (ENTREQ) statement items

No.	Item	Guide and description	Reported on page #
1	Aim	State the research question the synthesis addresses	4
2	Synthesis methodology	Identify the synthesis methodology or theoretical framework which underpins the synthesis, and describe the rationale for choice of methodology (e.g. metaethnography, thematic synthesis, critical interpretive synthesis, grounded theory synthesis, realist synthesis, meta-aggregation, meta-study, framework synthesis).	6-7
3	Approach to searching	Indicate whether the search was pre-planned (comprehensive search strategies to seek all available studies) or iterative (to seek all available concepts until they theoretical saturation is achieved).	4 and Supplementary File 2
4	Inclusion criteria	Specify the inclusion/exclusion criteria (e.g. in terms of population, language, year limits, type of publication, study type).	5
5	Data sources	Describe the information sources used (e.g. electronic databases (MEDLINE, EMBASE, CINAHL, psycINFO, Econlit), grey literature databases (digital thesis, policy reports), relevant organisational websites, experts, information specialists, generic web searches (Google Scholar) hand searching, reference lists) and when the searches conducted; provide the rationale for using the data sources.	4 and Supplementary File 2
6	Electronic Search strategy	Describe the literature search (e.g. provide electronic search strategies with population terms, clinical or health topic terms, experiential or social phenomena related terms, filters for qualitative research, and search limits).	Supplementary File 2
7	Study screening methods	Describe the process of study screening and sifting (e.g. title, abstract and full text review, number of independent reviewers who screened studies).	5
8	Study characteristics	Present the characteristics of the included studies (e.g. year of publication, country, population, number of participants, data collection, methodology, analysis, research questions).	9 & Table 1
9	Study selection results	Identify the number of studies screened and provide reasons for study exclusion (e,g, for comprehensive searching, provide numbers of studies screened and reasons for exclusion indicated in a figure/flowchart; for iterative searching describe reasons	7 & Figure 1

For peer review only

Supplementary file 1: ENTREQ and PRISMA Checklists

		for study exclusion and inclusion based on modifications t the research question and/or contribution to theory development).	
10	Rationale for appraisal	Describe the rationale and approach used to appraise the included studies or selected findings (e.g. assessment of conduct (validity and robustness), assessment of reporting (transparency), assessment of content and utility of the findings).	N/A
11	Appraisal items	State the tools, frameworks and criteria used to appraise the studies or selected findings (e.g. Existing tools: CASP, QARI, COREQ, Mays and Pope [25]; reviewer developed tools; describe the domains assessed: research team, study design, data analysis and interpretations, reporting).	N/A
12	Appraisal process	Indicate whether the appraisal was conducted independently by more than one reviewer and if consensus was required.	N/A
13	Appraisal results	Present results of the quality assessment and indicate which articles, if any, were weighted/excluded based on the assessment and give the rationale.	N/A
14	Data extraction	Indicate which sections of the primary studies were analysed and how were the data extracted from the primary studies? (e.g. all text under the headings "results /conclusions" were extracted electronically and entered into a computer software).	5-6
15	Software	State the computer software used, if any.	6
16	Number of reviewers	Identify who was involved in coding and analysis.	7
17	Coding	Describe the process for coding of data (e.g. line by line coding to search for concepts).	6
18	Study comparison	Describe how were comparisons made within and across studies (e.g. subsequent studies were coded into pre-existing concepts, and new concepts were created when deemed necessary).	7
19	Derivation of themes	Explain whether the process of deriving the themes or constructs was inductive or deductive.	6-7
20	Quotations	Provide quotations from the primary studies to illustrate themes/constructs, and identify whether the quotations were participant quotations of the author's interpretation.	13-17 & Table 2
21	Synthesis output	Present rich, compelling and useful results that go beyond a summary of the primary studies (e.g. new interpretation, models of evidence, conceptual models, analytical framework, development of a new theory or construct).	11-17

For peer review only

Supplementary file 1: ENTREQ and PRISMA Checklists

Section/topic	#	Checklist item	Reported on page #
**TITLE**			
Title	1	Identify the report as a systematic review, meta-analysis, or both.	1
**ABSTRACT**			
Structured summary	2	Provide a structured summary including, as applicable: background; objectives; data sources; study eligibility criteria, participants, and interventions; study appraisal and synthesis methods; results; limitations; conclusions and implications of key findings; systematic review registration number.	1
**INTRODUCTION**			
Rationale	3	Describe the rationale for the review in the context of what is already known.	4
Objectives	4	Provide an explicit statement of questions being addressed with reference to participants, interventions, comparisons, outcomes, and study design (PICOS).	N/A
**METHODS**			
Protocol and registration	5	Indicate if a review protocol exists, if and where it can be accessed (e.g., Web address), and, if available, provide registration information including registration number.	N/A
Eligibility criteria	6	Specify study characteristics (e.g., PICOS, length of follow-up) and report characteristics (e.g., years considered, language, publication status) used as criteria for eligibility, giving rationale.	5
Information sources	7	Describe all information sources (e.g., databases with dates of coverage, contact with study authors to identify additional studies) in the search and date last searched.	4
Search	8	Present full electronic search strategy for at least one database, including any limits used, such that it could be repeated.	Supplementary File 2
Study selection	9	State the process for selecting studies (i.e., screening, eligibility, included in systematic review, and, if applicable, included in the meta-analysis).	5
Data collection process	10	Describe method of data extraction from reports (e.g., piloted forms, independently, in duplicate) and any processes for obtaining and confirming data from investigators.	5-7
Data items	11	List and define all variables for which data were sought (e.g., PICOS, funding sources) and any assumptions and simplifications made.	9-17

For peer review only - http://bmjopen.bmj.com/site/about/guidelines.xhtml

Supplementary file 1: ENTREQ and PRISMA Checklists

Risk of bias in individual studies	12	Describe methods used for assessing risk of bias of individual studies (including specification of whether this was done at the study or outcome level), and how this information is to be used in any data synthesis.	N/A
Summary measures	13	State the principal summary measures (e.g., risk ratio, difference in means).	N/A
Synthesis of results	14	Describe the methods of handling data and combining results of studies, if done, including measures of consistency (e.g., I^2) for each meta-analysis.	N/A
Risk of bias across studies	15	Specify any assessment of risk of bias that may affect the cumulative evidence (e.g., publication bias, selective reporting within studies).	N/A
Additional analyses	16	Describe methods of additional analyses (e.g., sensitivity or subgroup analyses, meta-regression), if done, indicating which were pre-specified.	N/A
**RESULTS**			
Study selection	17	Give numbers of studies screened, assessed for eligibility, and included in the review, with reasons for exclusions at each stage, ideally with a flow diagram.	Figure 1
Study characteristics	18	For each study, present characteristics for which data were extracted (e.g., study size, PICOS, follow-up period) and provide the citations.	9-17
Risk of bias within studies	19	Present data on risk of bias of each study and, if available, any outcome level assessment (see item 12).	N/A
Results of individual studies	20	For all outcomes considered (benefits or harms), present, for each study: (a) simple summary data for each intervention group (b) effect estimates and confidence intervals, ideally with a forest plot.	N/A
Synthesis of results	21	Present results of each meta-analysis done, including confidence intervals and measures of consistency.	N/A
Risk of bias across studies	22	Present results of any assessment of risk of bias across studies (see Item 15).	N/A
Additional analysis	23	Give results of additional analyses, if done (e.g., sensitivity or subgroup analyses, meta-regression [see Item 16]).	N/A
**DISCUSSION**			
Summary of evidence	24	Summarize the main findings including the strength of evidence for each main outcome; consider their relevance to key groups (e.g., healthcare providers, users, and policy makers).	17-19
Limitations	25	Discuss limitations at study and outcome level (e.g., risk of bias), and at review-level (e.g., incomplete retrieval of identified research, reporting bias).	19-20
Conclusions	26	Provide a general interpretation of the results in the context of other evidence, and implications for future research.	19-20

For peer review only

Supplementary file 1: ENTREQ and PRISMA Checklists

**FUNDING**			
Funding	27	Describe sources of funding for the systematic review and other support (e.g., supply of data); role of funders for the systematic review.	20

For peer review only

Supplementary Material 2

Search strategy:

The search strategy was to identify article that had cited at least one of the ten key IDEAL publications (see below) using the electronic citation tools in SCOPUS and Web of Science.

Electronic searches in these databases (web of Science and Scopus) were performed to identify studies reported as following the IDEAL/IDEAL-D framework. This was defined as a study citing any of the ten key IDEAL/IDEAL-D papers that members of the IDEAL collaboration deemed significant in describing the framework (see Khachane *et al*, 2018). No restrictions to study design or publication dates were applied. Searches were conducted in April 2019, and were updated in June 2020.

SCOPUS
Search date: 1st April 2019
1112 records identified.

Searches repeated: 26th June 2020 (limited to since 01/04/2019)
259 records identified.

Web of Science
Search date: 1st April 2019
1018 records identified.

Searches repeated: 26th June 2020 (limited to since 01/04/2019)
200 records identified.

Citation searches were conducted for the following papers:

1. McCulloch P, Altman DG, Campbell WB, Flum DR, Glasziou P, Marshall JC et al. No surgical innovation without evaluation: the IDEAL recommendations. Lancet 2009; 374: 1105–1112.
2. Sedrakyan A, Campbell B, Merino JG, Kuntz R, Hirst A, McCulloch P. IDEAL-D: a rational framework for evaluating and regulating the use of medical devices. BMJ 2016; 353: i2372.
3. Barkun JS, Aronson JK, Feldman LS, Maddern GJ, Strasberg SM, Altman DG et al. Evaluation and stages of surgical innovations. Lancet 2009; 374: 1089–1096.
4. Ergina PL, Cook JA, Blazeby JM, Boutron I, Clavien PA, Reeves BC et al. Challenges in evaluating surgical innovation. Lancet 2009; 374: 1097–1104.
5. Cook JA, McCulloch P, Blazeby JM, Beard DJ, Marinac-Dabic D, Sedrakyan A; IDEAL Group. IDEAL framework for surgical innovation 3: randomised controlled trials in the assessment stage and evaluations in the long term study stage. BMJ 2013; 346: f2820.
6. Ergina PL, Barkun JS, McCulloch P, Cook JA, Altman DG; IDEAL Group. IDEAL framework for surgical innovation 2: observational studies in the exploration and assessment stages. BMJ 2013; 346: f3011.
7. Hirst A, Agha RA, Rosin D, McCulloch P. How can we improve surgical research and innovation?: the IDEAL framework for action. Int J Surg 2013; 11: 1038–1042.

For peer review only

8. McCulloch P, Cook JA, Altman DG, Heneghan C, Diener MK; IDEAL Group. IDEAL framework for surgical innovation 1: the idea and development stages. BMJ 2013; 346: f3012.
9. Pennell CP, Hirst A, Sedrakyan A, McCulloch PG. Adapting the IDEAL framework and recommendations for medical device evaluation: a modified Delphi survey. Int J Surg 2016; 28: 141–148.
10. Pennell CP, Hirst AD, Campbell WB, Sood A, Agha RA, Barkun JS et al. Practical guide to the idea, development and exploration stages of the IDEAL framework and recommendations. Br J Surg 2016; 103: 607–615.

For peer review only

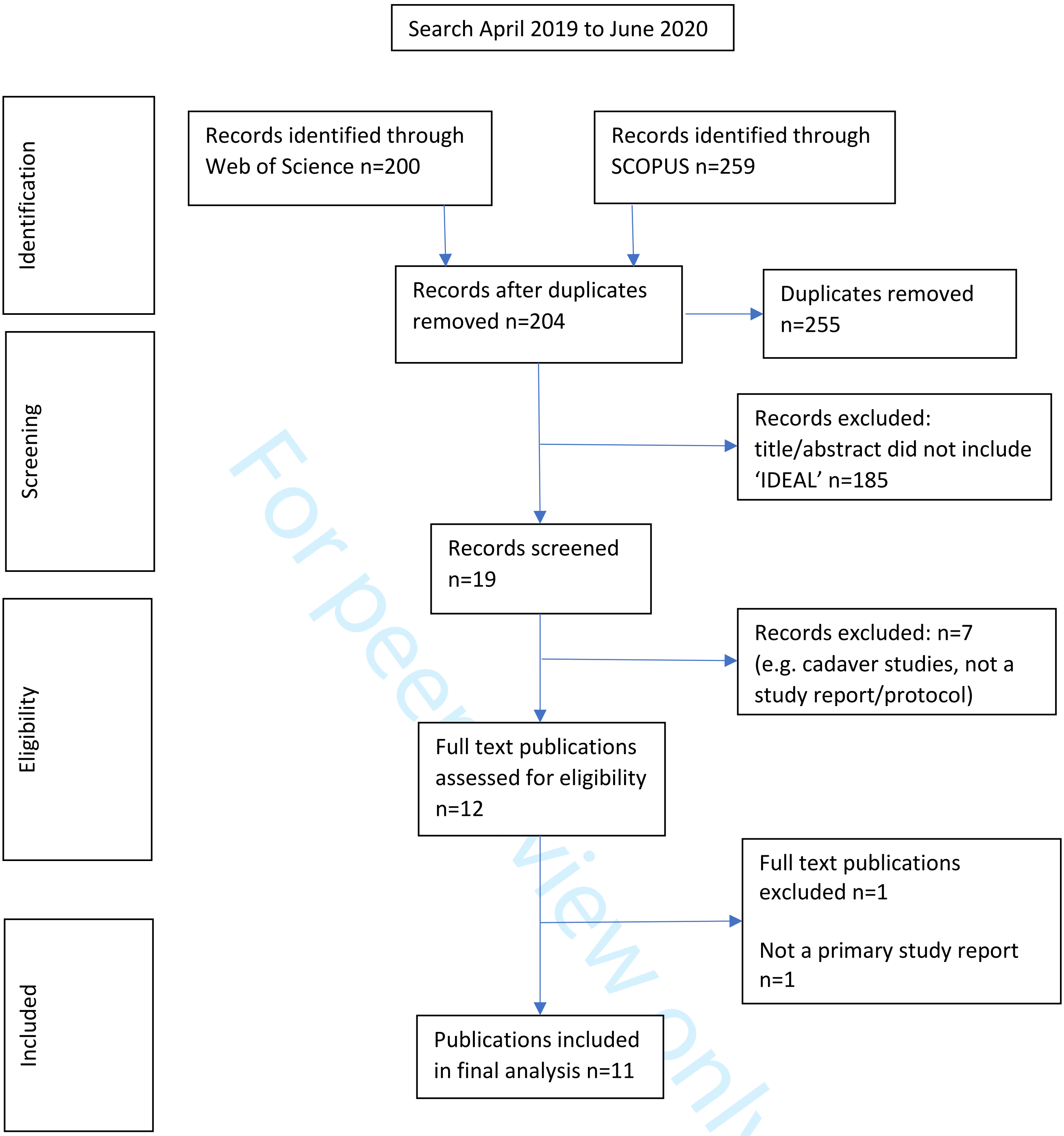

For peer review only
